# Supplementary figures and images for: Coupling to short linear motifs creates versatile PME-1 activities in PP2A holoenzyme demethylation and inhibition
Source: eLife. 2022 Aug 4;11:e79736. doi: 10.7554/eLife.79736 (PMC9398451; doi:10.7554/eLife.79736)

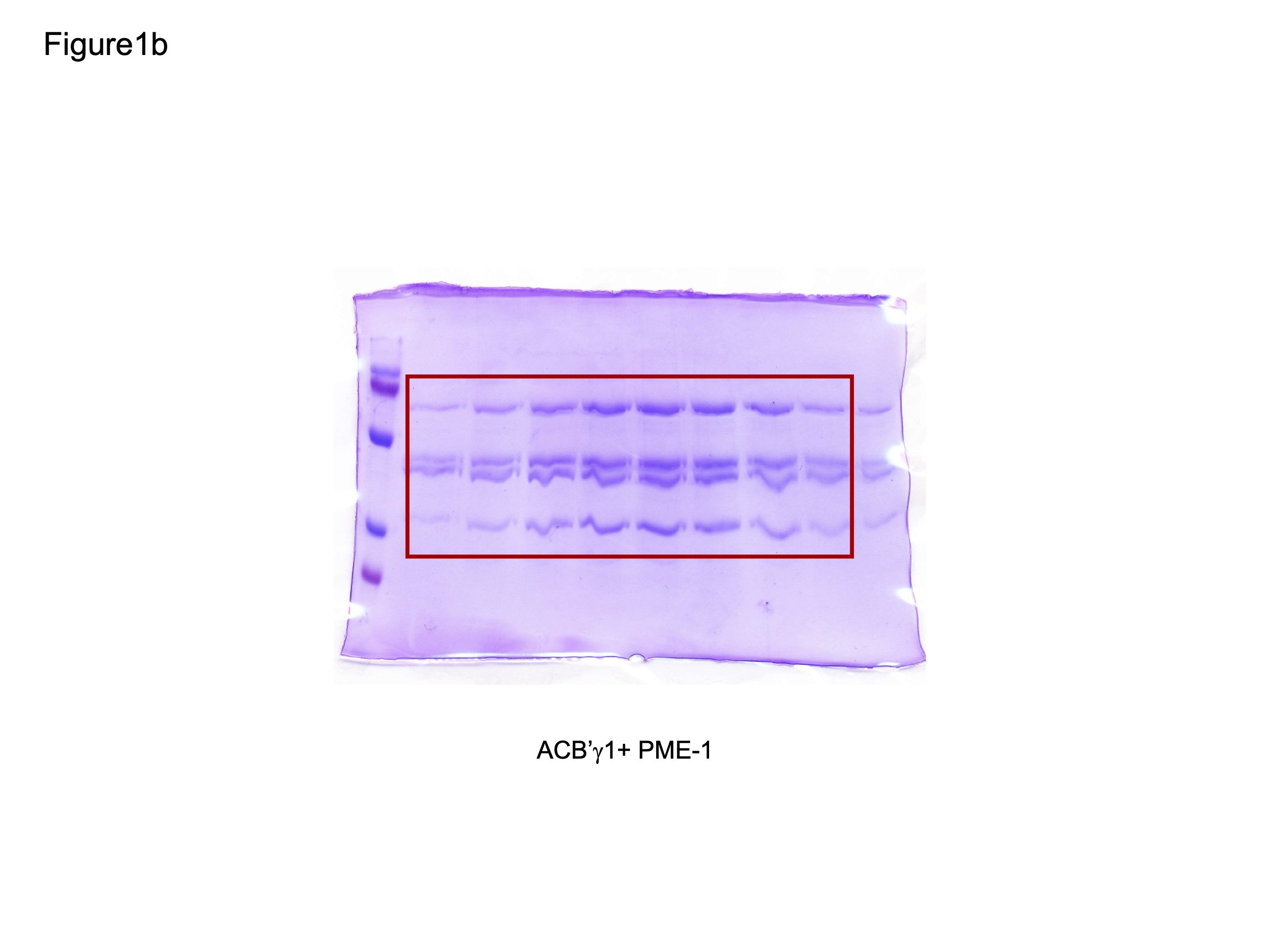

Supplement: Figure 1—source data 1. [file elife-79736-fig1-data1.zip › Figure 1-source data 1/Uncropped_Labeled_Gel_Figure 1b.jpg]

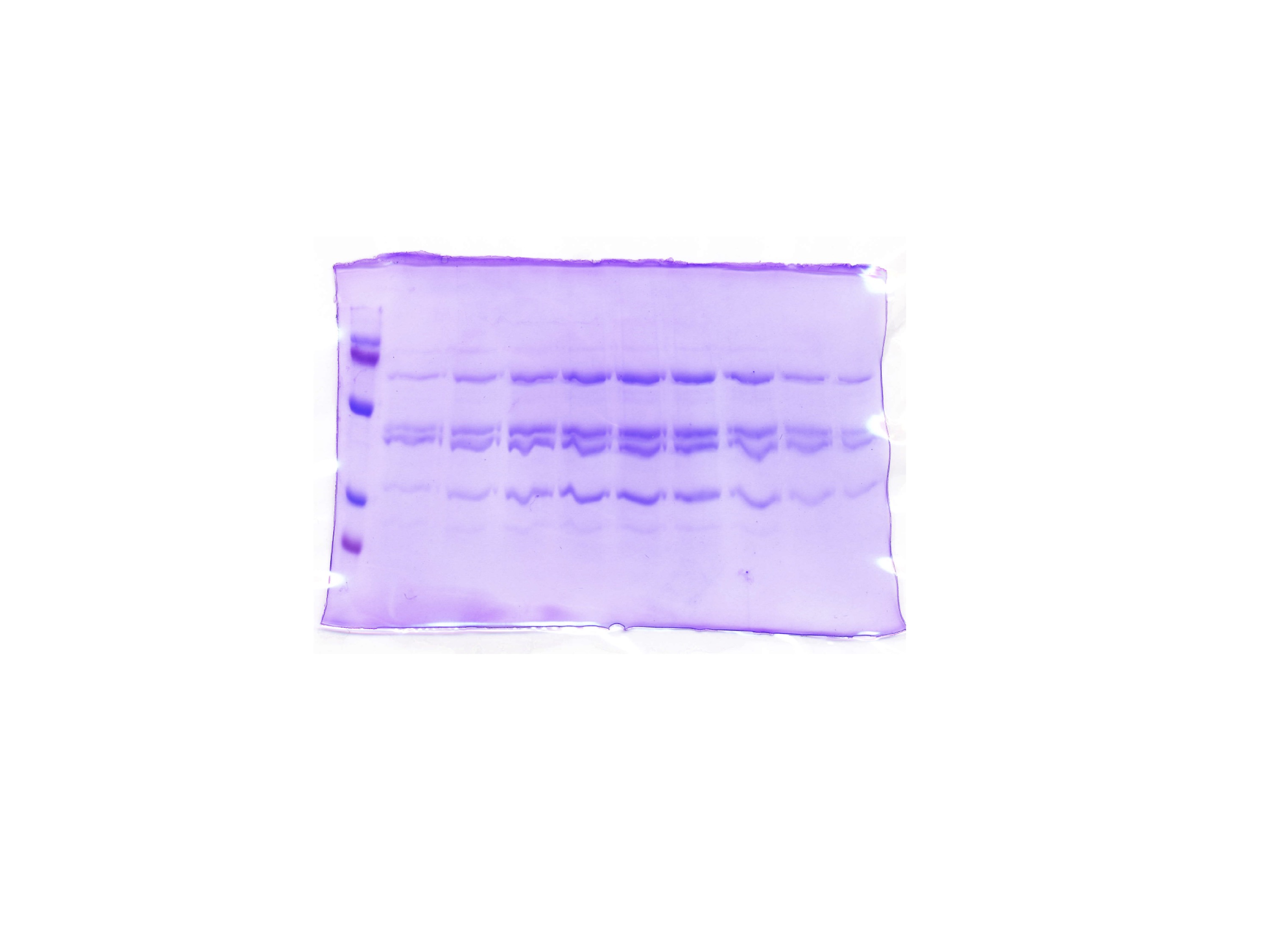

Supplement: Figure 1—source data 1. [file elife-79736-fig1-data1.zip › Figure 1-source data 1/Figure 1b.jpg]

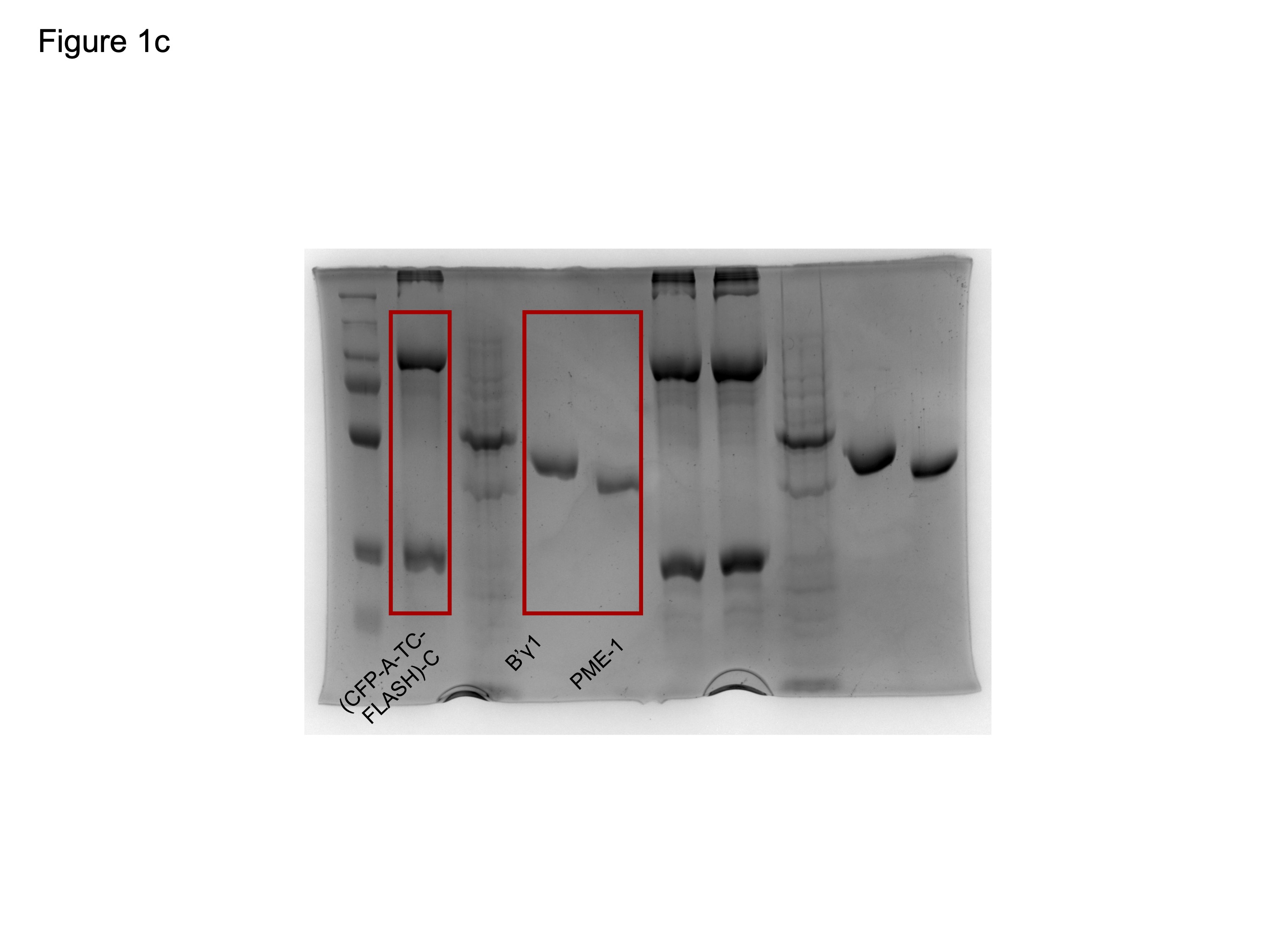

Supplement: Figure 1—source data 2. [file elife-79736-fig1-data2.zip › Figure 1-source data 2/Uncropped_Labeled_Gel_Figure 1c.jpg]

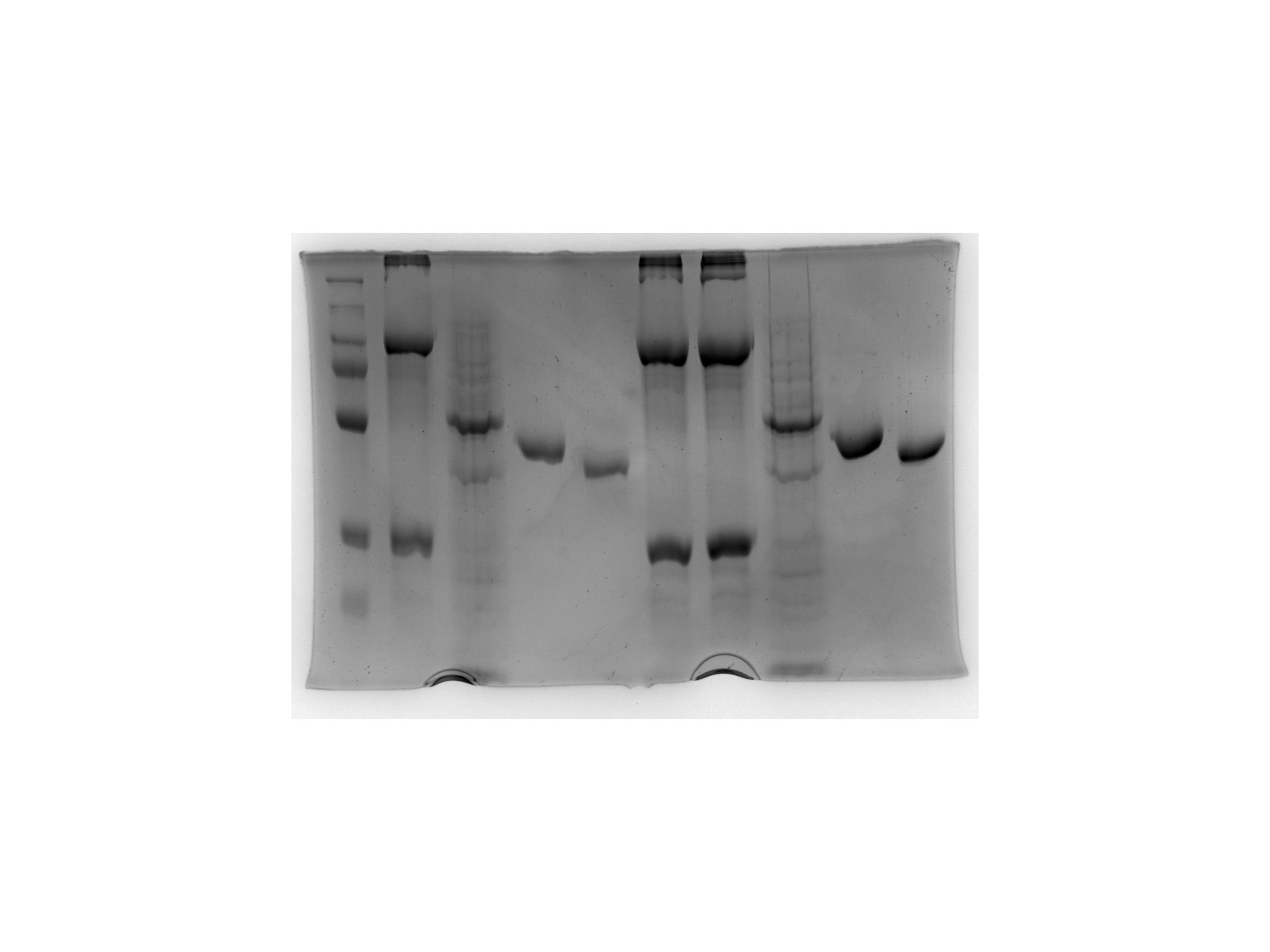

Supplement: Figure 1—source data 2. [file elife-79736-fig1-data2.zip › Figure 1-source data 2/Figure 1c.jpg]

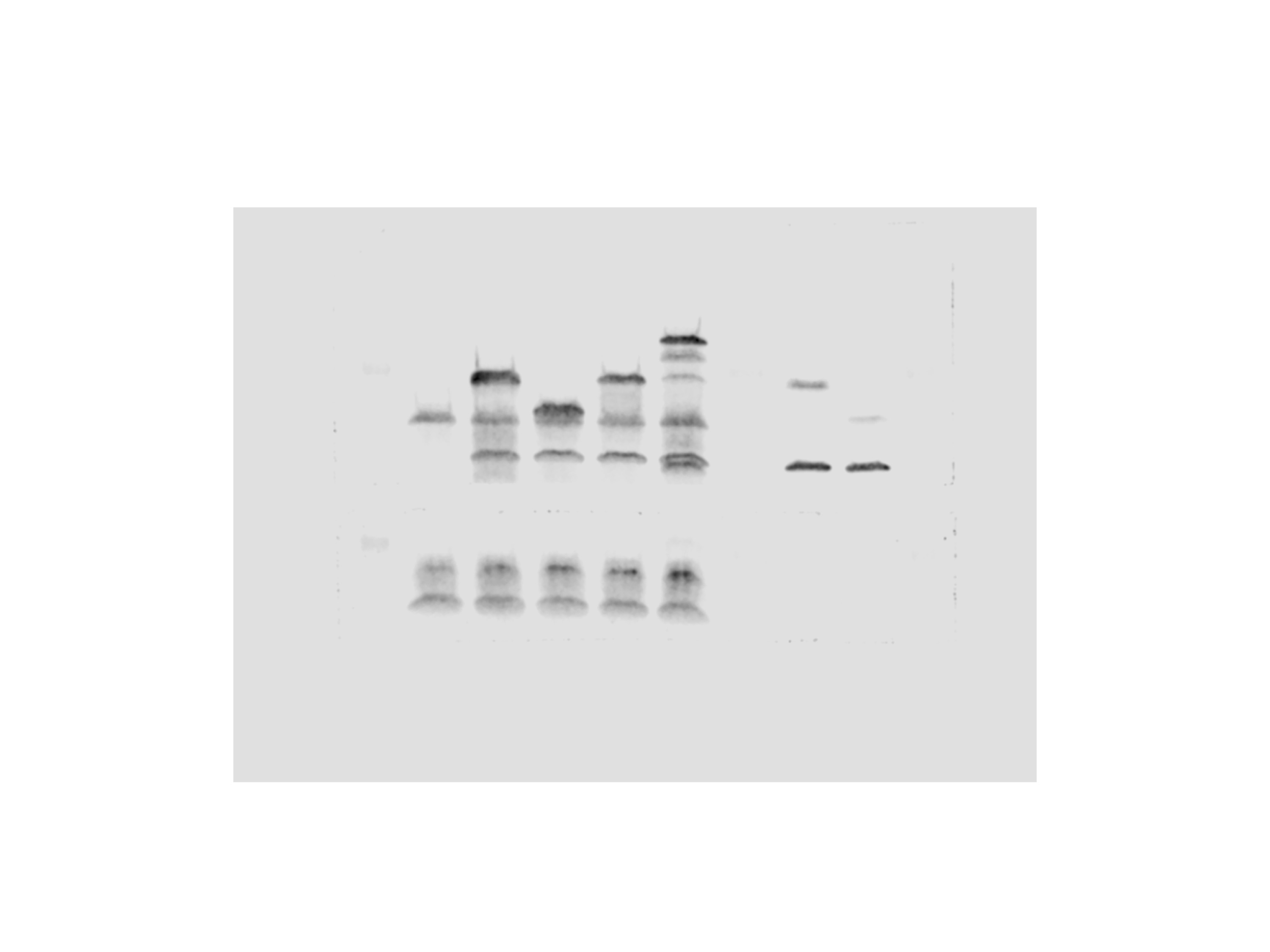

Supplement: Figure 1—source data 3. [file elife-79736-fig1-data3.zip › Figure 1-source data 3/Figure 1d_IP_B56.jpg]

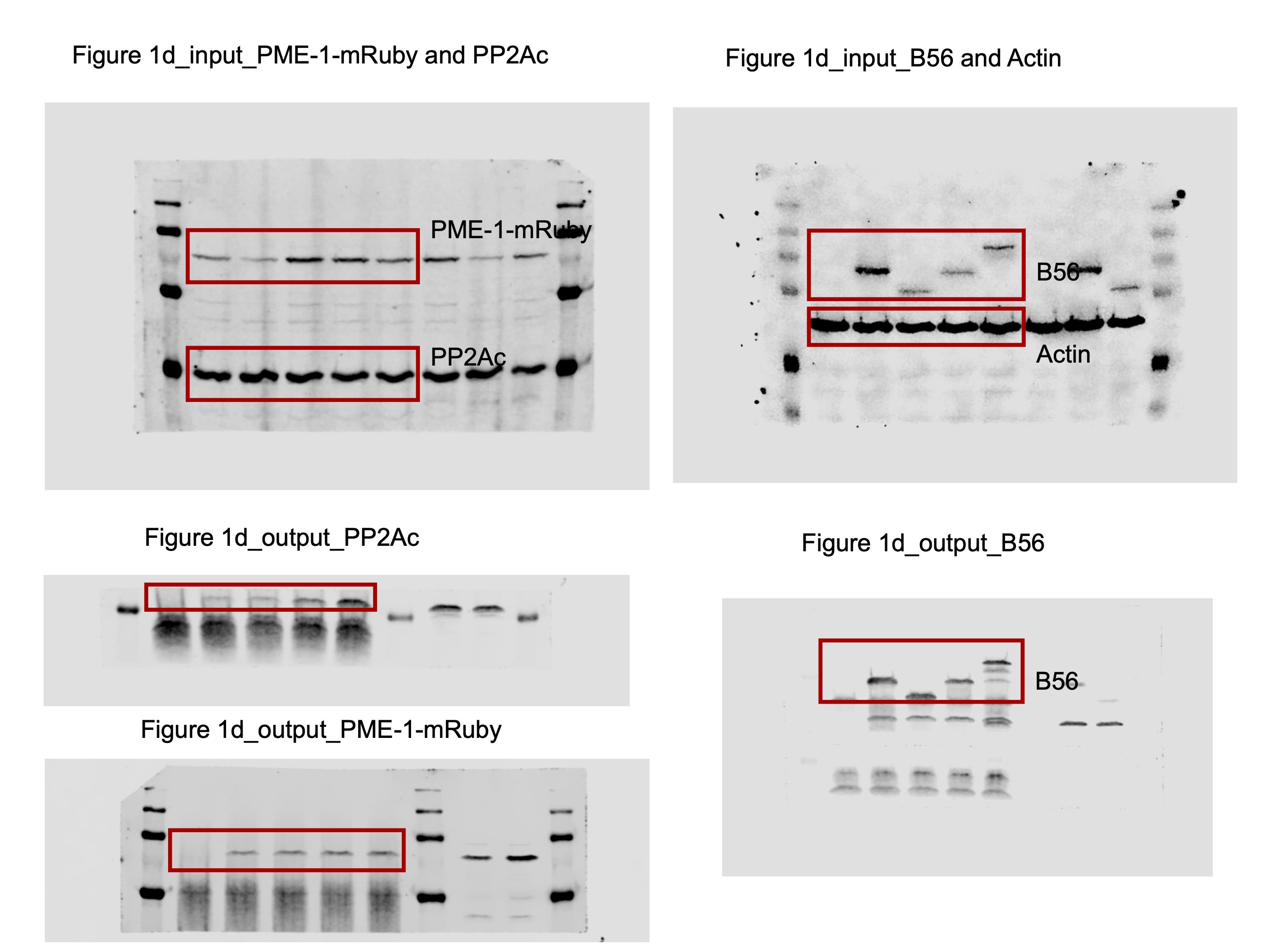

Supplement: Figure 1—source data 3. [file elife-79736-fig1-data3.zip › Figure 1-source data 3/Uncropped_Labeled_Gel_Figure 1d.jpg]

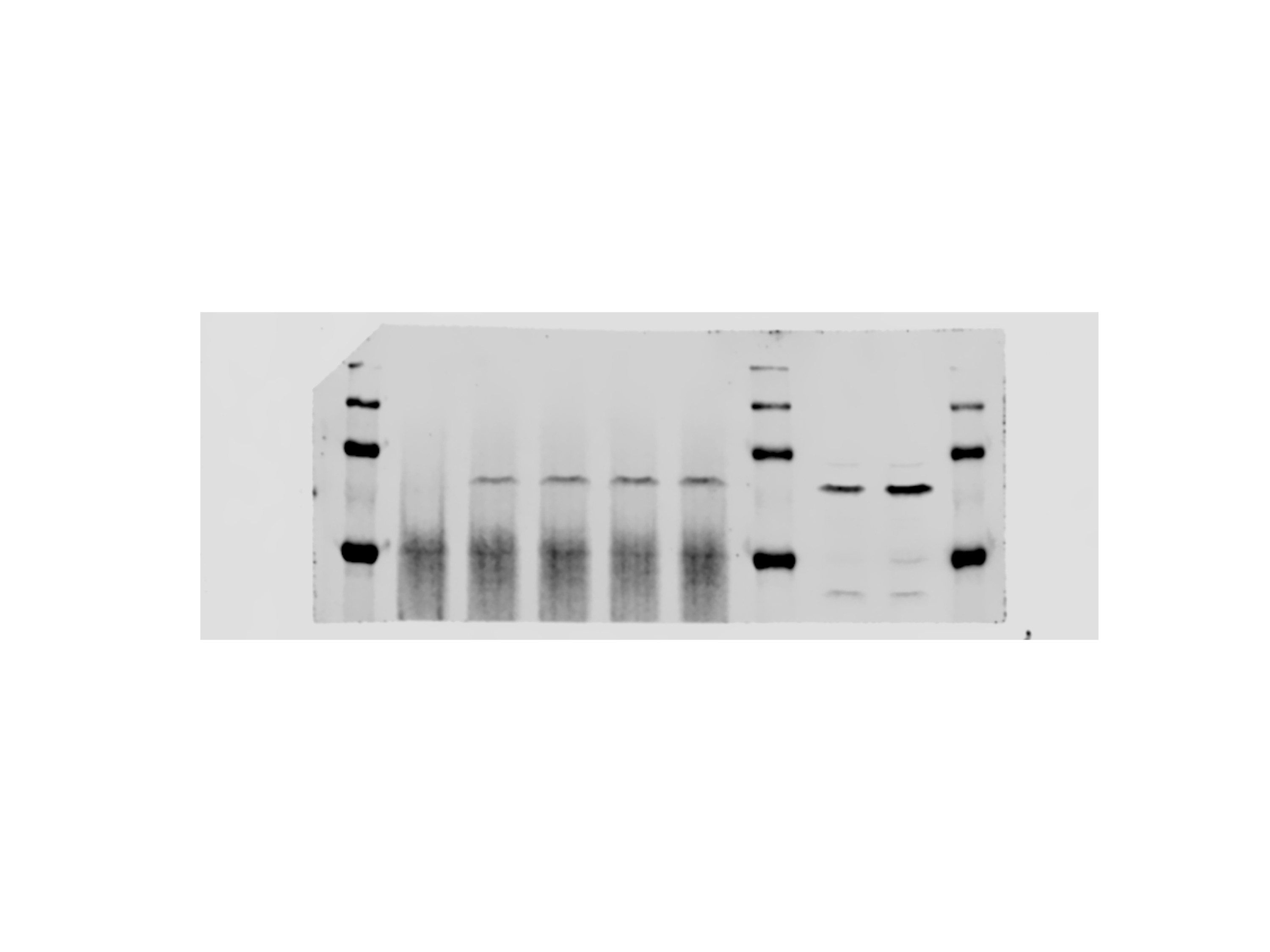

Supplement: Figure 1—source data 3. [file elife-79736-fig1-data3.zip › Figure 1-source data 3/Figure 1d_IP_PME-1-mRuby.jpg]

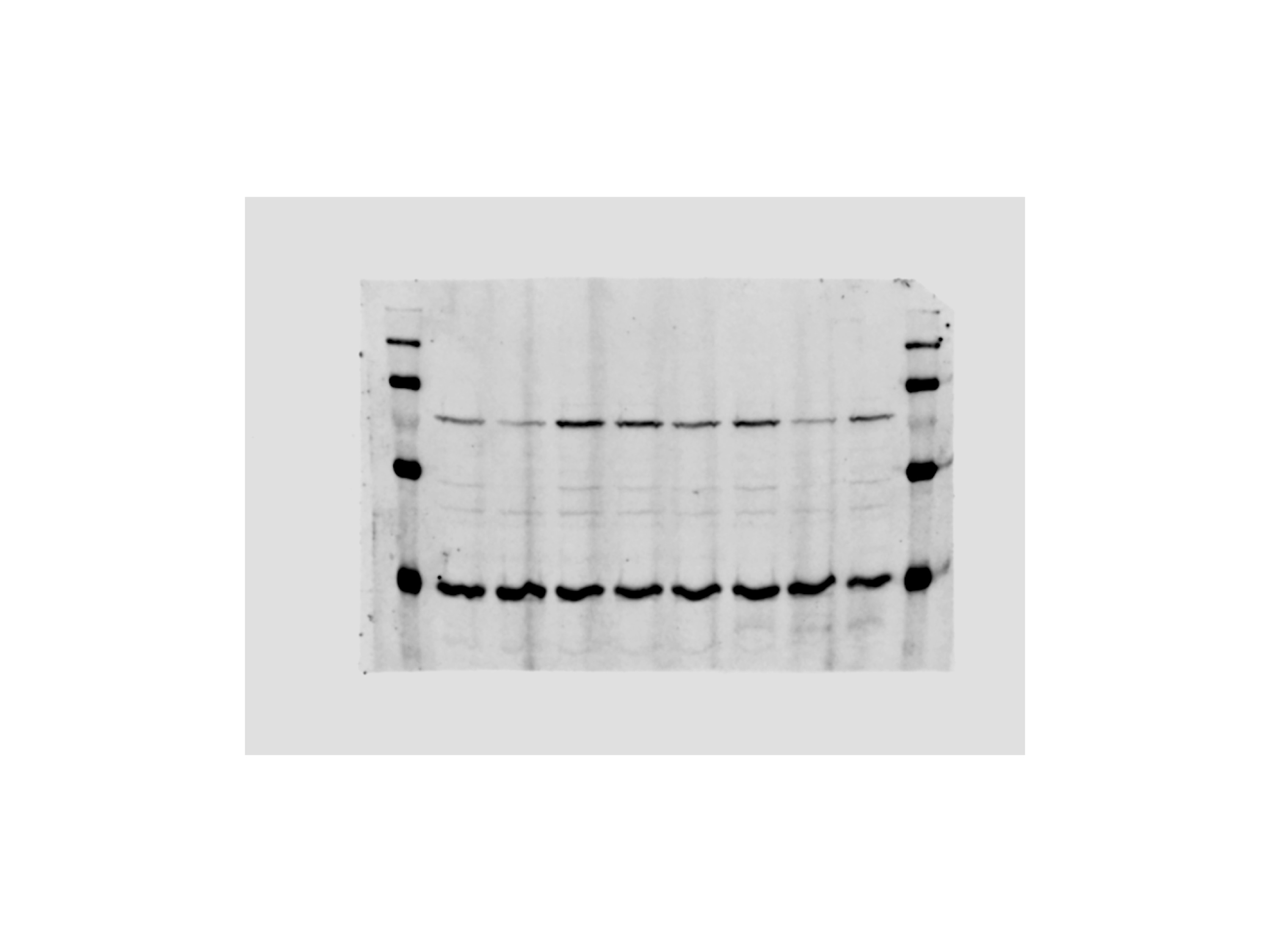

Supplement: Figure 1—source data 3. [file elife-79736-fig1-data3.zip › Figure 1-source data 3/Figure 1d_input_PME-1-mRuby and PP2Ac.jpg]

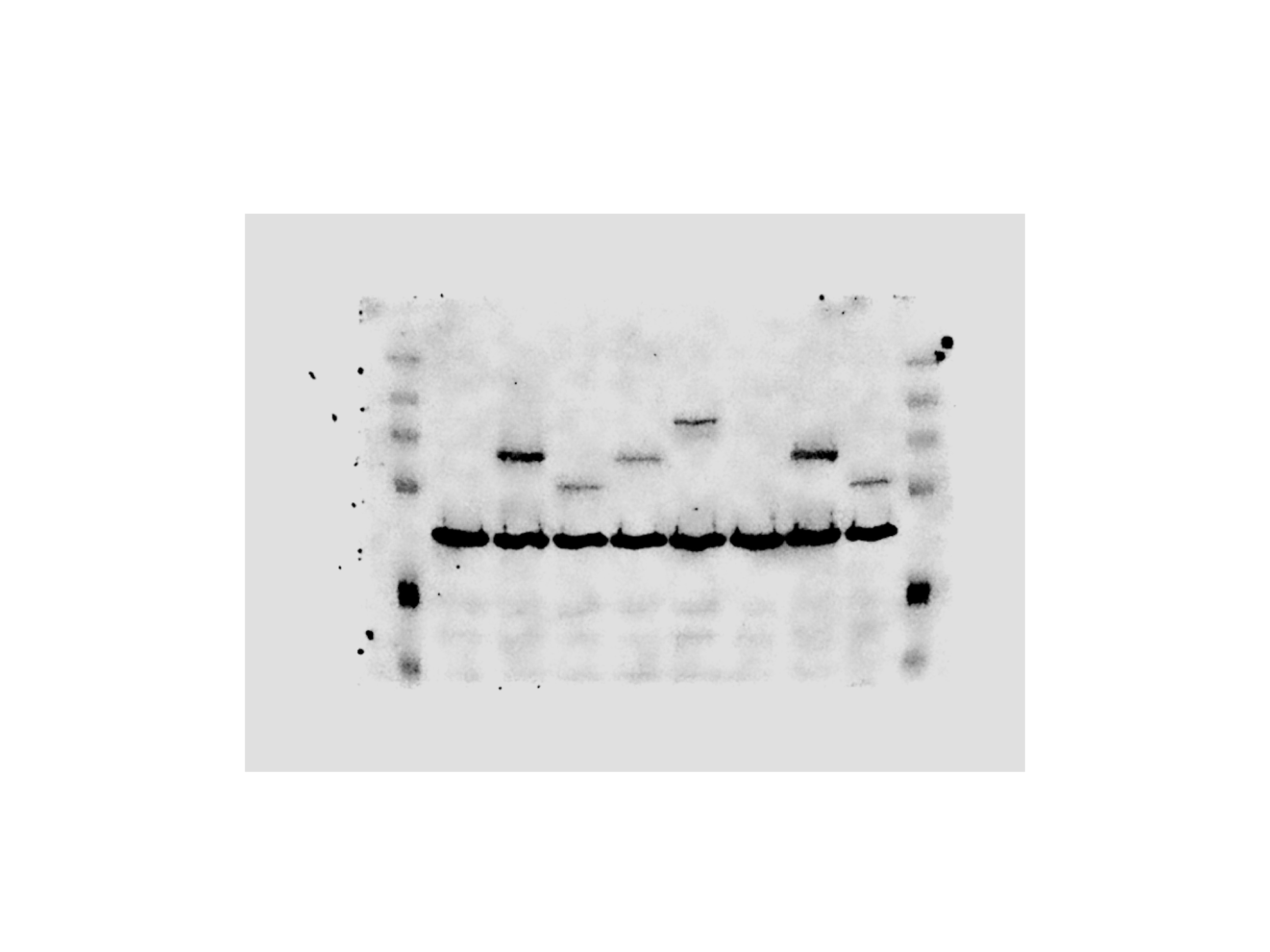

Supplement: Figure 1—source data 3. [file elife-79736-fig1-data3.zip › Figure 1-source data 3/Figure 1d_input_B56 and Actin.jpg]

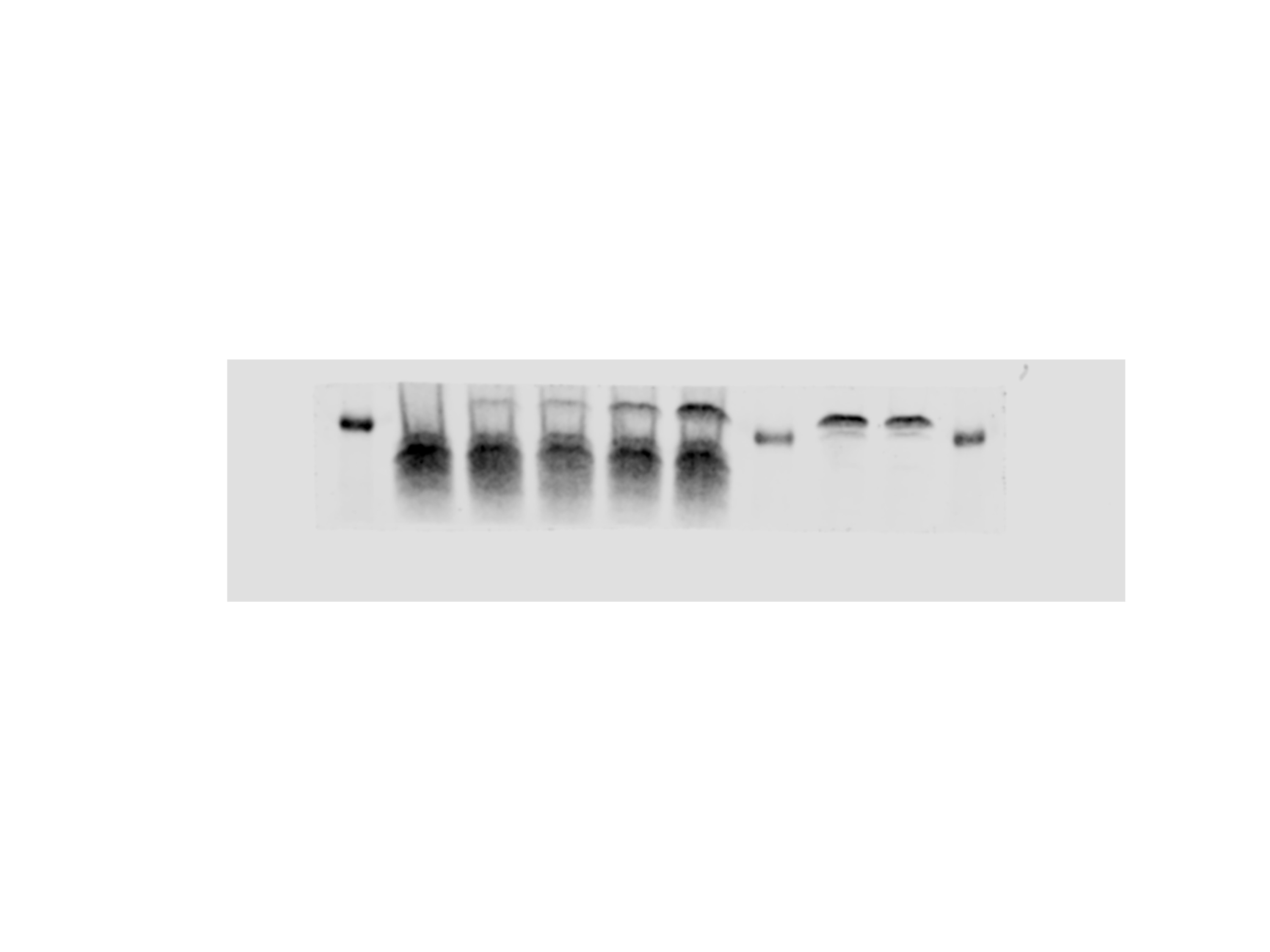

Supplement: Figure 1—source data 3. [file elife-79736-fig1-data3.zip › Figure 1-source data 3/Figure 1d_IP_PP2Ac.jpg]

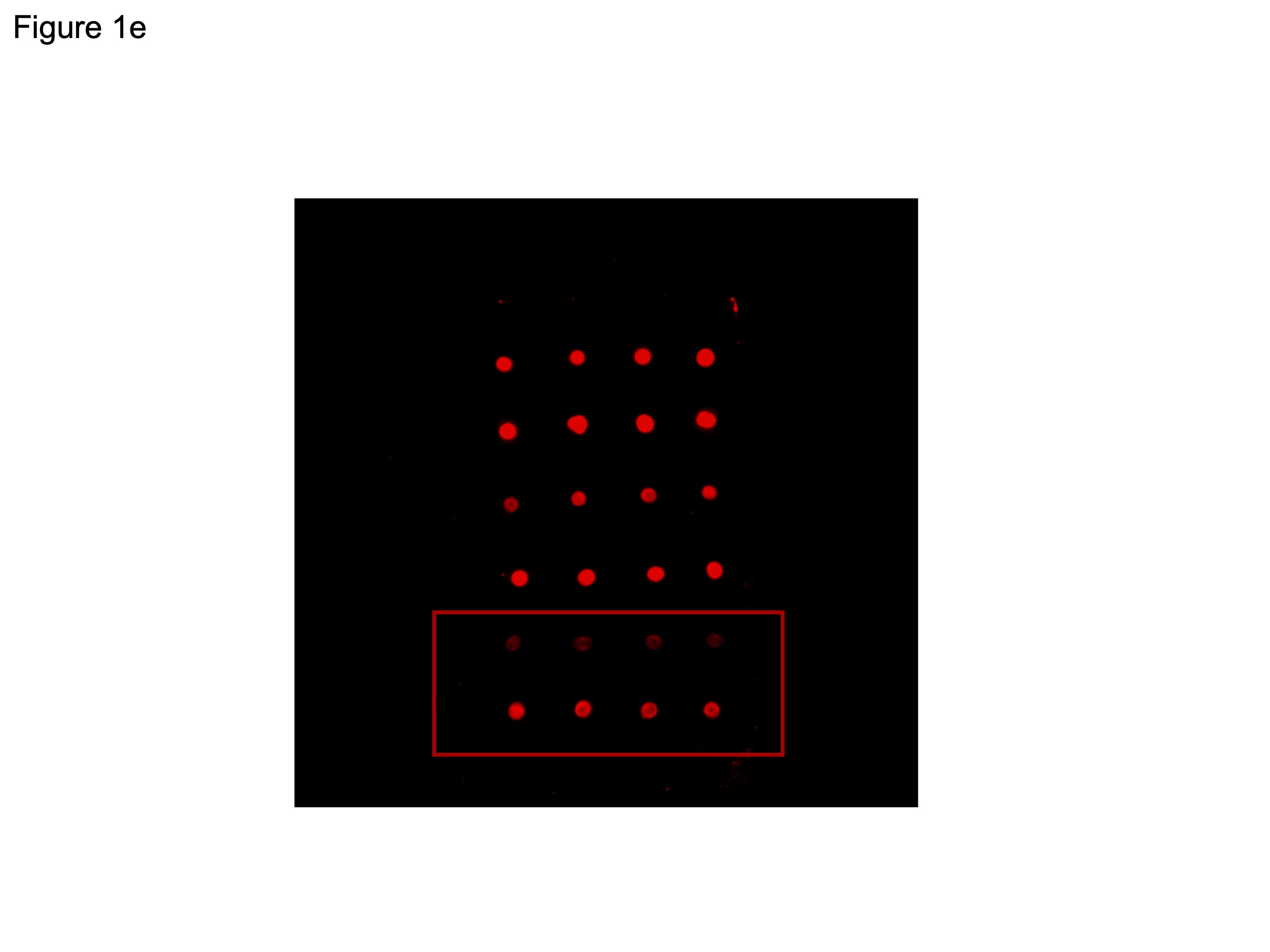

Supplement: Figure 1—source data 4. [file elife-79736-fig1-data4.zip › Figure 1-source data 4/Uncropped_Labeled_Gel_Figure 1e.jpg]

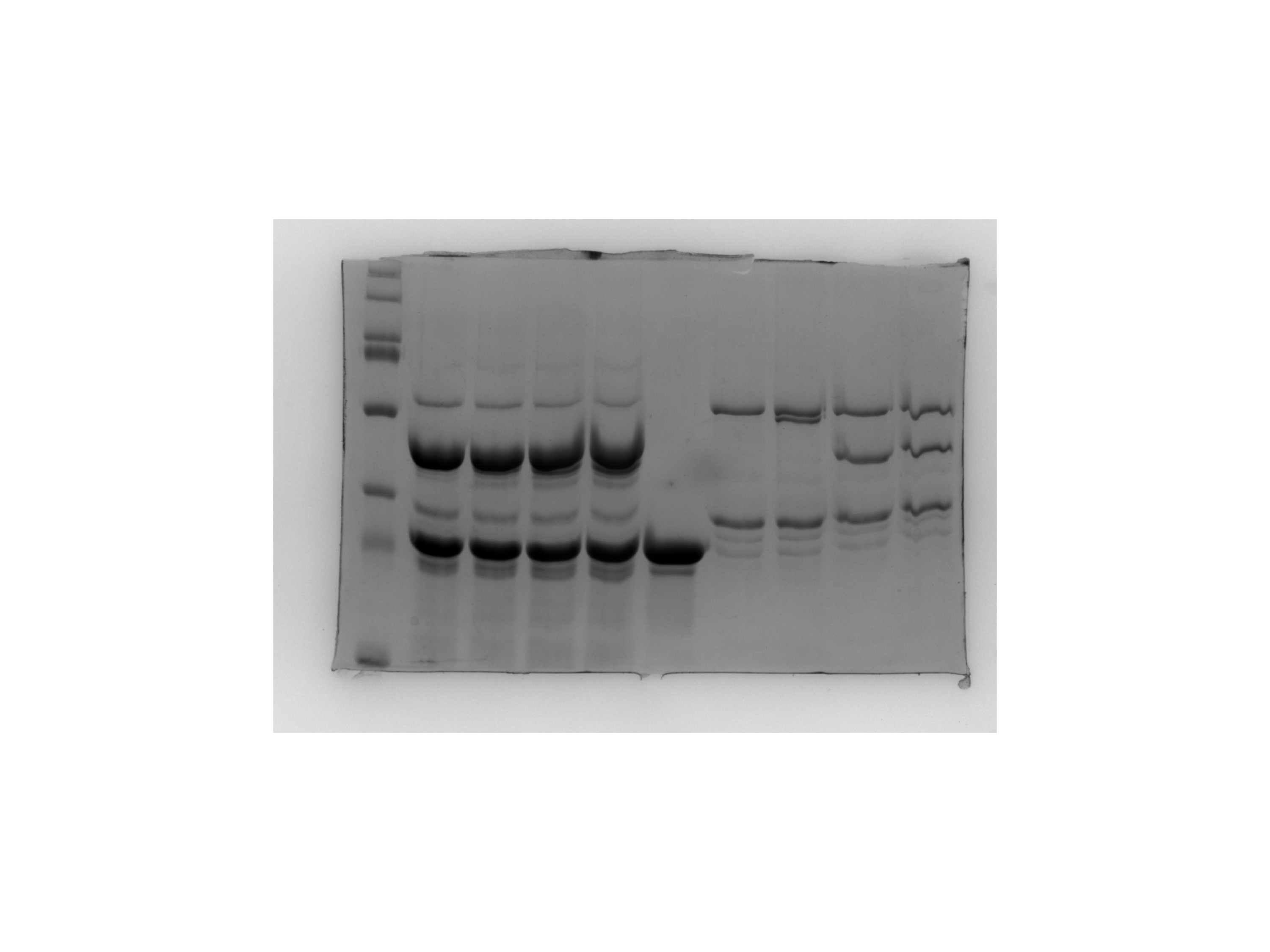

Supplement: Figure 1—source data 4. [file elife-79736-fig1-data4.zip › Figure 1-source data 4/Figure 1e_input.jpg]

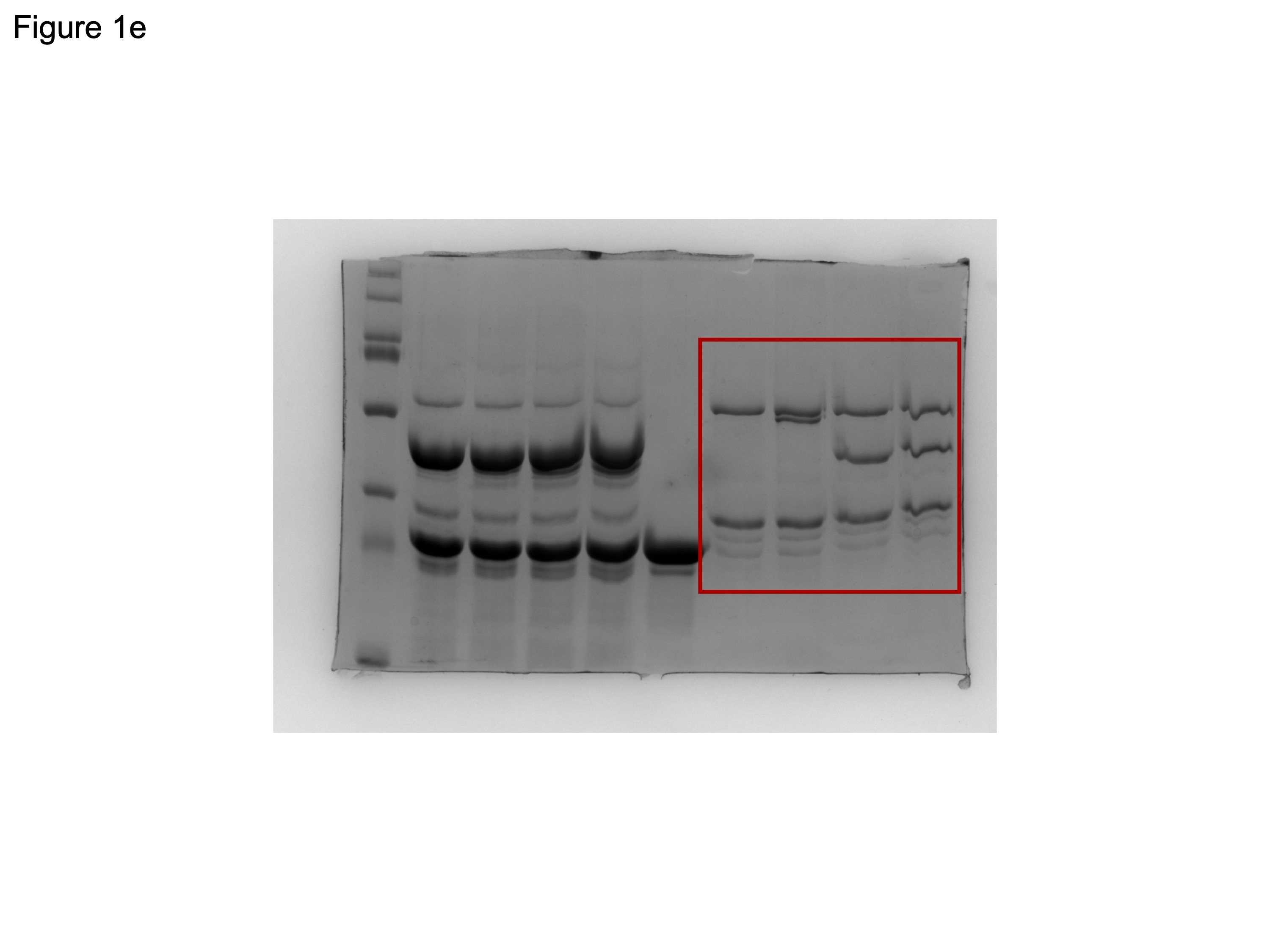

Supplement: Figure 1—source data 4. [file elife-79736-fig1-data4.zip › Figure 1-source data 4/Uncropped_Labeled_Gel_Figure 1e_input.jpg]

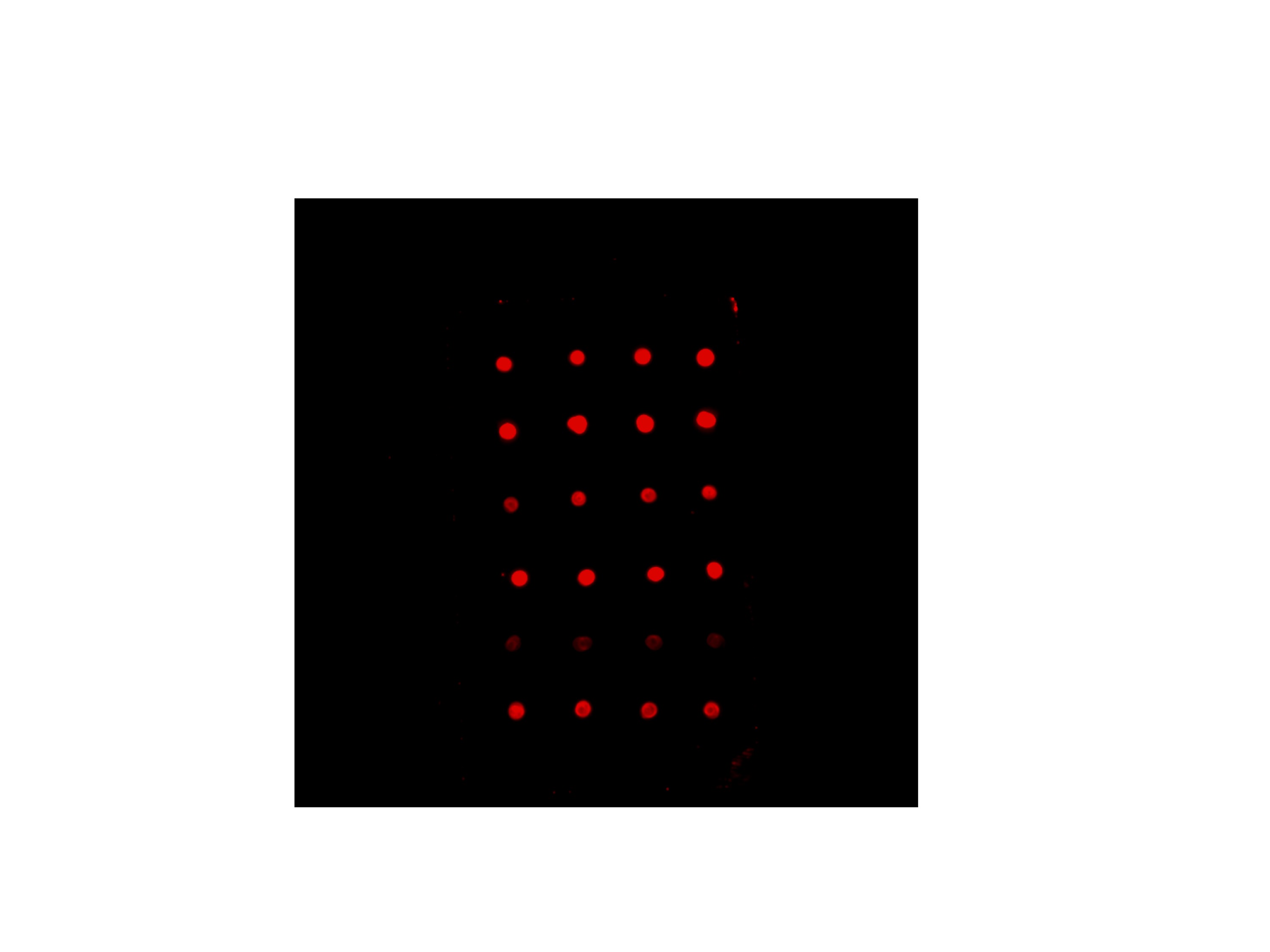

Supplement: Figure 1—source data 4. [file elife-79736-fig1-data4.zip › Figure 1-source data 4/Figure 1e.jpg]

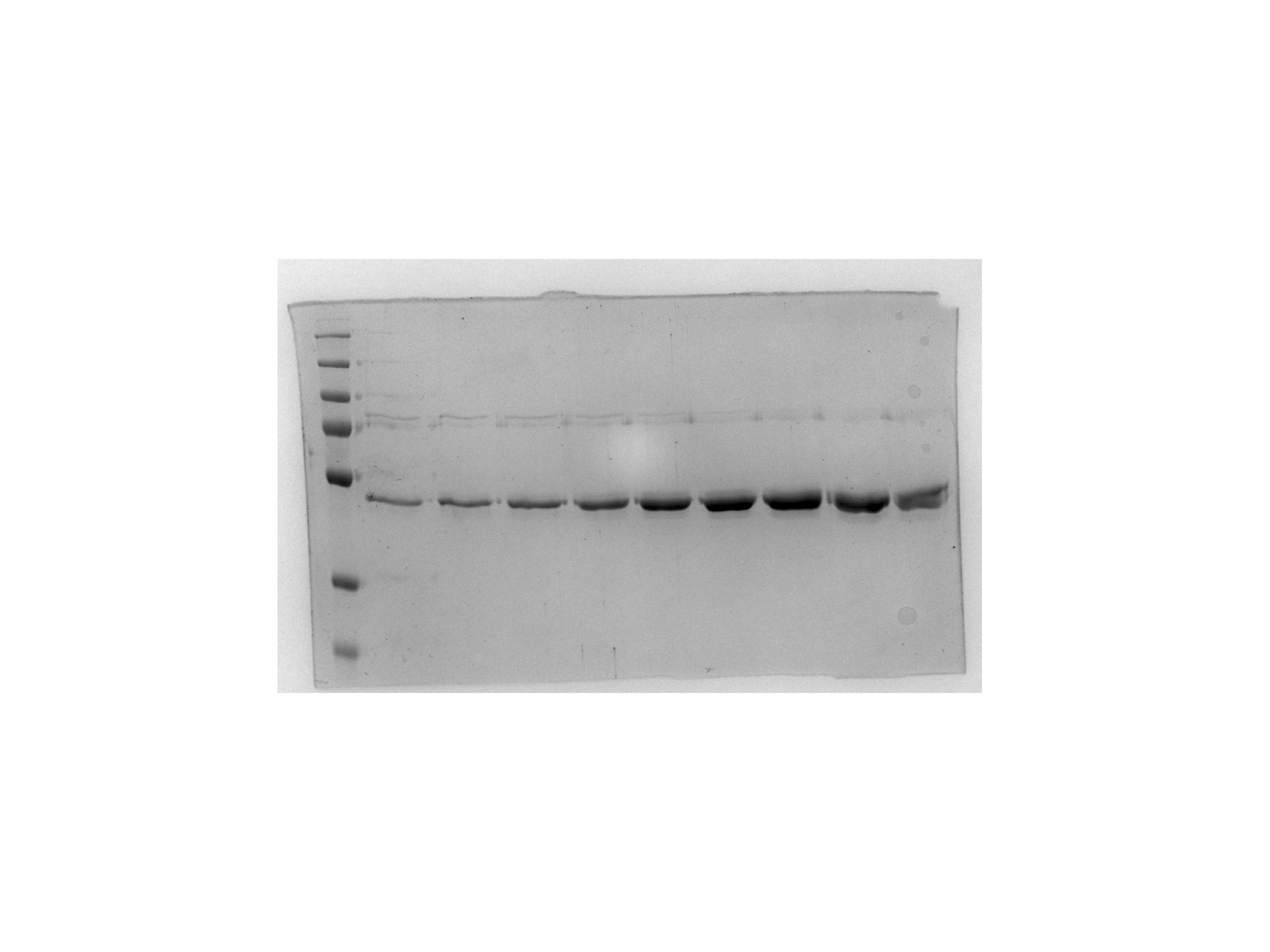

Supplement: Figure 1—figure supplement 1—source data 1. [file elife-79736-fig1-figsupp1-data1.zip › Figure 1-figure supplement 1-source data 1/Figure1-figure supplement 1b_right.jpg]

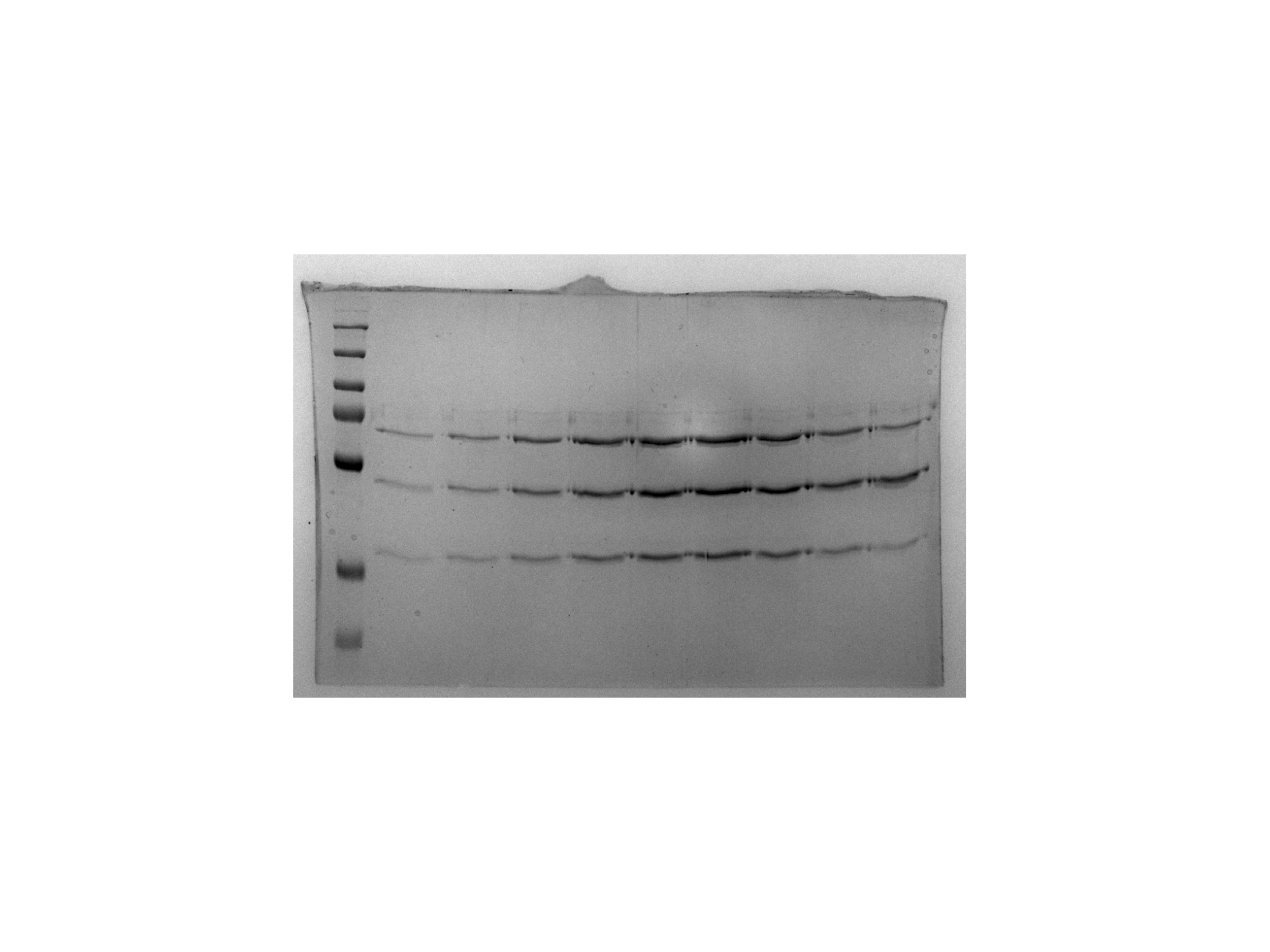

Supplement: Figure 1—figure supplement 1—source data 1. [file elife-79736-fig1-figsupp1-data1.zip › Figure 1-figure supplement 1-source data 1/Figure1-figure supplement 1b_middle.jpg]

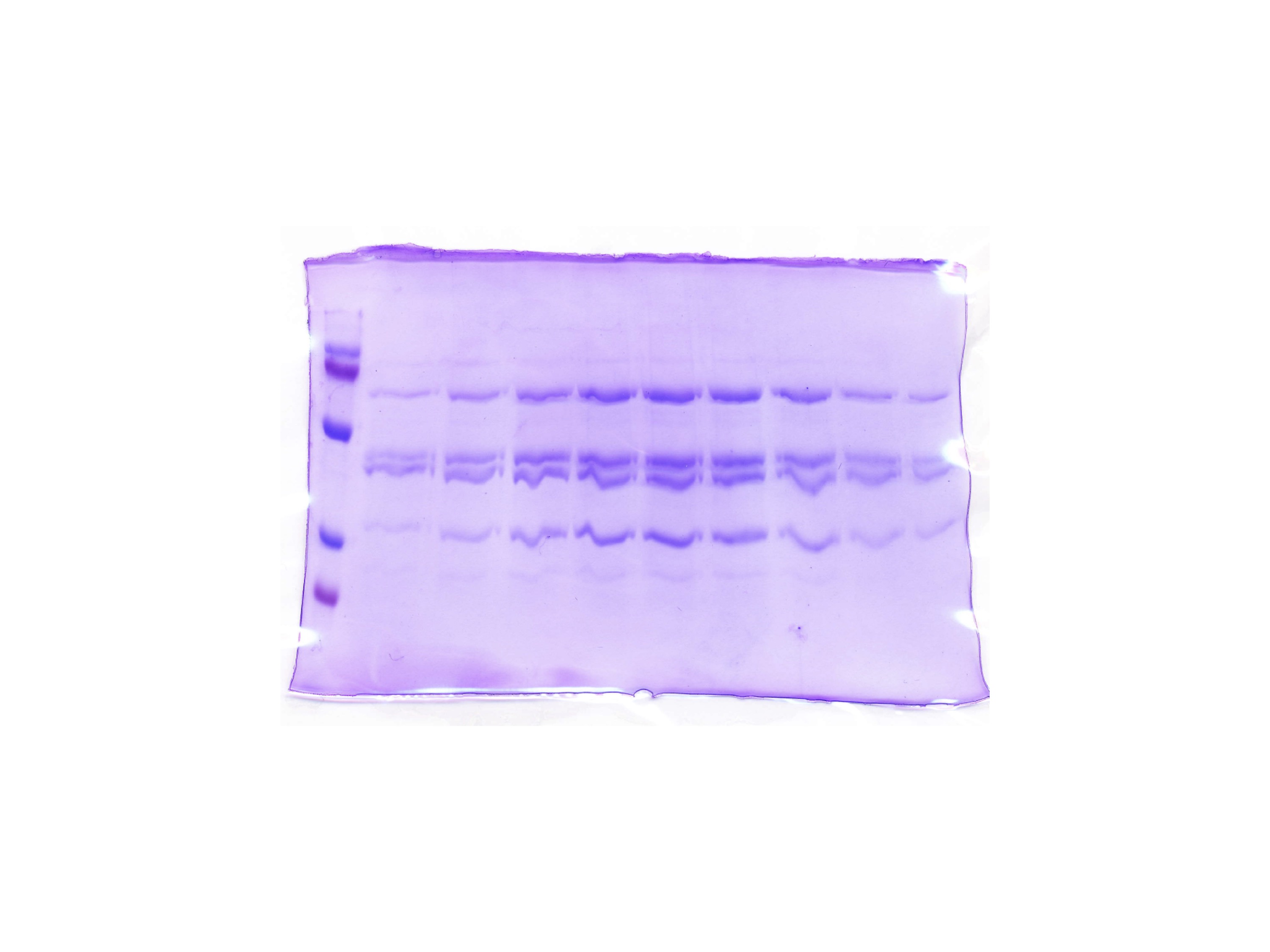

Supplement: Figure 1—figure supplement 1—source data 1. [file elife-79736-fig1-figsupp1-data1.zip › Figure 1-figure supplement 1-source data 1/Figure1-figure supplement 1b_left.jpg]

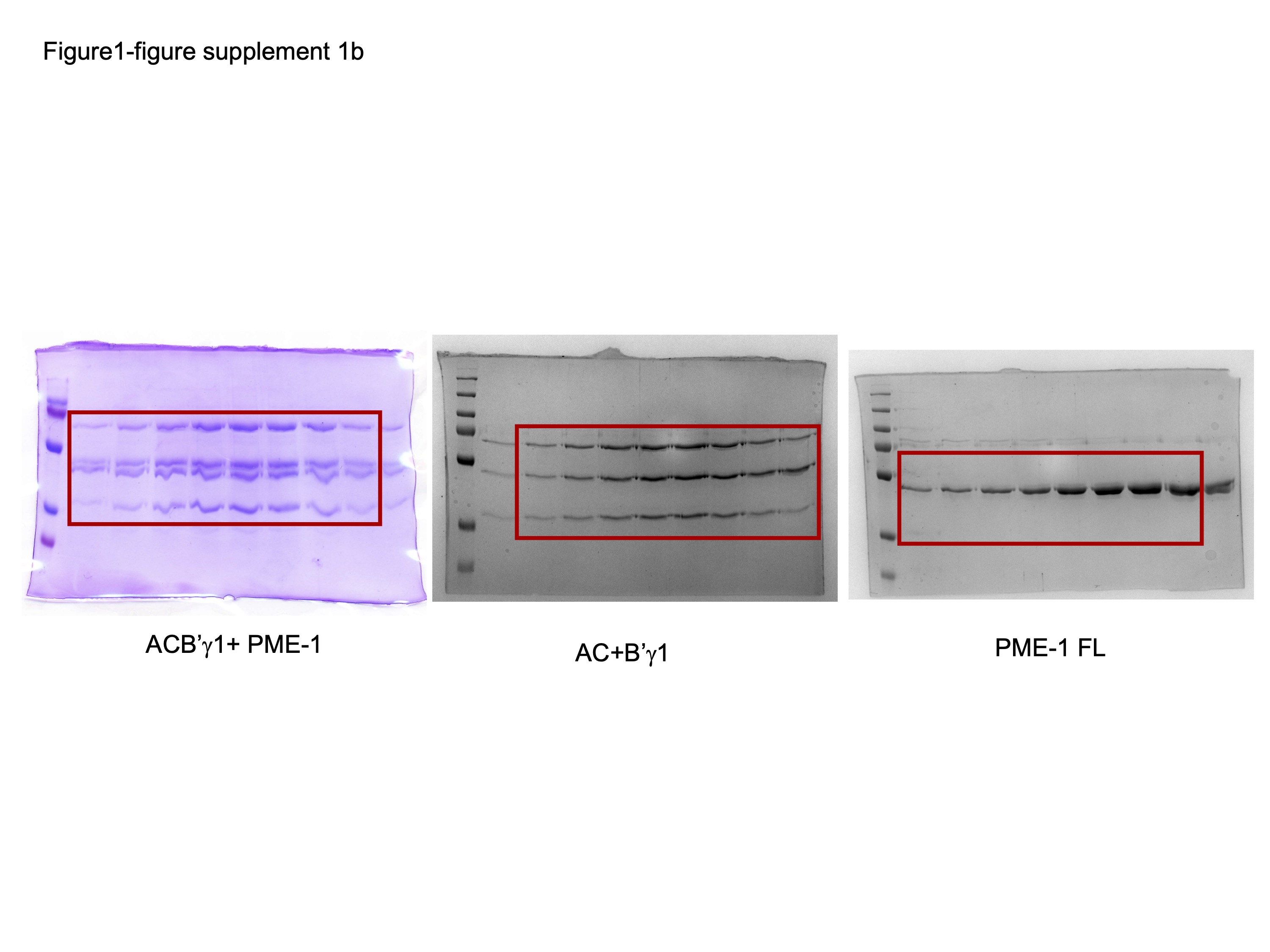

Supplement: Figure 1—figure supplement 1—source data 1. [file elife-79736-fig1-figsupp1-data1.zip › Figure 1-figure supplement 1-source data 1/Uncropped_Labeled_Gel_Figure1-figure supplement 1b.jpg]

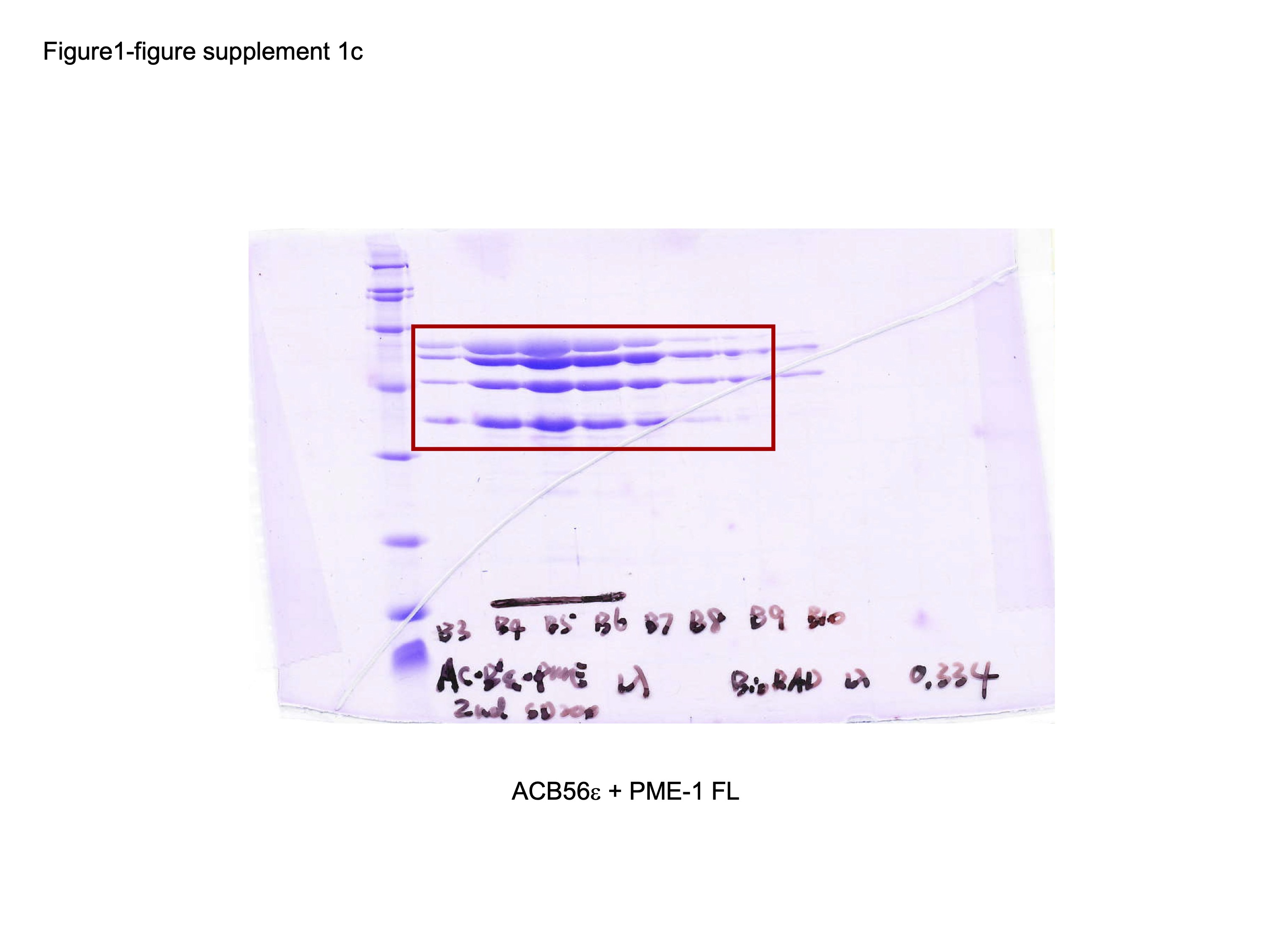

Supplement: Figure 1—figure supplement 1—source data 2. [file elife-79736-fig1-figsupp1-data2.zip › Figure 1-figure supplement 1-source data 2/Uncropped_Labeled_Gel_Figure 1-figure supplement 1c.jpg]

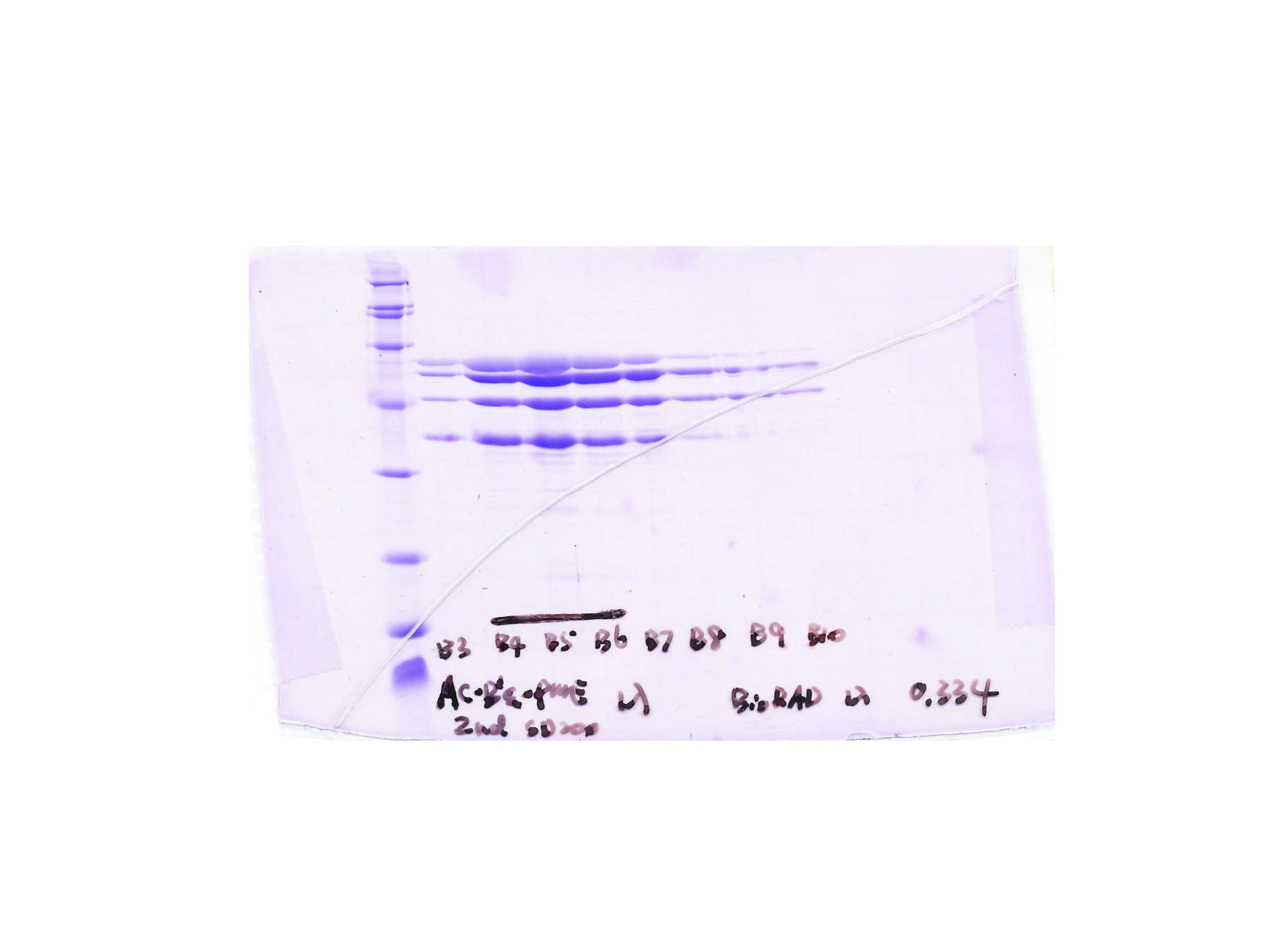

Supplement: Figure 1—figure supplement 1—source data 2. [file elife-79736-fig1-figsupp1-data2.zip › Figure 1-figure supplement 1-source data 2/Figure 1-figure supplement 1c.jpg]

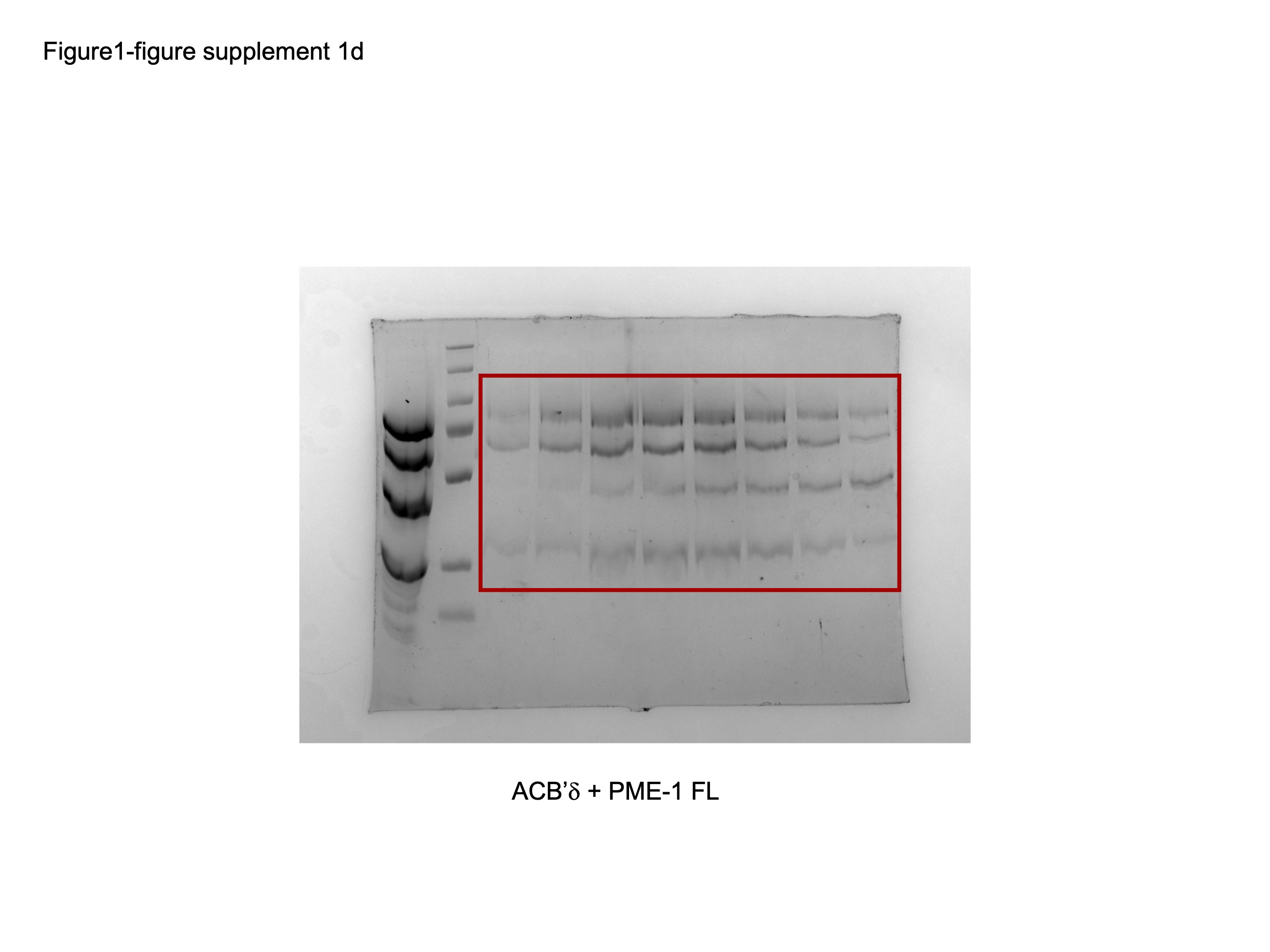

Supplement: Figure 1—figure supplement 1—source data 3. [file elife-79736-fig1-figsupp1-data3.zip › Figure 1-figure supplement 1-source data 3/Uncropped_Labeled_Gel_Figure 1-figure supplement 1d.jpg]

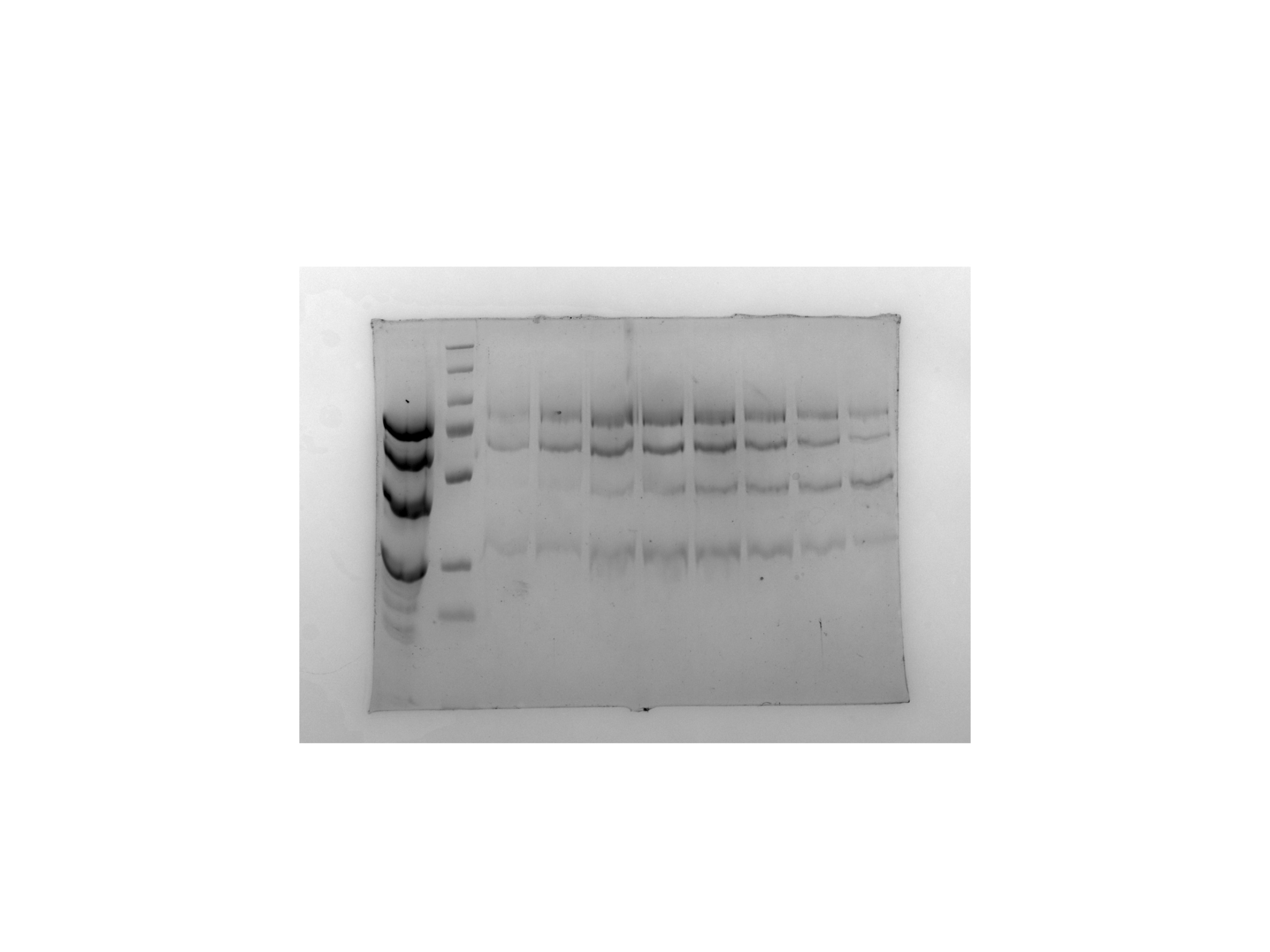

Supplement: Figure 1—figure supplement 1—source data 3. [file elife-79736-fig1-figsupp1-data3.zip › Figure 1-figure supplement 1-source data 3/Figure 1-figure supplement 1d.jpg]

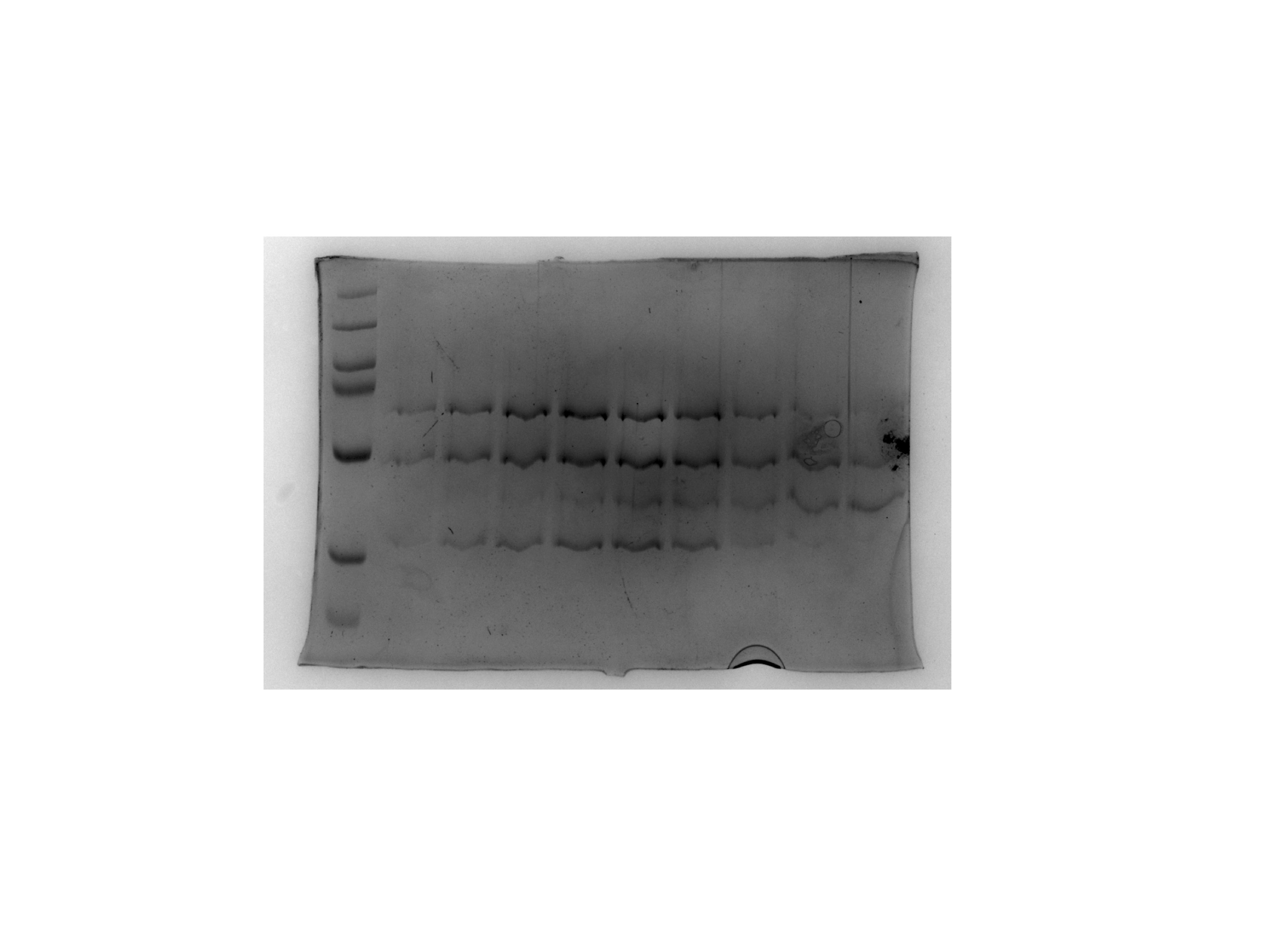

Supplement: Figure 2—source data 1. [file elife-79736-fig2-data1.zip › Figure 2-source data 1/Figure 2b_middle.jpg]

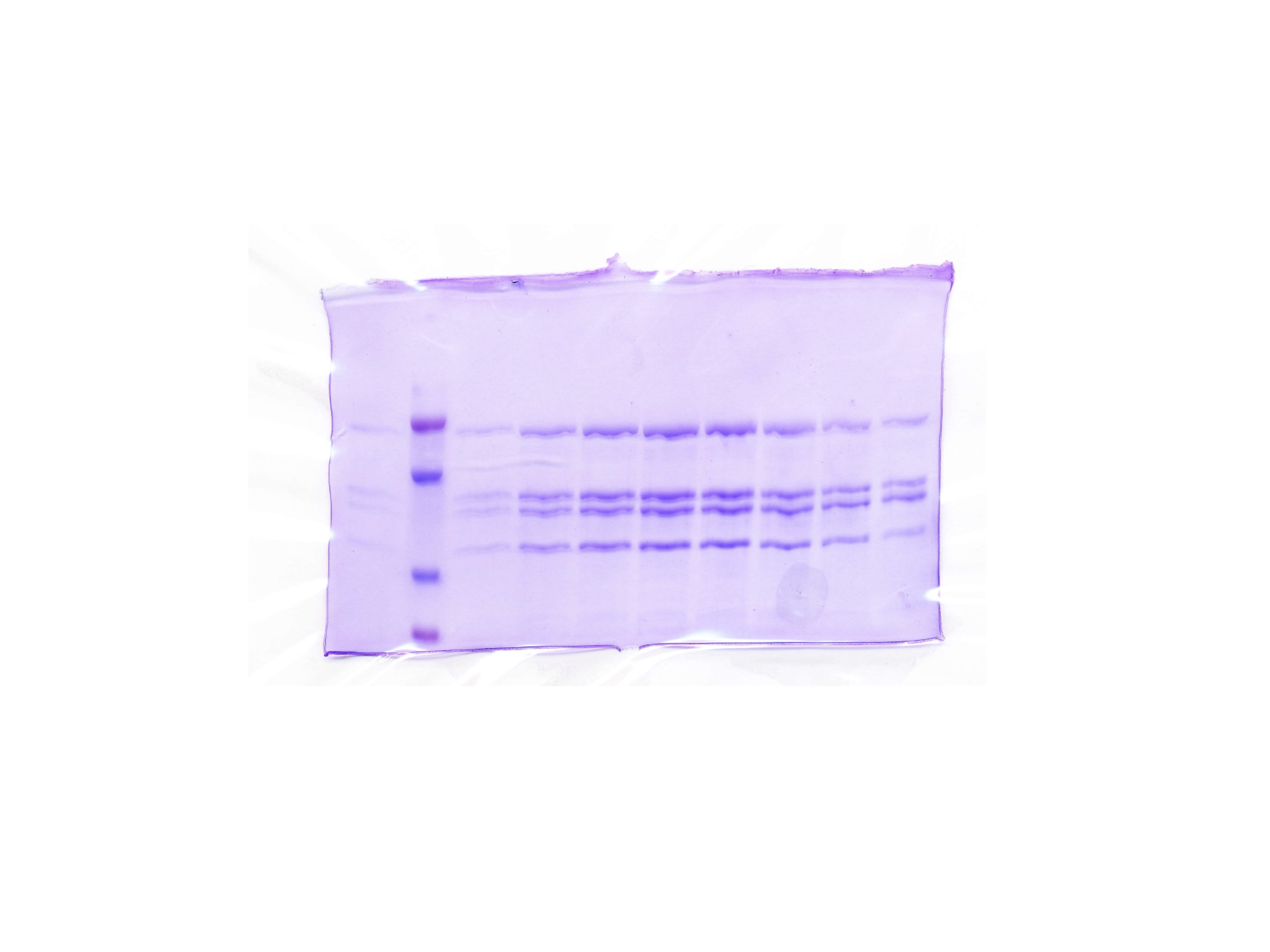

Supplement: Figure 2—source data 1. [file elife-79736-fig2-data1.zip › Figure 2-source data 1/Figure 2b_left.jpg]

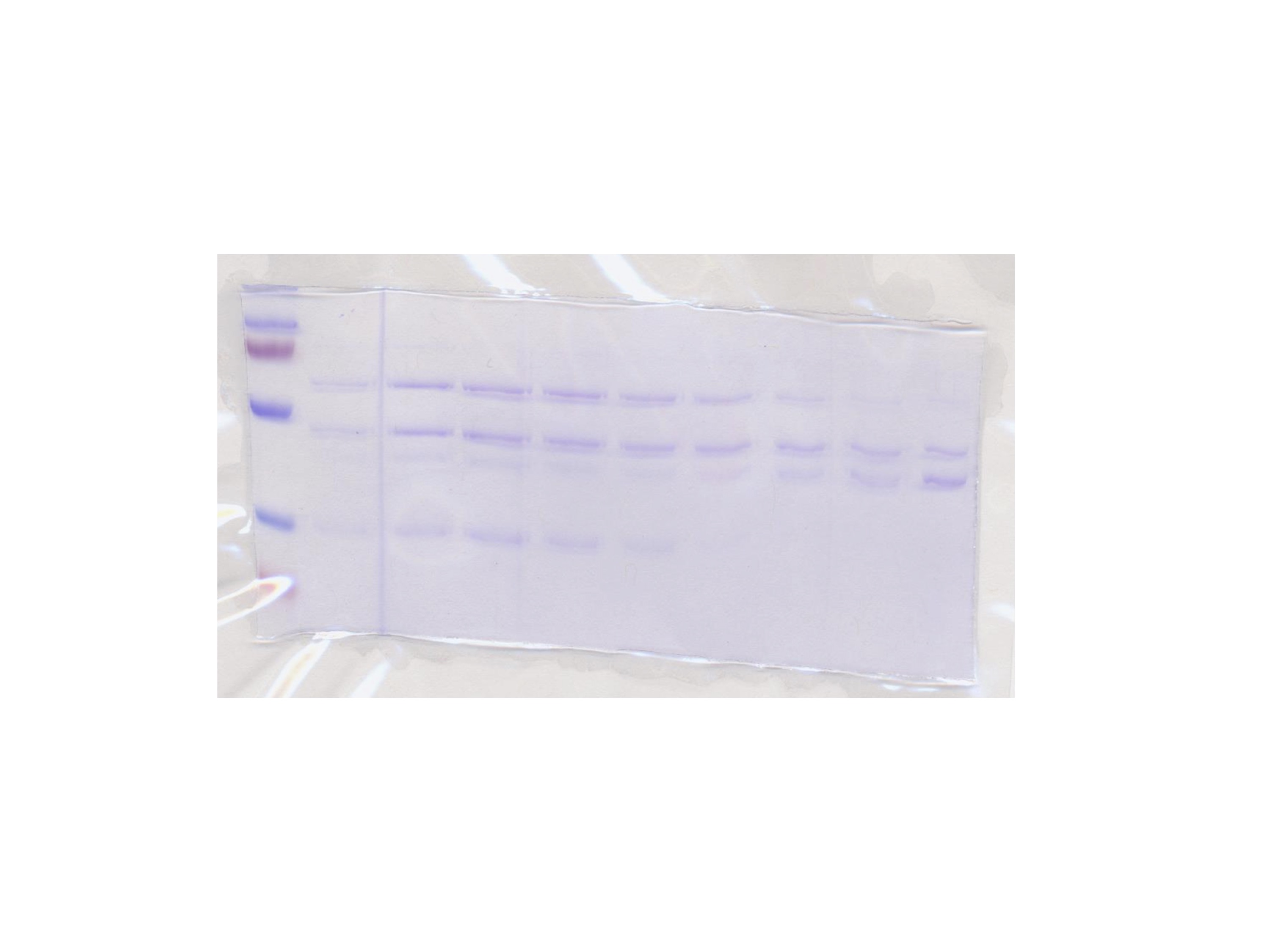

Supplement: Figure 2—source data 1. [file elife-79736-fig2-data1.zip › Figure 2-source data 1/Figure 2b_right.jpg]

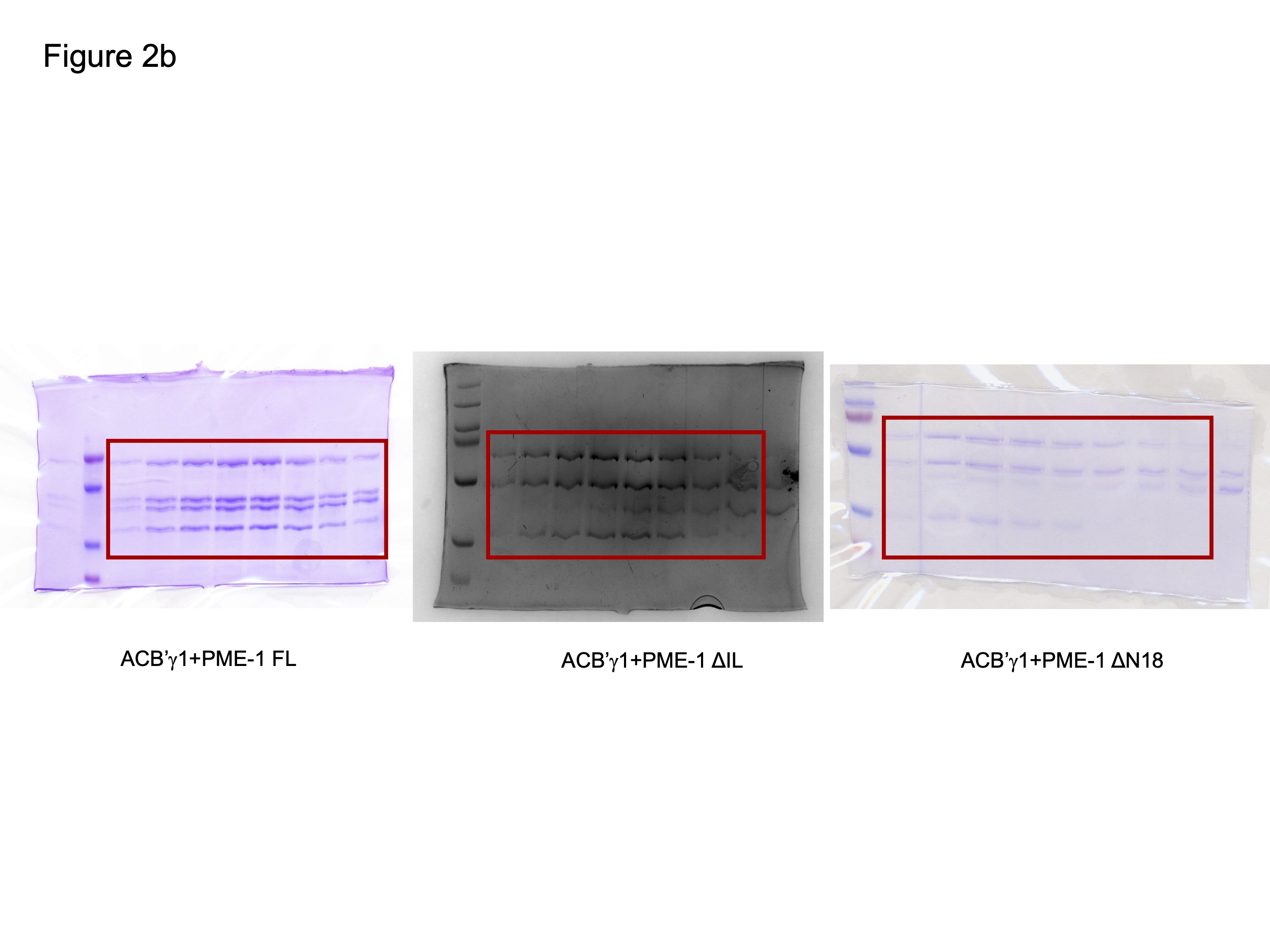

Supplement: Figure 2—source data 1. [file elife-79736-fig2-data1.zip › Figure 2-source data 1/Uncropped_Labeled_Gel_Figure 2b.jpg]

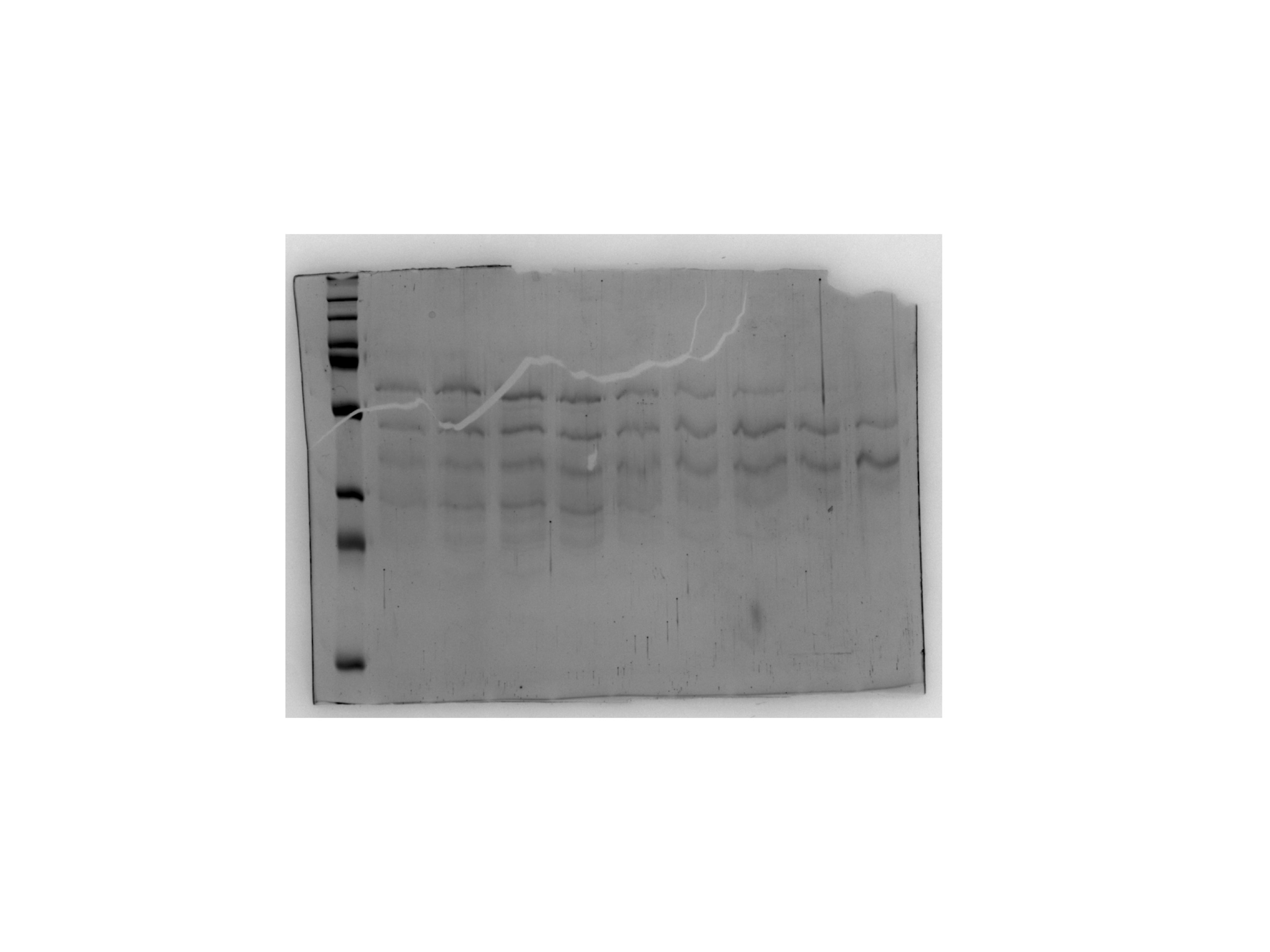

Supplement: Figure 2—source data 2. [file elife-79736-fig2-data2.zip › Figure 2-source data 2/Figure 2c_middle.jpg]

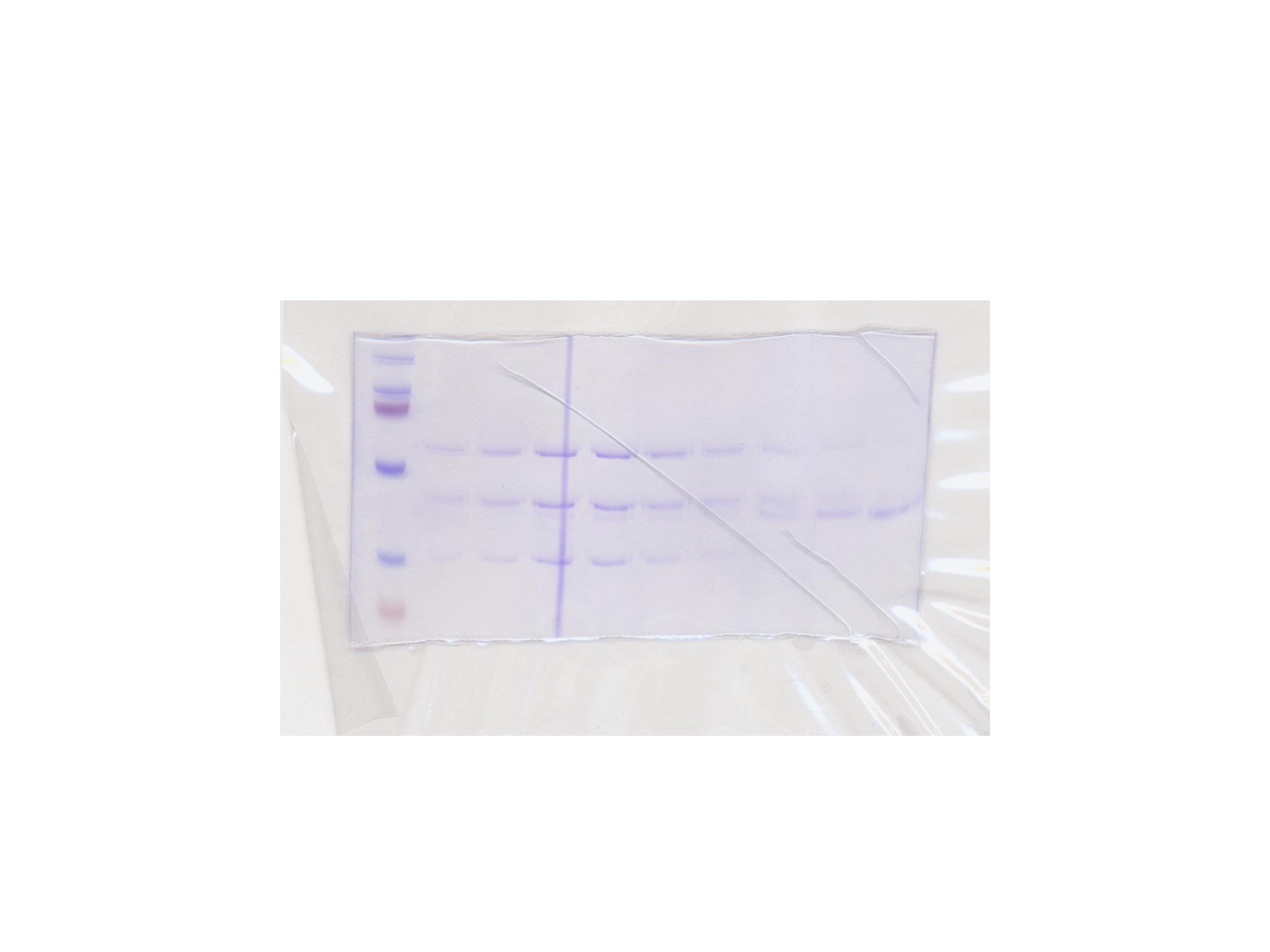

Supplement: Figure 2—source data 2. [file elife-79736-fig2-data2.zip › Figure 2-source data 2/Figure 2c_right.jpg]

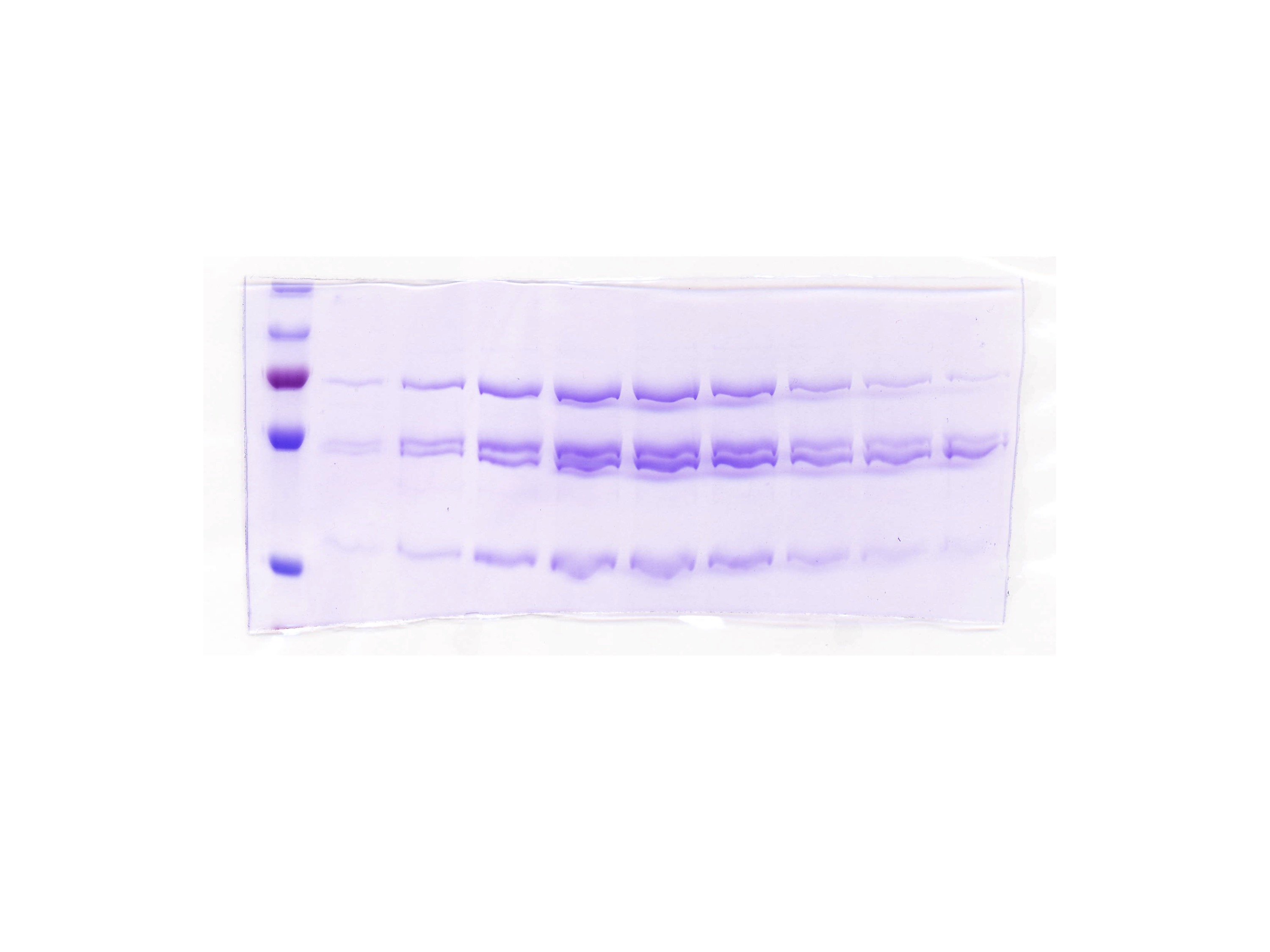

Supplement: Figure 2—source data 2. [file elife-79736-fig2-data2.zip › Figure 2-source data 2/Figure 2c_left.jpg]

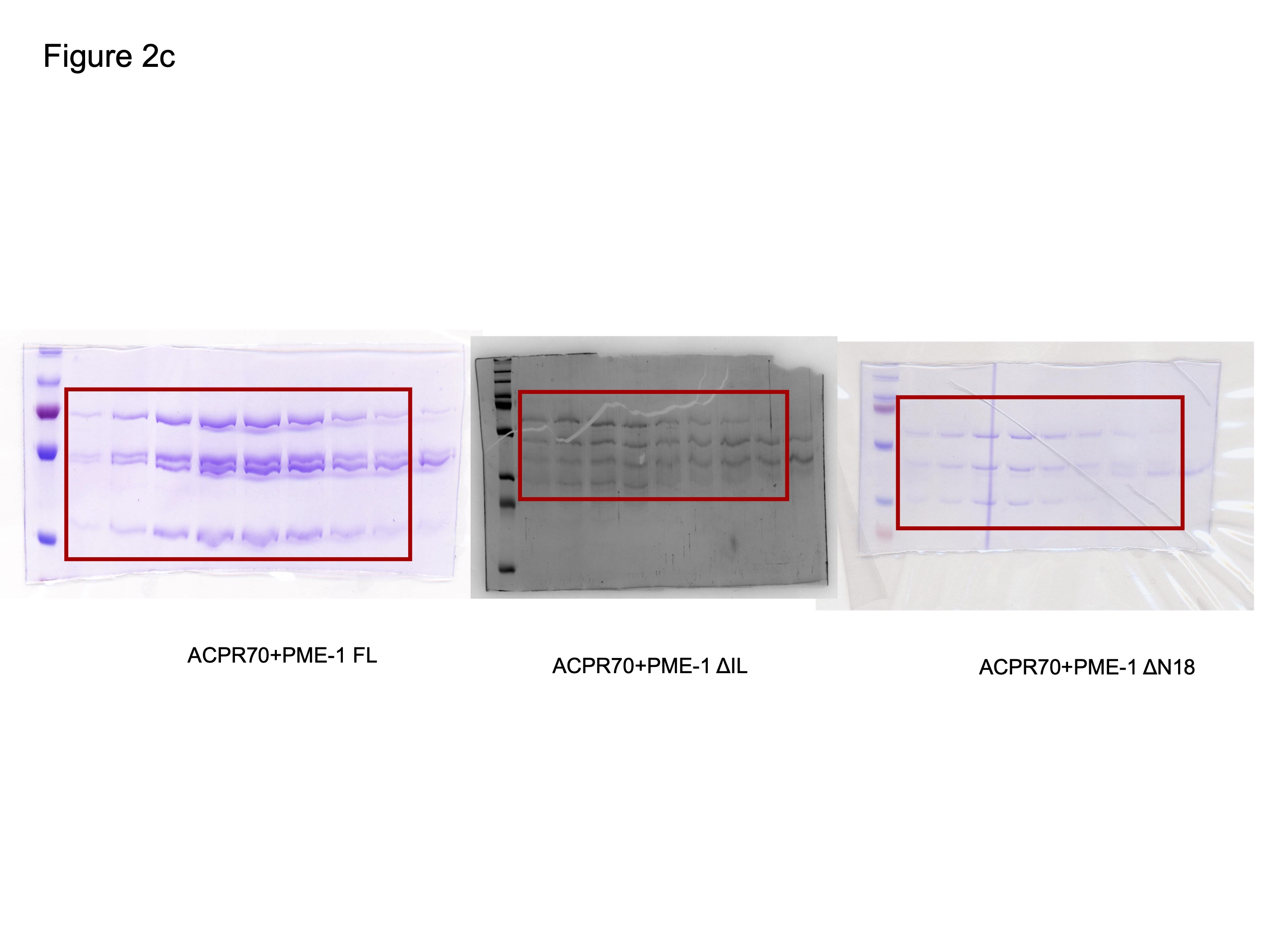

Supplement: Figure 2—source data 2. [file elife-79736-fig2-data2.zip › Figure 2-source data 2/Uncropped_Labeled_Gel_Figure 2c.jpg]

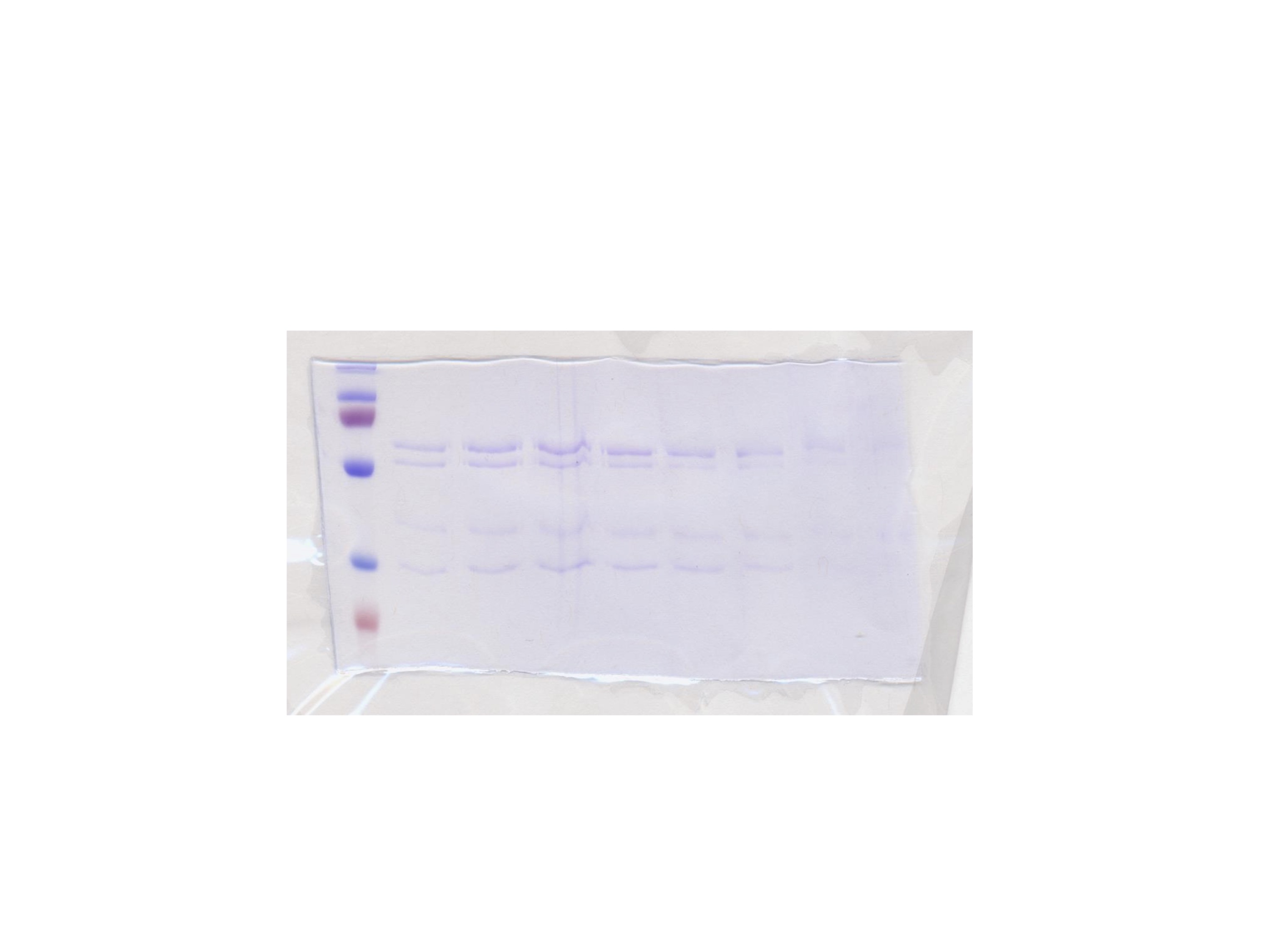

Supplement: Figure 2—source data 3. [file elife-79736-fig2-data3.zip › Figure 2-source data 3/Figure 2d_middle.jpg]

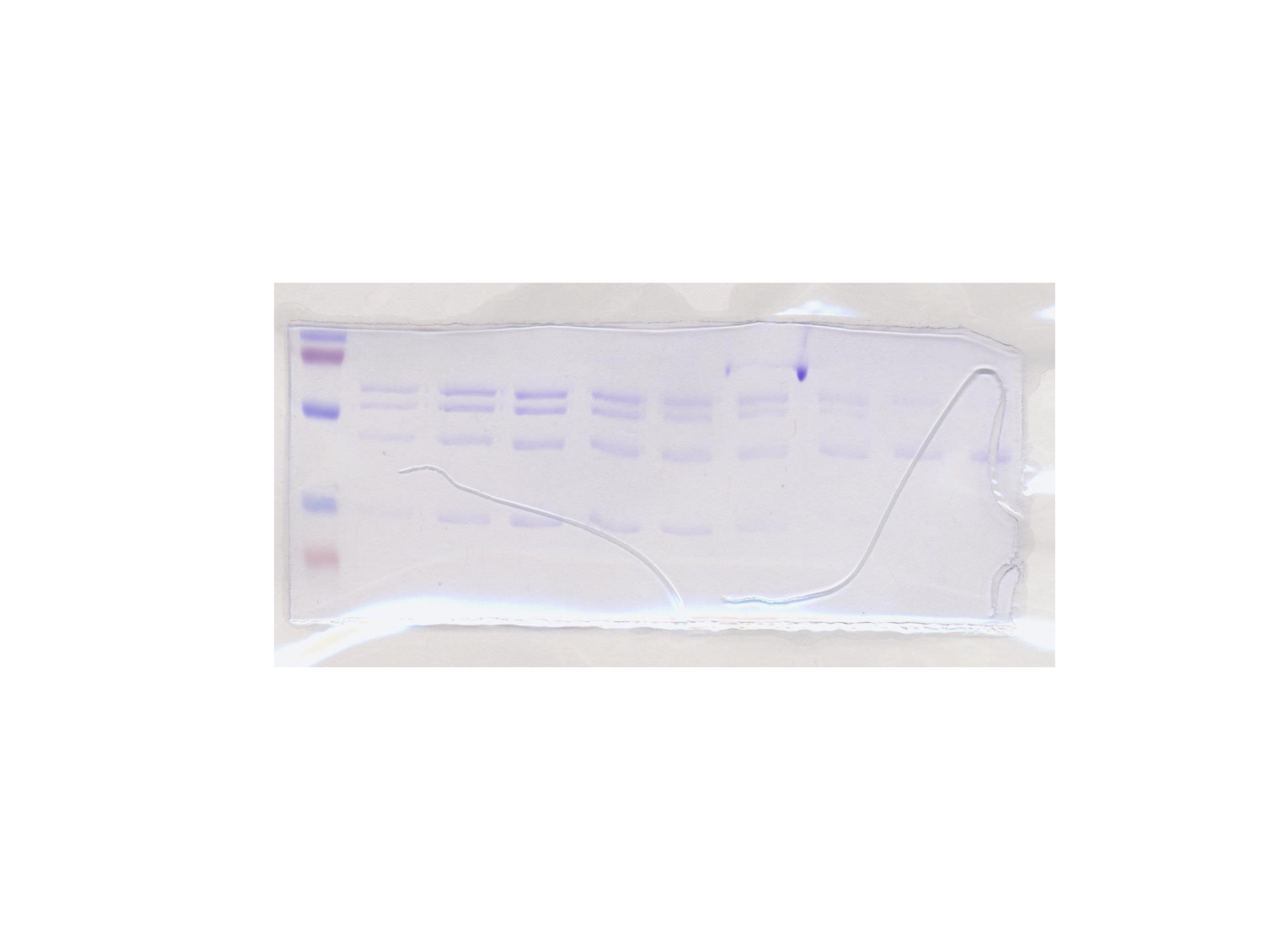

Supplement: Figure 2—source data 3. [file elife-79736-fig2-data3.zip › Figure 2-source data 3/Figure 2d_left.jpg]

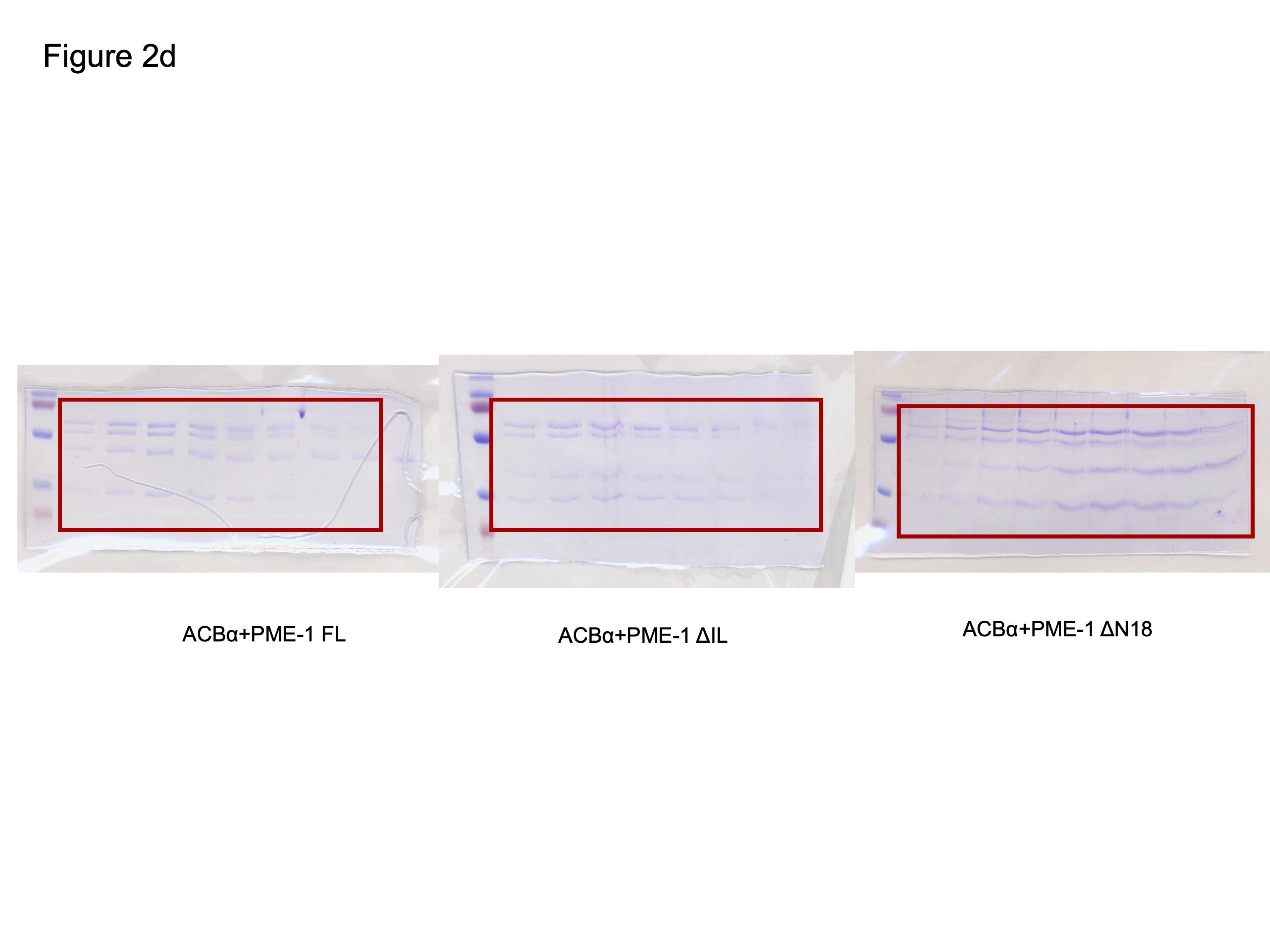

Supplement: Figure 2—source data 3. [file elife-79736-fig2-data3.zip › Figure 2-source data 3/Uncropped_Labeled_Gel_Figure 2d.jpg]

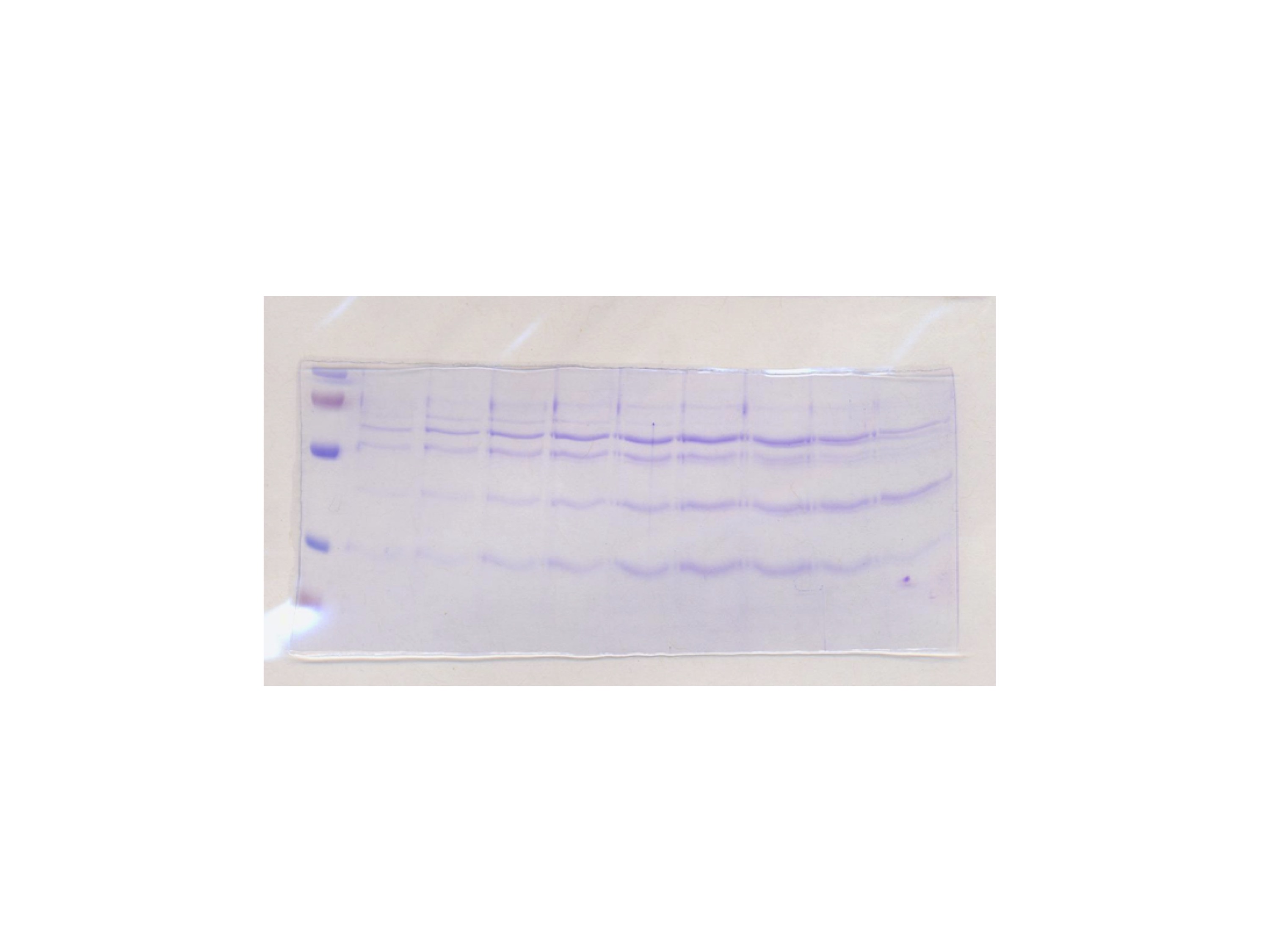

Supplement: Figure 2—source data 3. [file elife-79736-fig2-data3.zip › Figure 2-source data 3/Figure 2d_right.jpg]

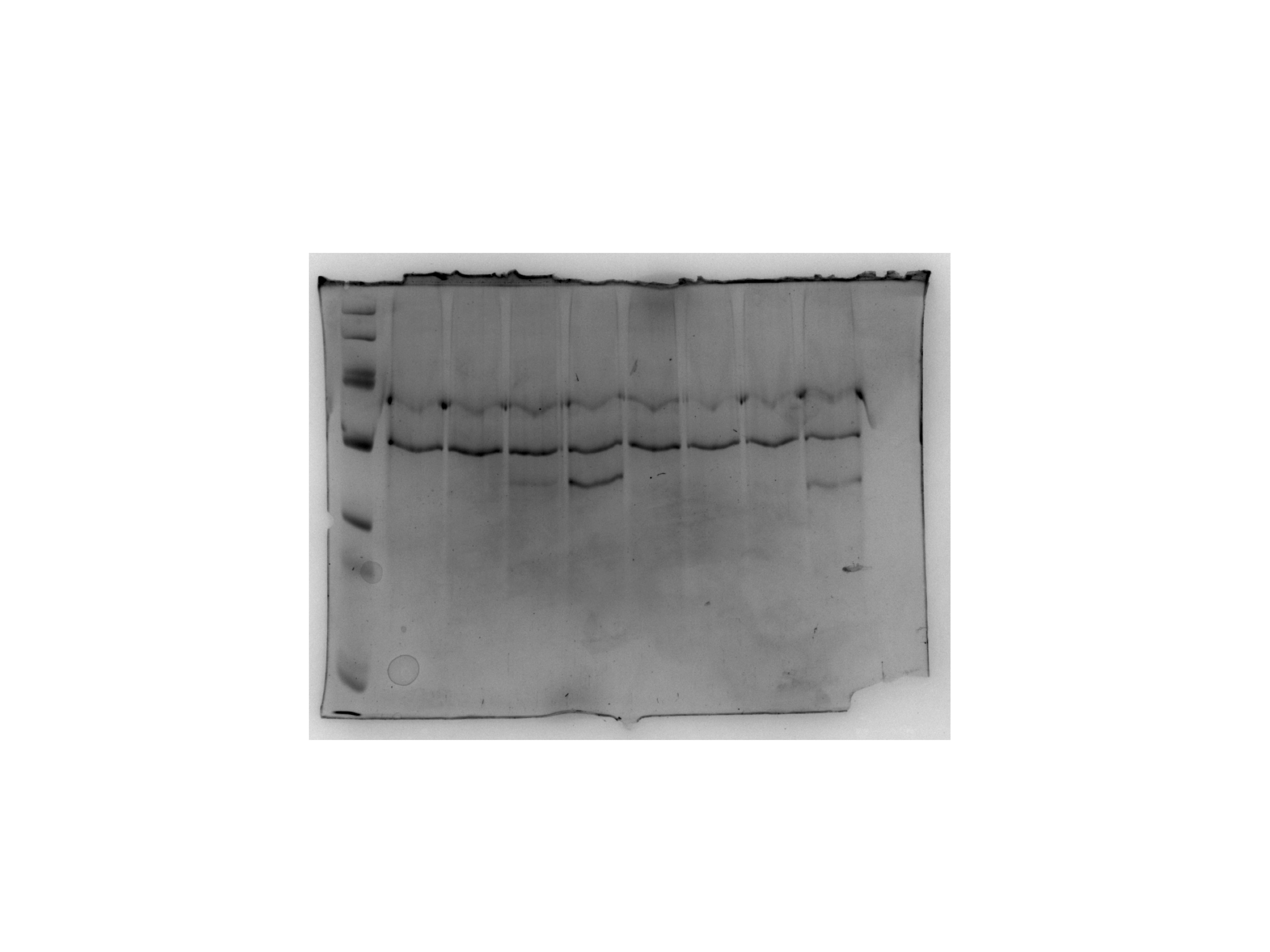

Supplement: Figure 2—figure supplement 1—source data 1. [file elife-79736-fig2-figsupp1-data1.zip › Figure 2-figure supplement 1-source data 1/Figure2-figure supplement 1c_PME-1 FL and ╬öIL.jpg]

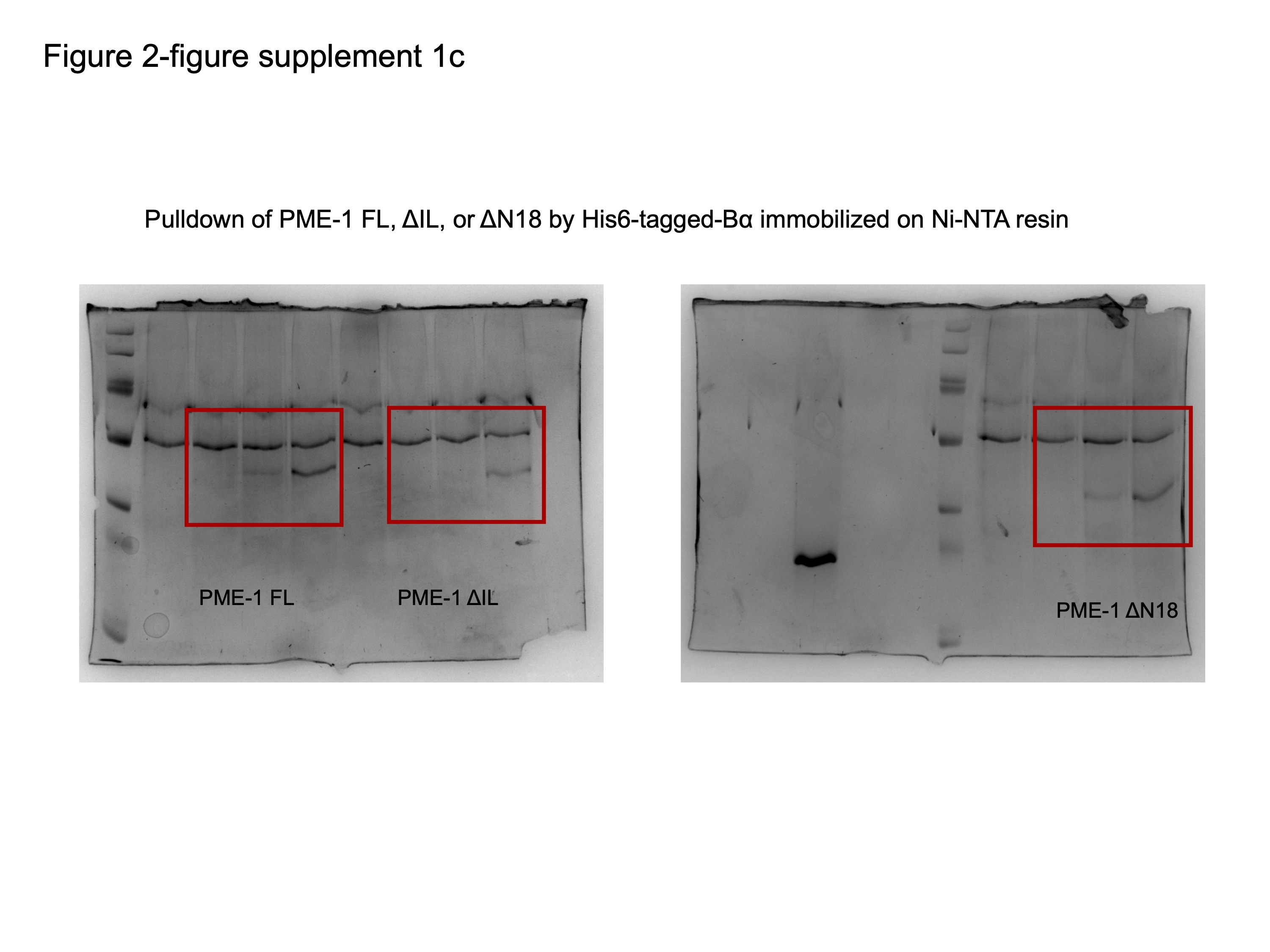

Supplement: Figure 2—figure supplement 1—source data 1. [file elife-79736-fig2-figsupp1-data1.zip › Figure 2-figure supplement 1-source data 1/Uncropped_Labeled_Gel_Figure 2-figure supplement 1c.jpg]

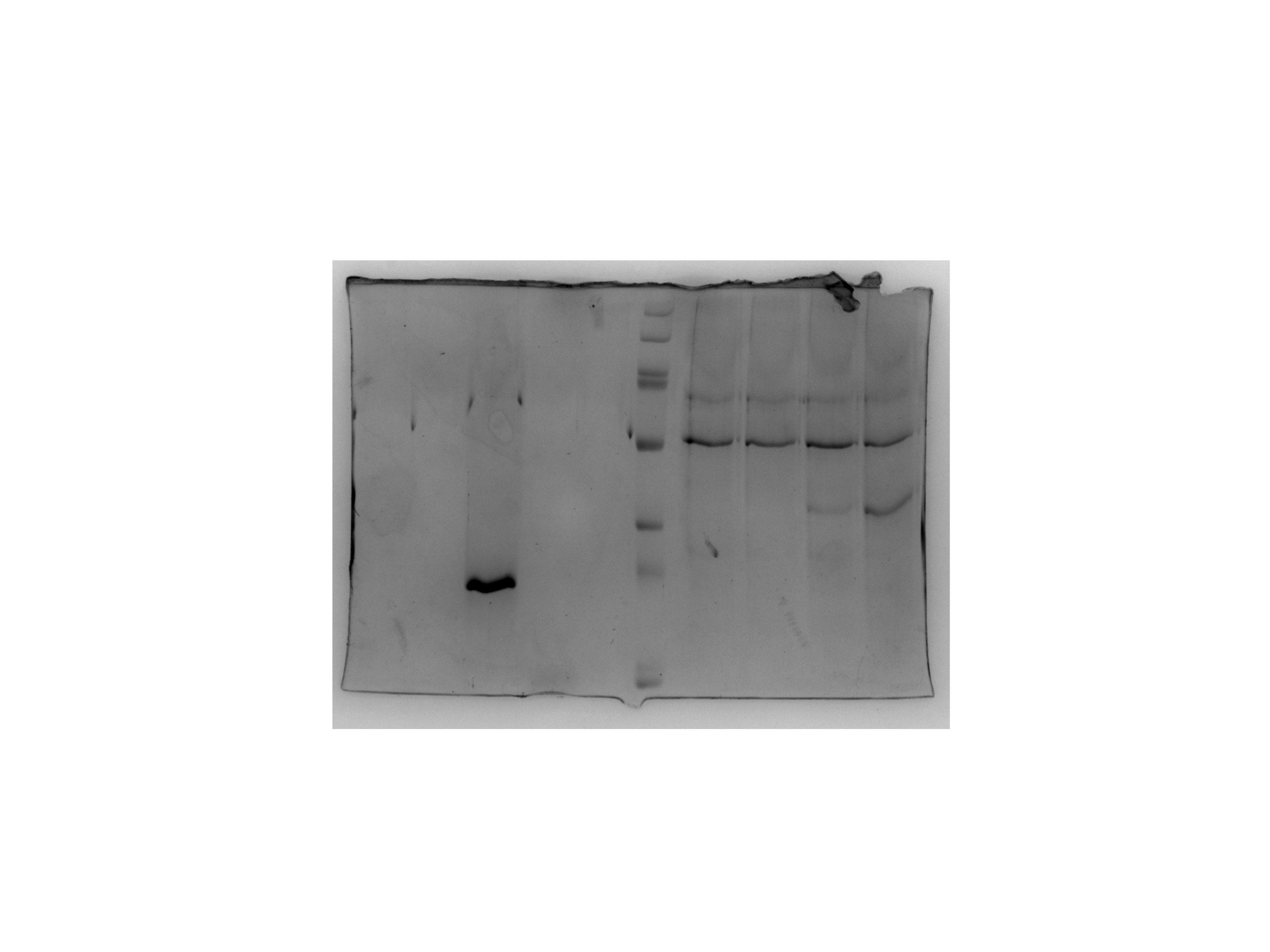

Supplement: Figure 2—figure supplement 1—source data 1. [file elife-79736-fig2-figsupp1-data1.zip › Figure 2-figure supplement 1-source data 1/Figure2-figure supplement 1c_PME-1 ╬öN18.jpg]

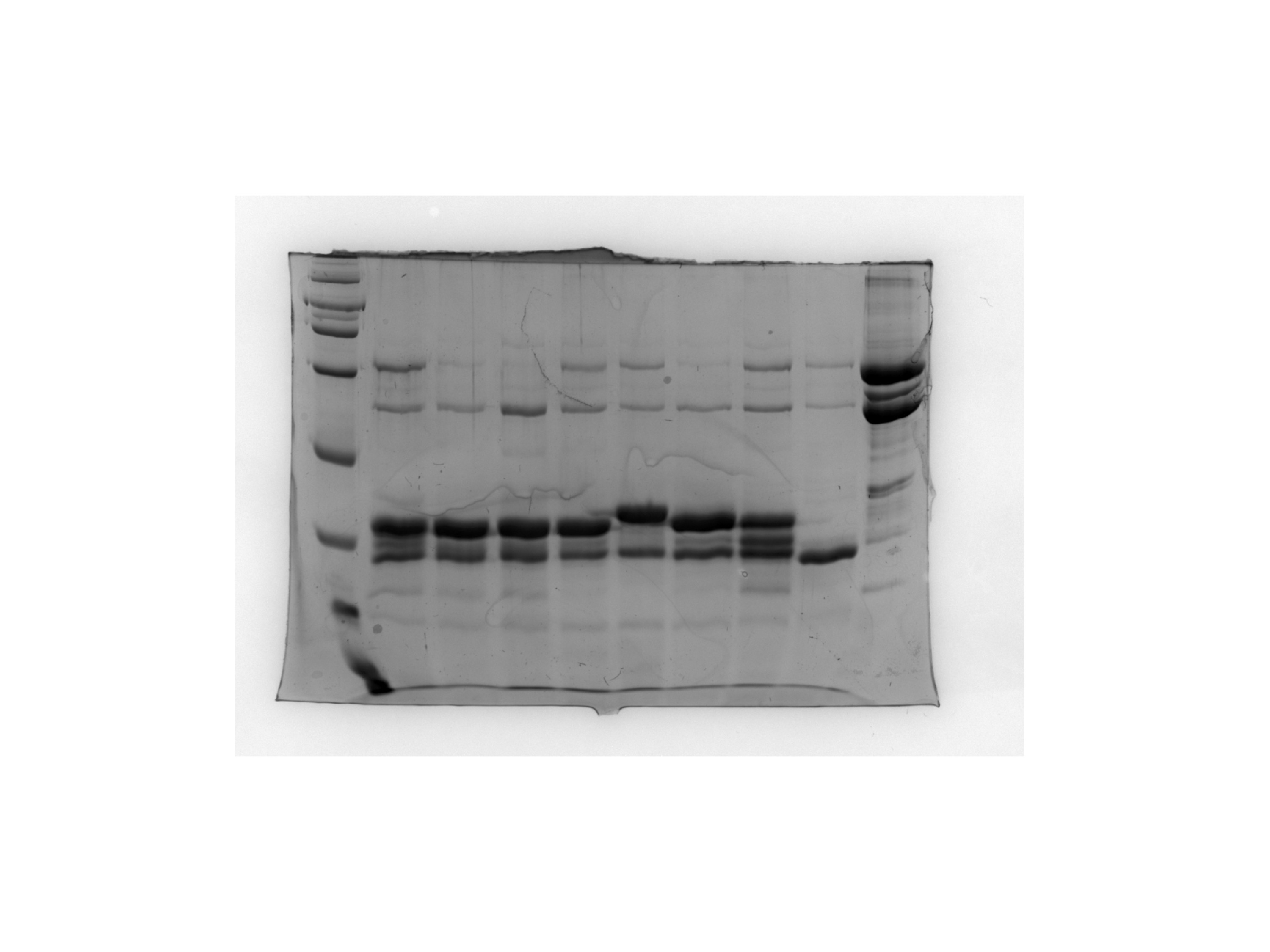

Supplement: Figure 2—figure supplement 2—source data 1. [file elife-79736-fig2-figsupp2-data1.zip › Figure 2-figure supplement 2-source data 1/Figure 2-figure supplement 2b_1.jpg]

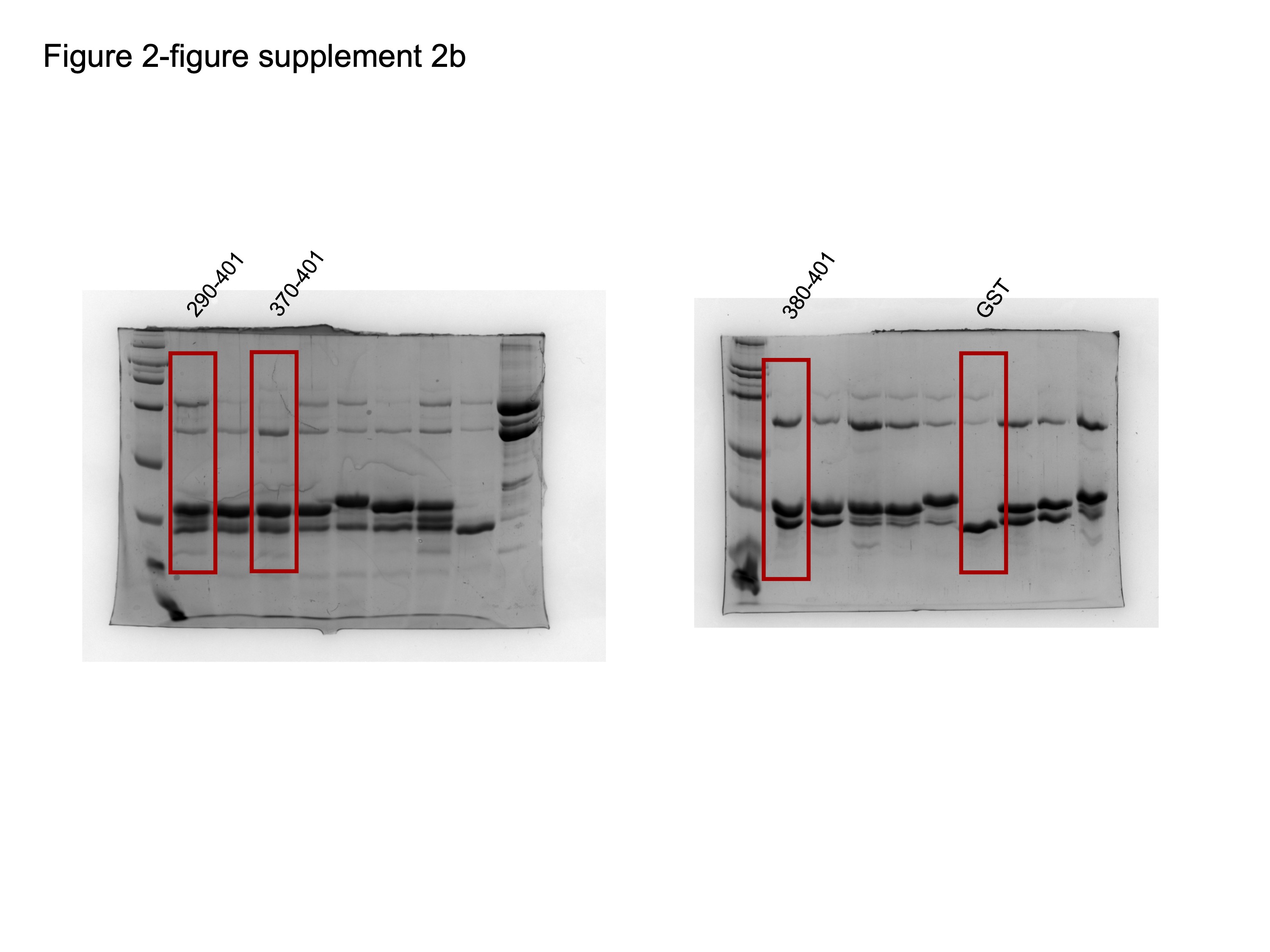

Supplement: Figure 2—figure supplement 2—source data 1. [file elife-79736-fig2-figsupp2-data1.zip › Figure 2-figure supplement 2-source data 1/Uncropped_Labeled_Gels_Figure 2-figure supplement 2b.jpg]

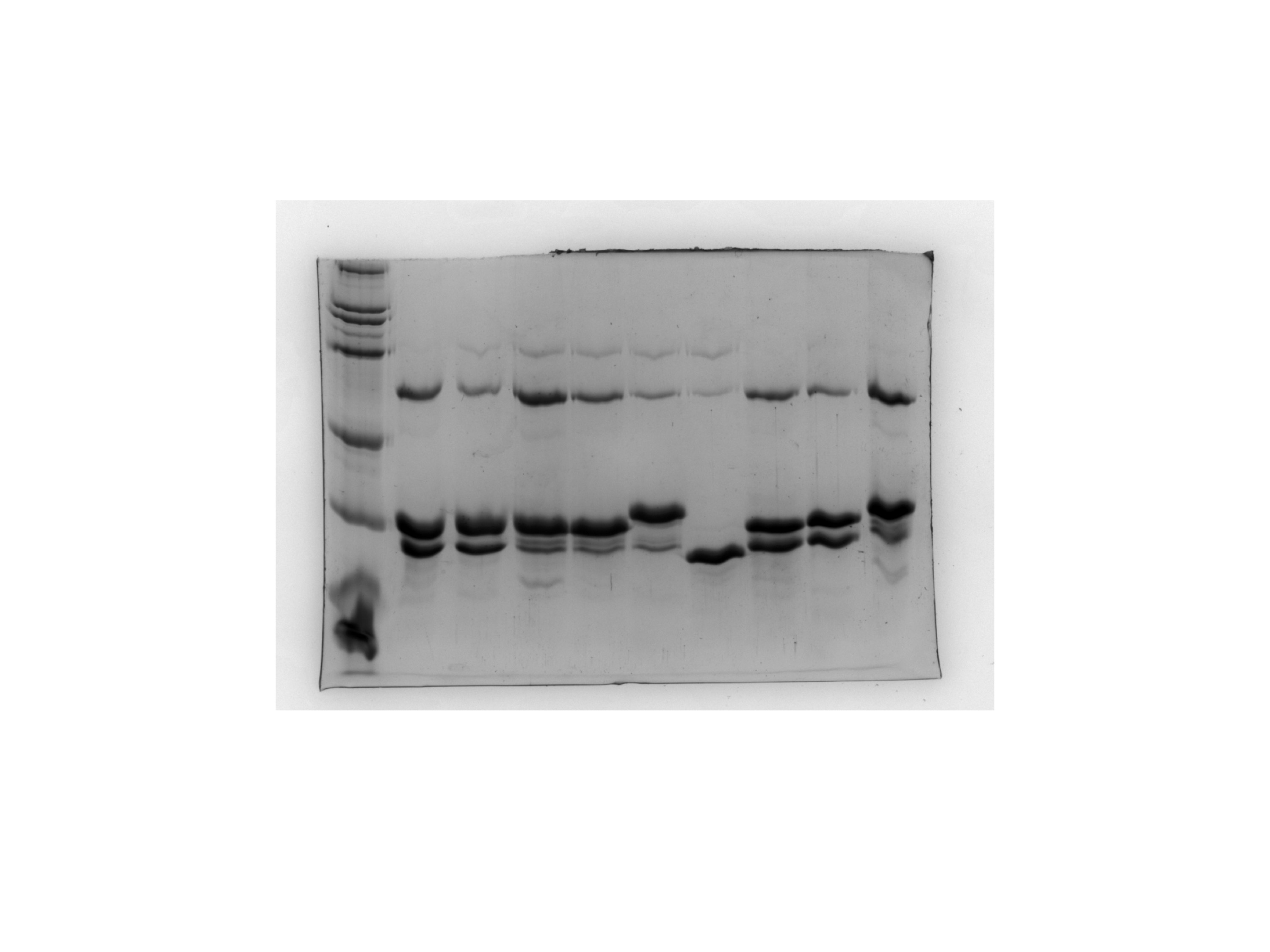

Supplement: Figure 2—figure supplement 2—source data 1. [file elife-79736-fig2-figsupp2-data1.zip › Figure 2-figure supplement 2-source data 1/Figure 2-figure supplement 2b_2.jpg]

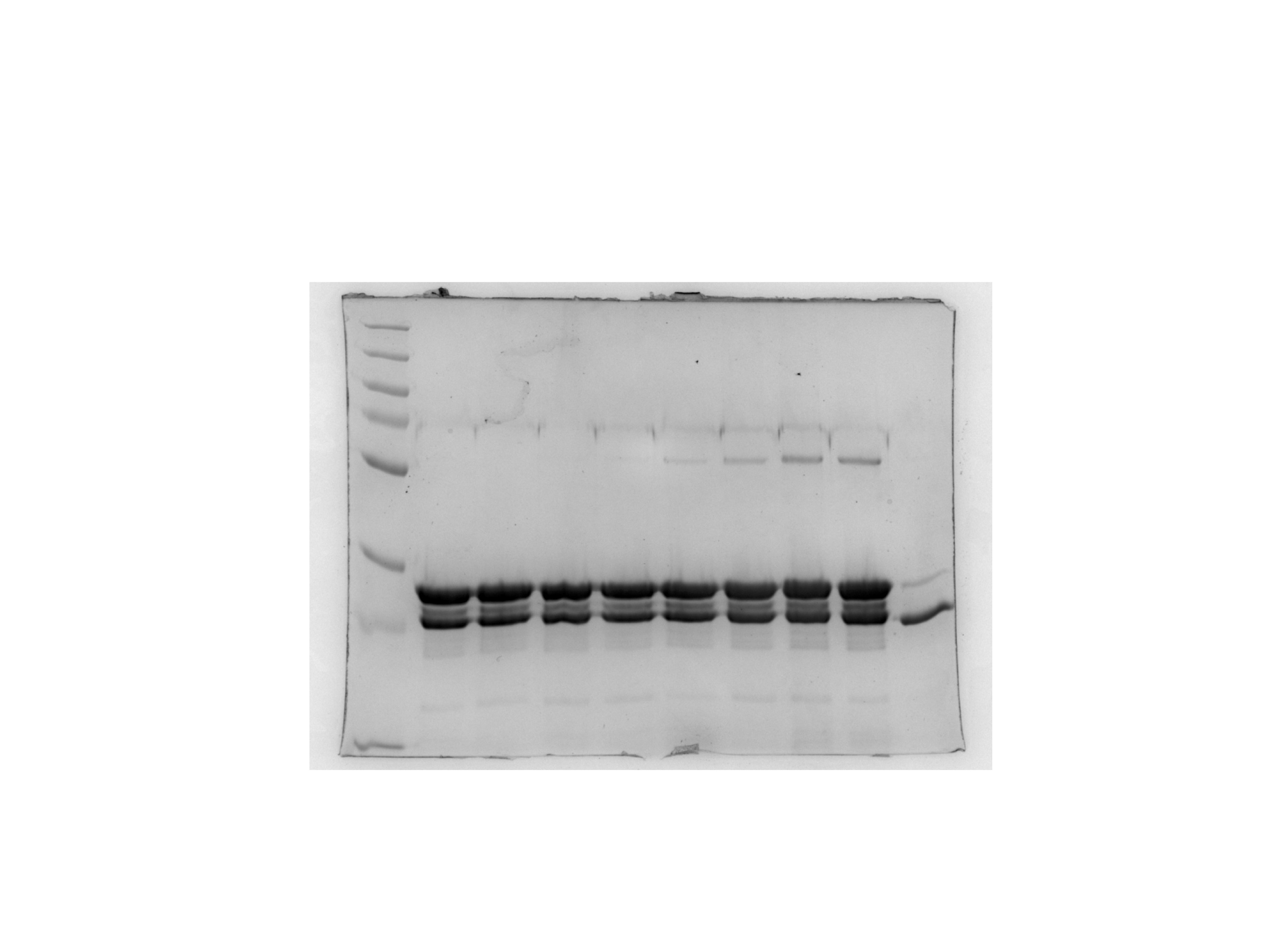

Supplement: Figure 2—figure supplement 2—source data 2. [file elife-79736-fig2-figsupp2-data2.zip › Figure 2-figure supplement 2-source data 2/Figure2-figure supplement 2c.jpg]

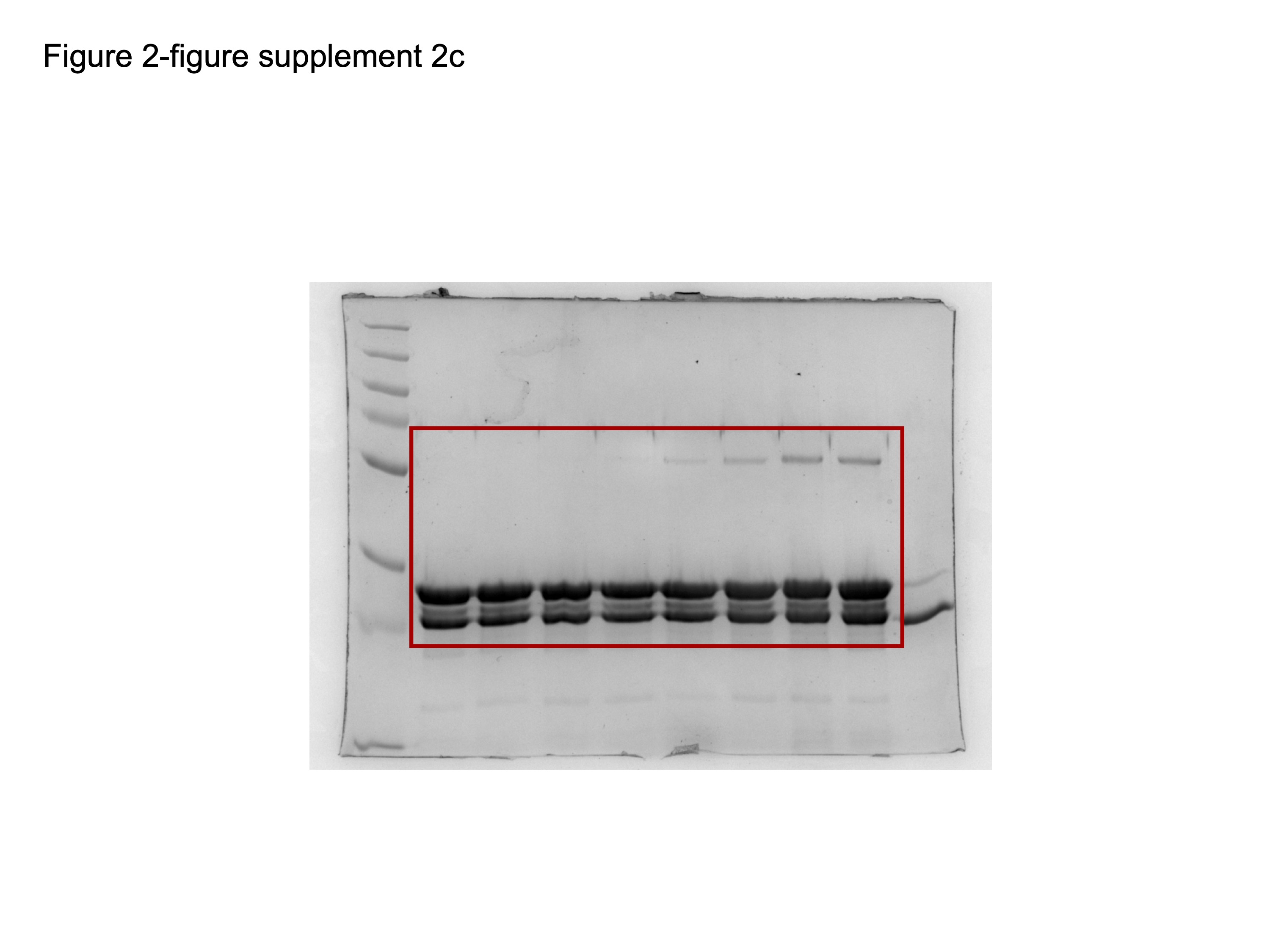

Supplement: Figure 2—figure supplement 2—source data 2. [file elife-79736-fig2-figsupp2-data2.zip › Figure 2-figure supplement 2-source data 2/Uncropped_Labeled_Gel_Figure 2-figure supplement 2c.jpg]

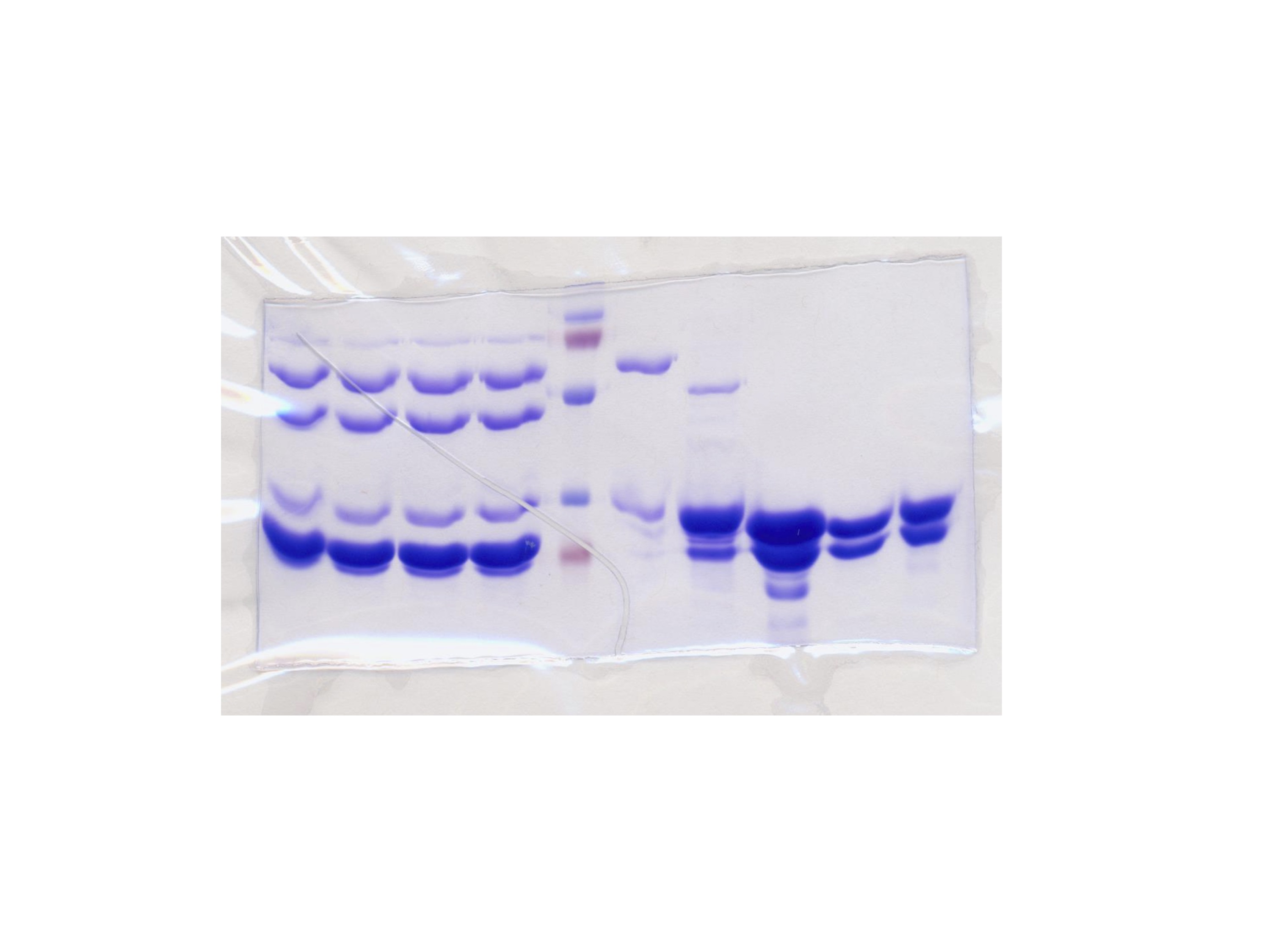

Supplement: Figure 2—figure supplement 3—source data 1. [file elife-79736-fig2-figsupp3-data1.zip › Figure 2-figure supplement 3-source data 1/Figure 2-Figure supplement 3a_SYT16_PME-1 ╬öN18.jpg]

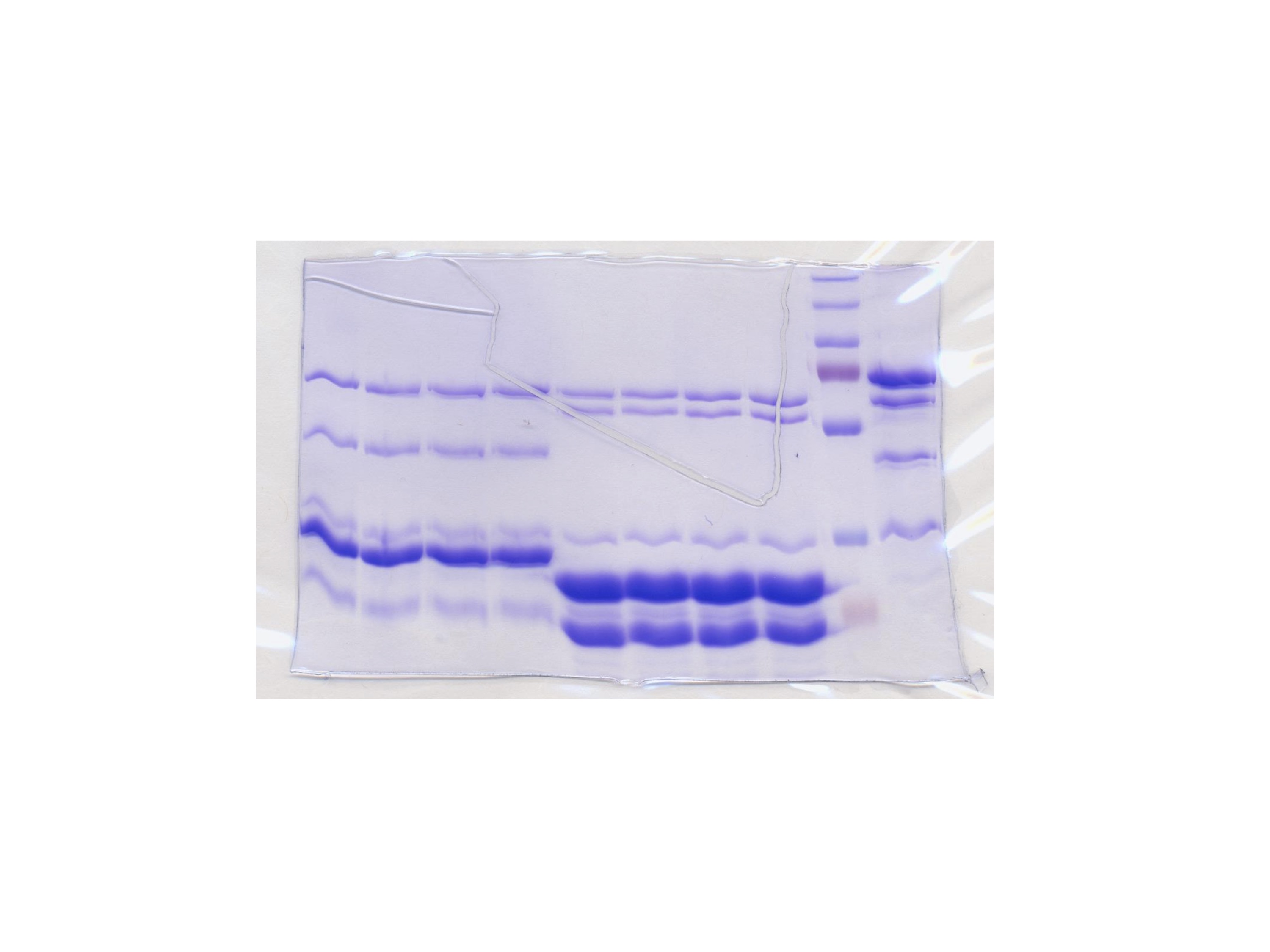

Supplement: Figure 2—figure supplement 3—source data 1. [file elife-79736-fig2-figsupp3-data1.zip › Figure 2-figure supplement 3-source data 1/Figure 2-Figure supplement 3a_CRTC3_PME-1 ╬öN18 and Cdc6_PME-1 ╬öN18.jpg]

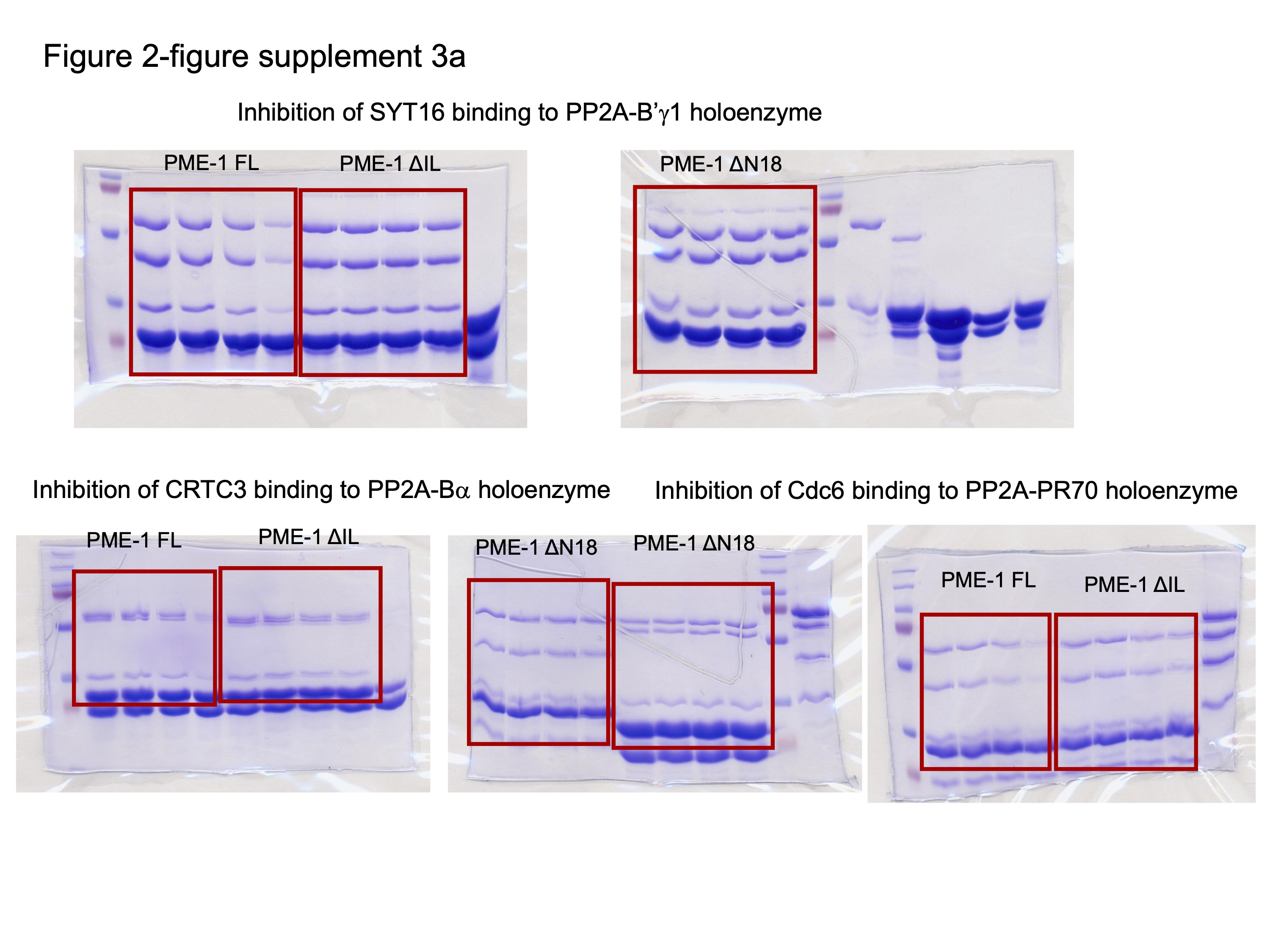

Supplement: Figure 2—figure supplement 3—source data 1. [file elife-79736-fig2-figsupp3-data1.zip › Figure 2-figure supplement 3-source data 1/Uncropped_Labeled_Gel_Figure 2-figure supplement 3a.jpg]

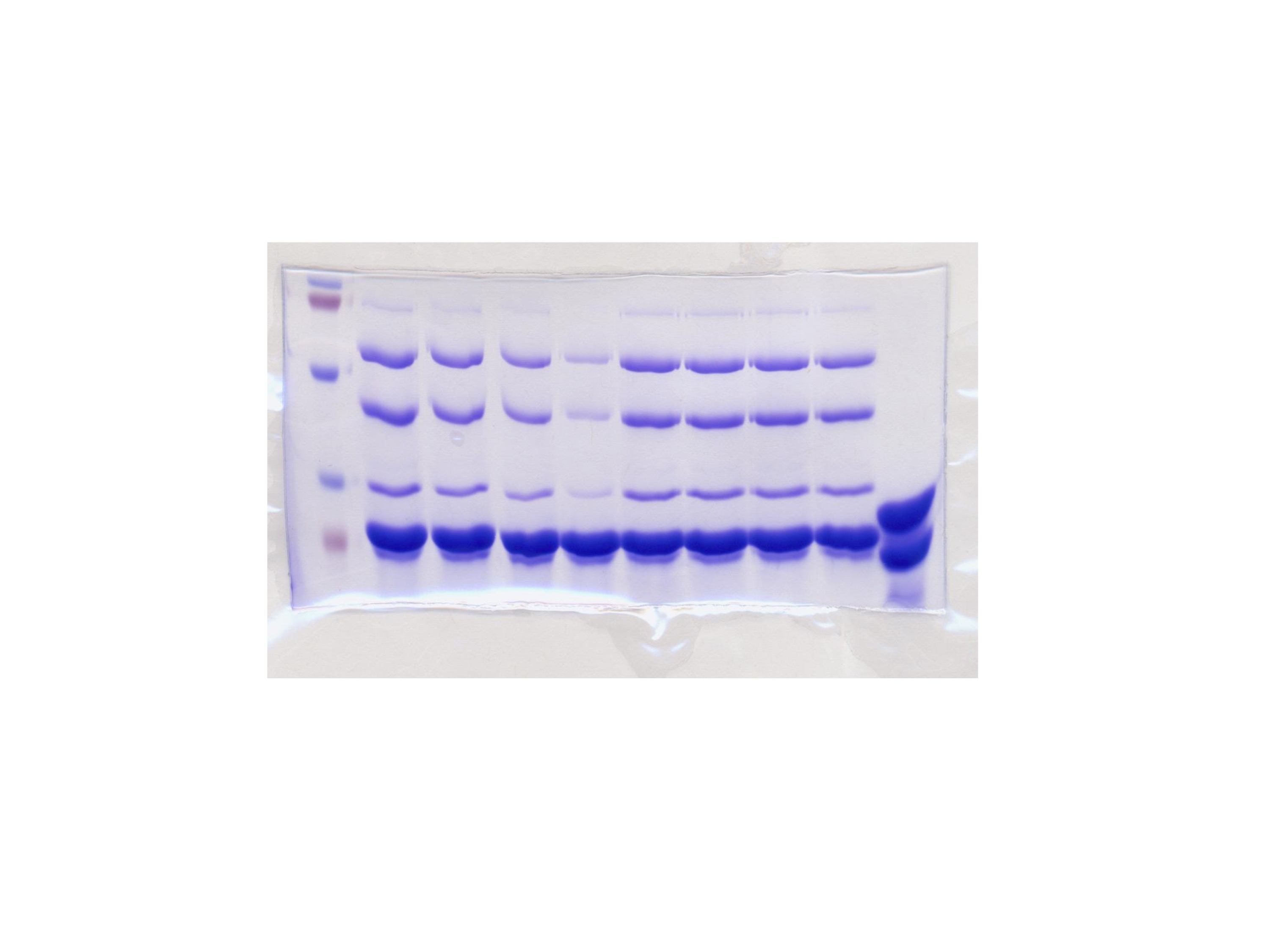

Supplement: Figure 2—figure supplement 3—source data 1. [file elife-79736-fig2-figsupp3-data1.zip › Figure 2-figure supplement 3-source data 1/Figure 2-Figure supplement 3a_SYT16_PME-1 FL and ╬öIL.jpg]

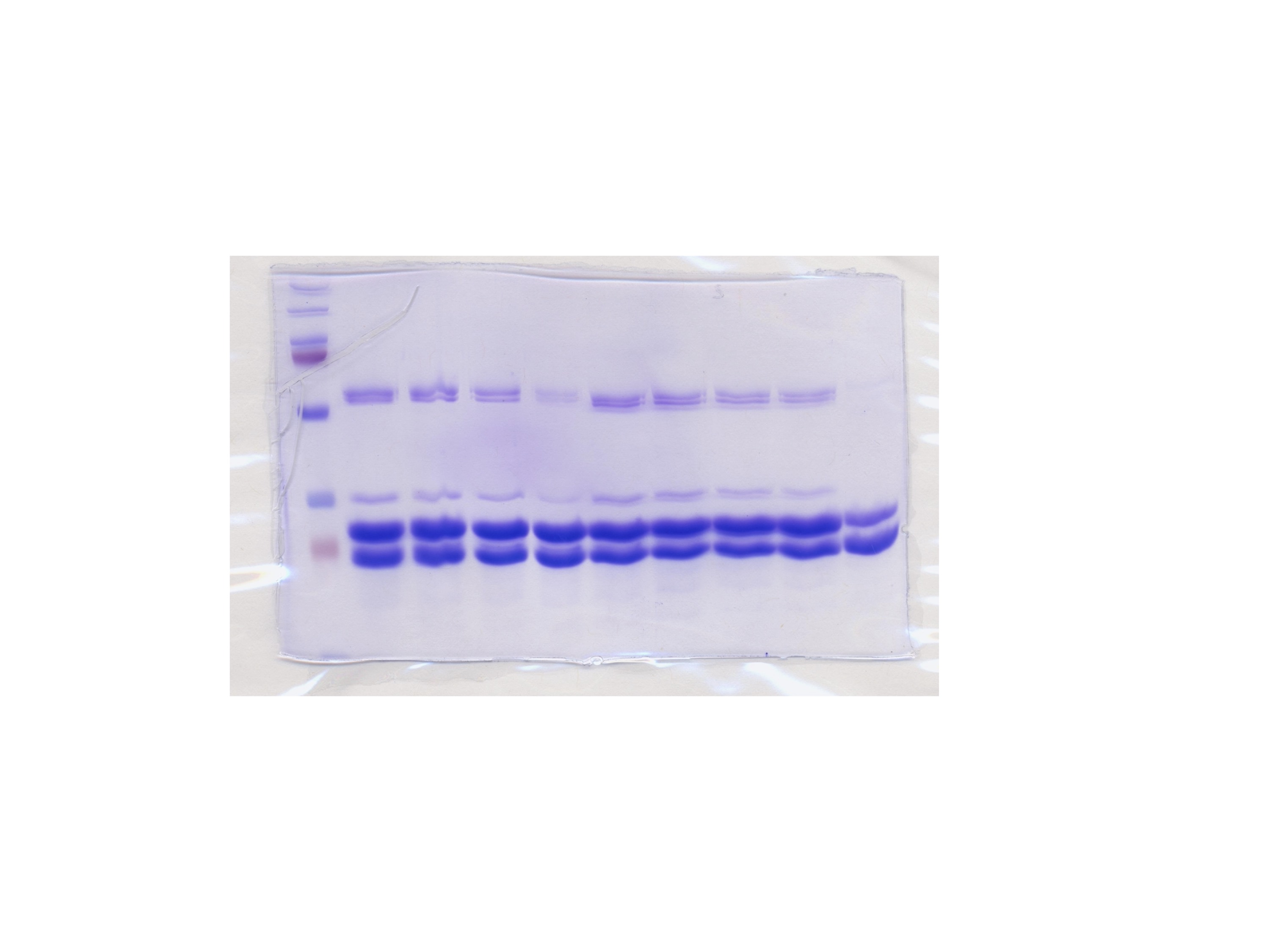

Supplement: Figure 2—figure supplement 3—source data 1. [file elife-79736-fig2-figsupp3-data1.zip › Figure 2-figure supplement 3-source data 1/Figure 2-Figure supplement 3a_CRTC3_PME-1 FL and ╬öIL.jpg]

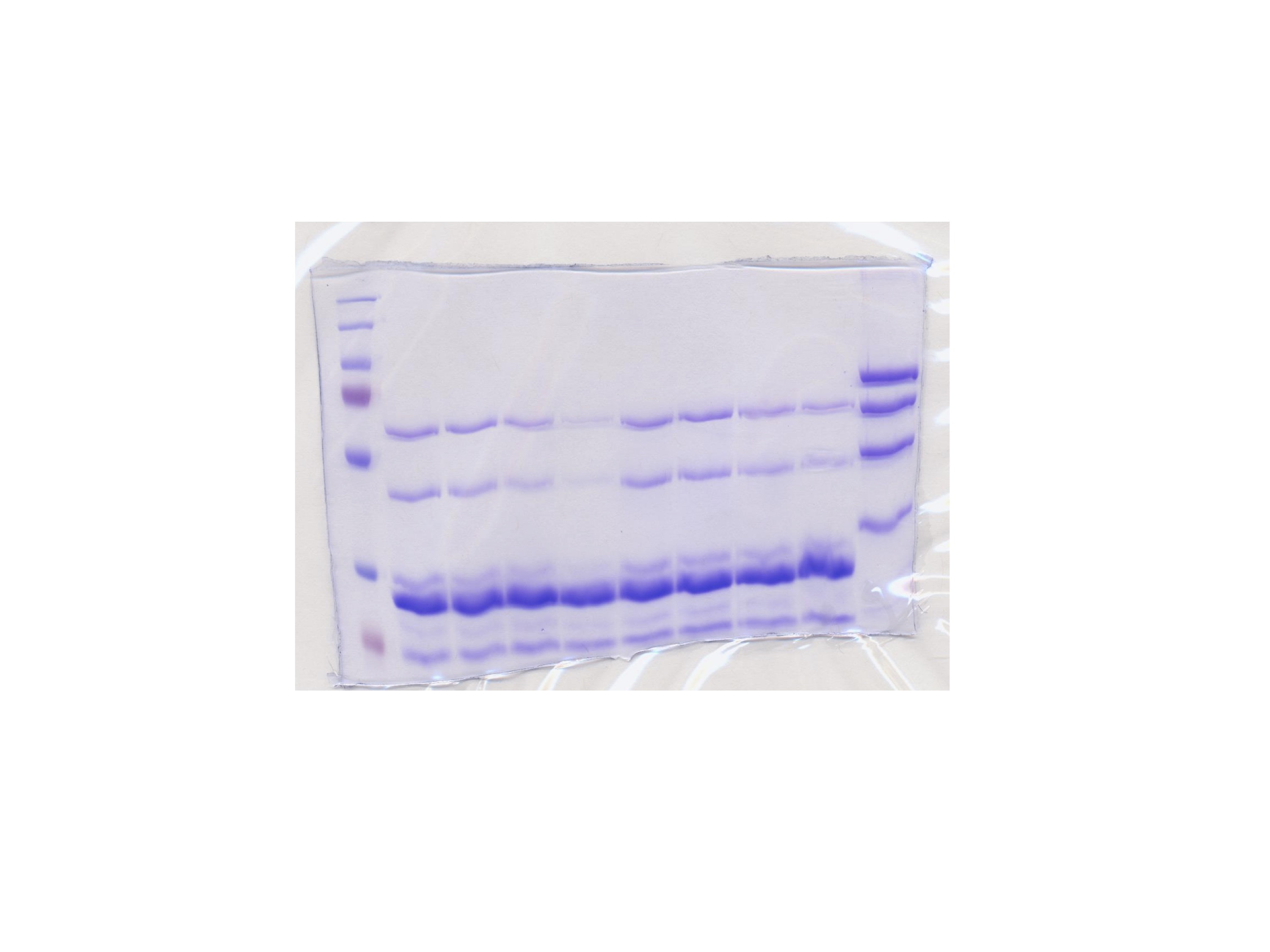

Supplement: Figure 2—figure supplement 3—source data 1. [file elife-79736-fig2-figsupp3-data1.zip › Figure 2-figure supplement 3-source data 1/Figure 2-Figure supplement 3a_Cdc6_PME-1 FL and ╬öIL.jpg]

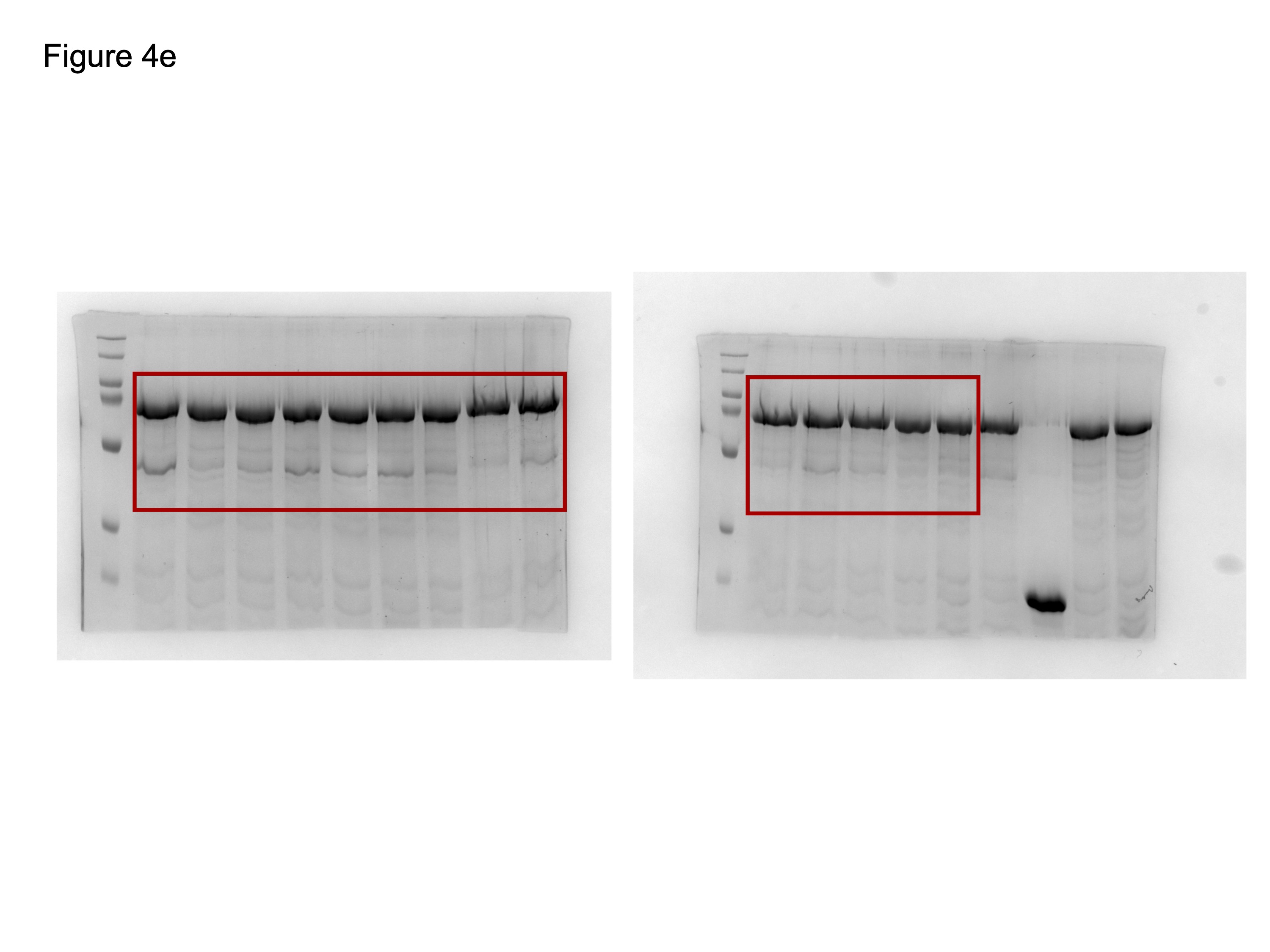

Supplement: Figure 4—source data 1. [file elife-79736-fig4-data1.zip › Figure 4-source data 1/Uncropped_Labeled_Gel_Figure 4e.jpg]

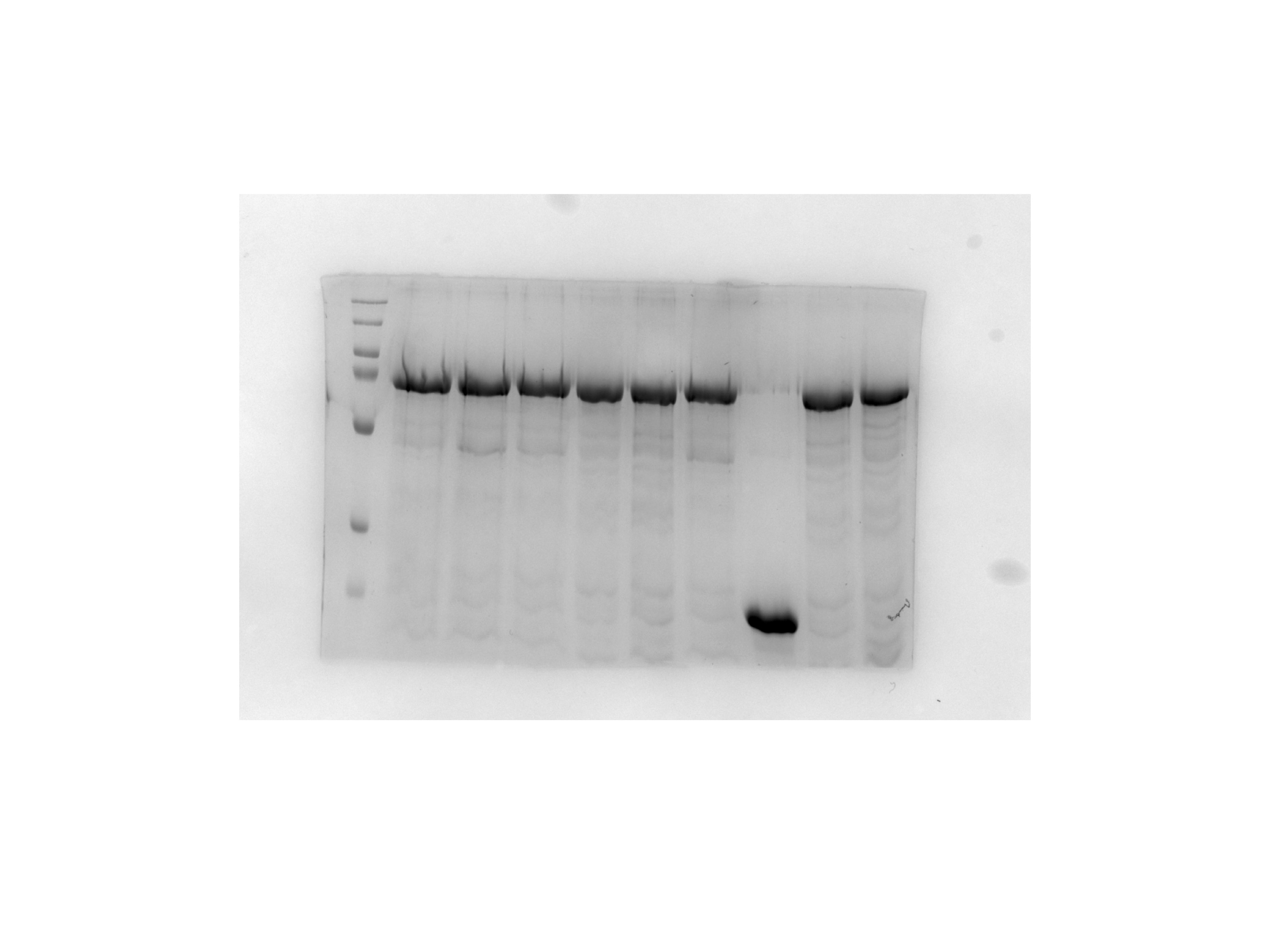

Supplement: Figure 4—source data 1. [file elife-79736-fig4-data1.zip › Figure 4-source data 1/Figure 4e_2.jpg]

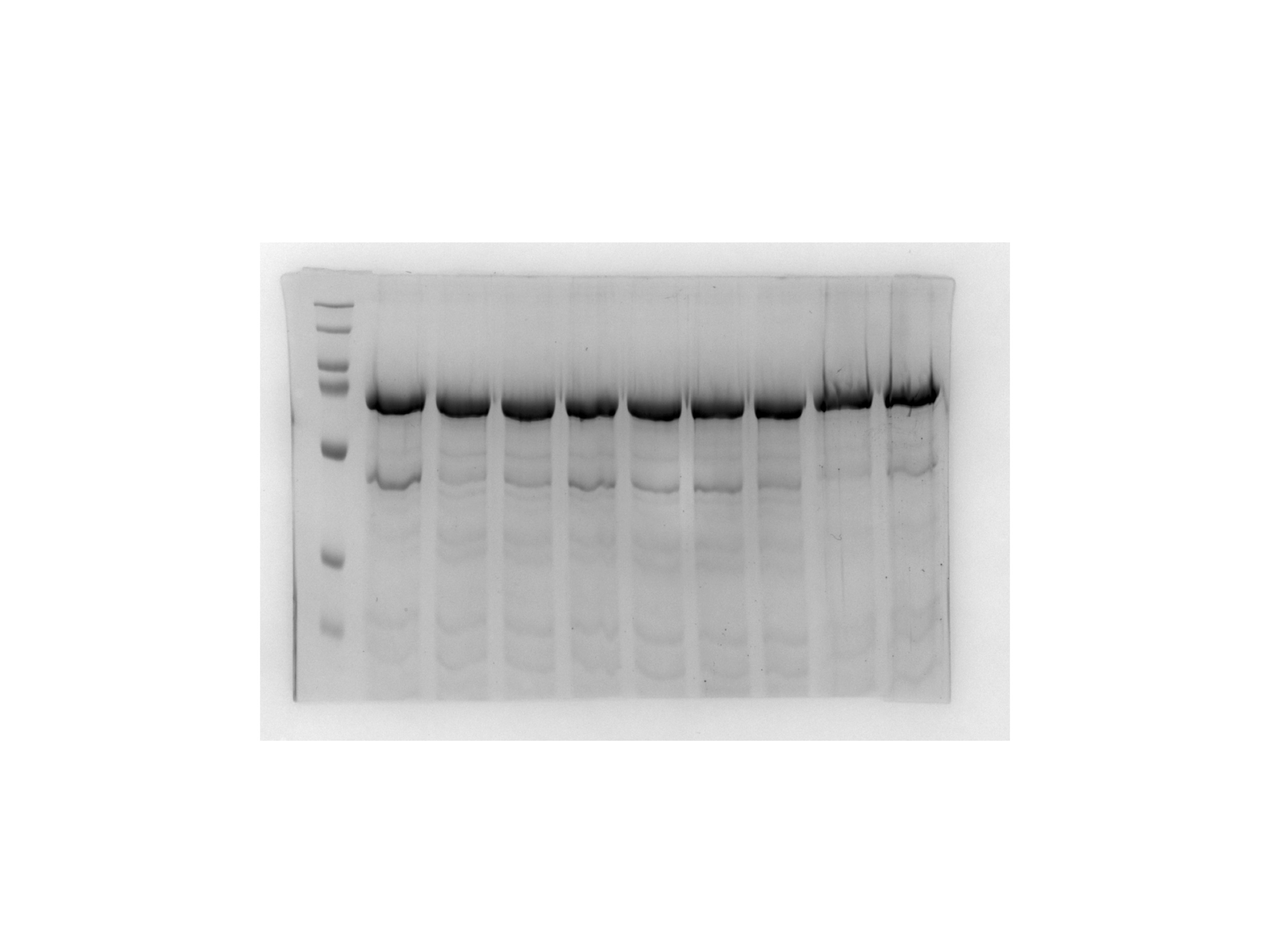

Supplement: Figure 4—source data 1. [file elife-79736-fig4-data1.zip › Figure 4-source data 1/Figure 4e_1.jpg]

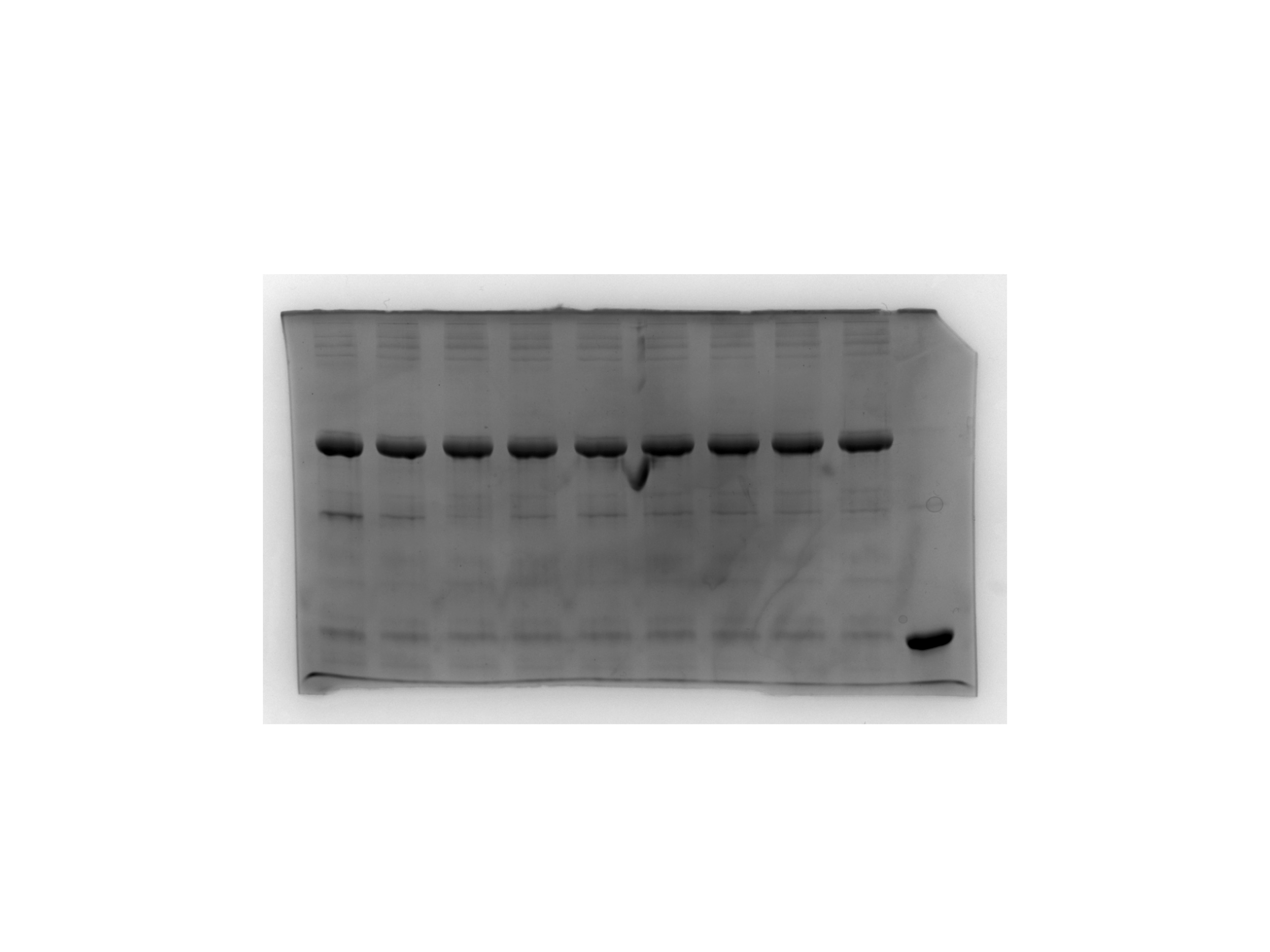

Supplement: Figure 4—source data 2. [file elife-79736-fig4-data2.zip › Figure 4-source data 2/Figure 4f.jpg]

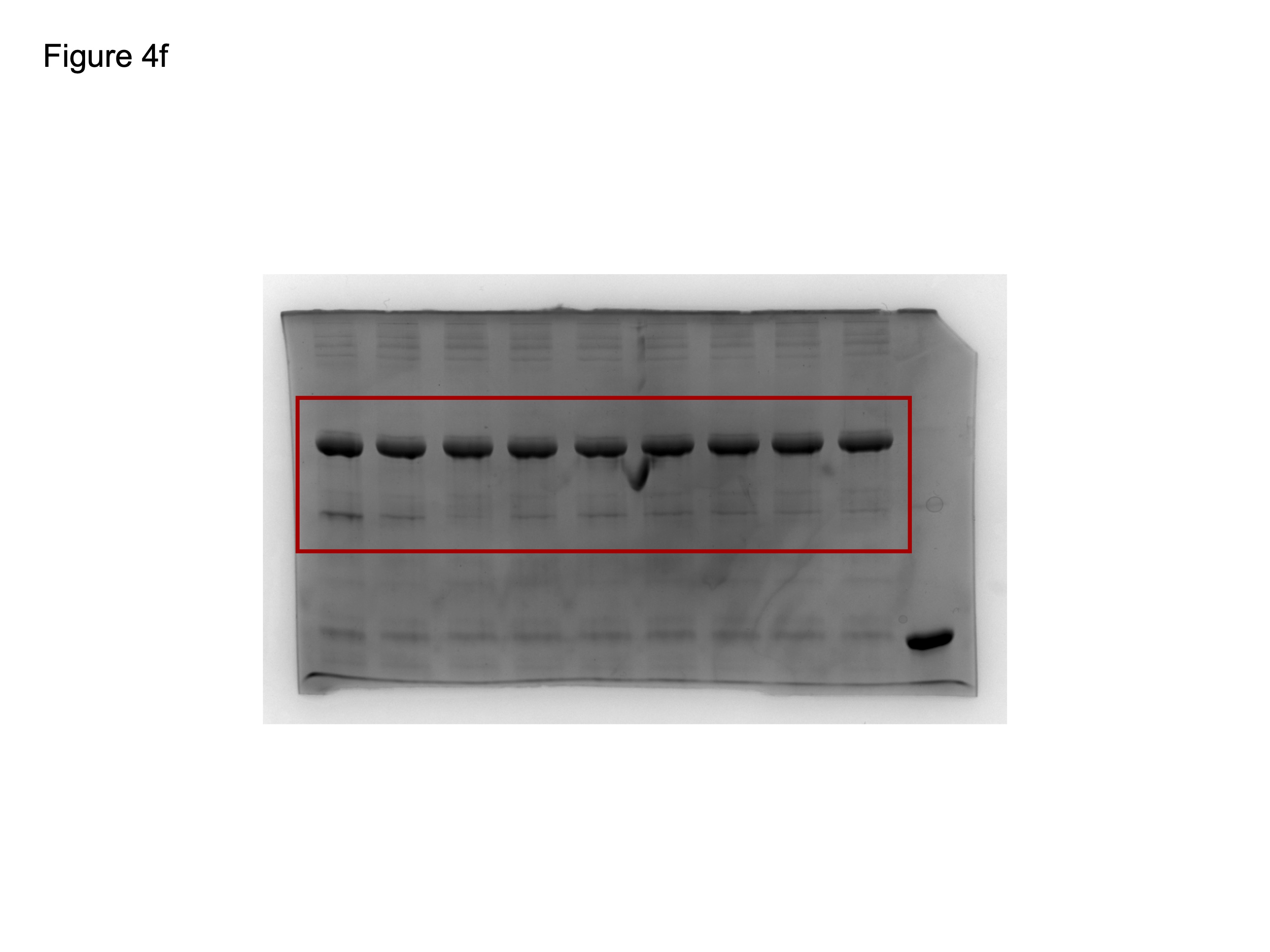

Supplement: Figure 4—source data 2. [file elife-79736-fig4-data2.zip › Figure 4-source data 2/Uncropped_Labeled_Gel_Figure 4e.jpg]

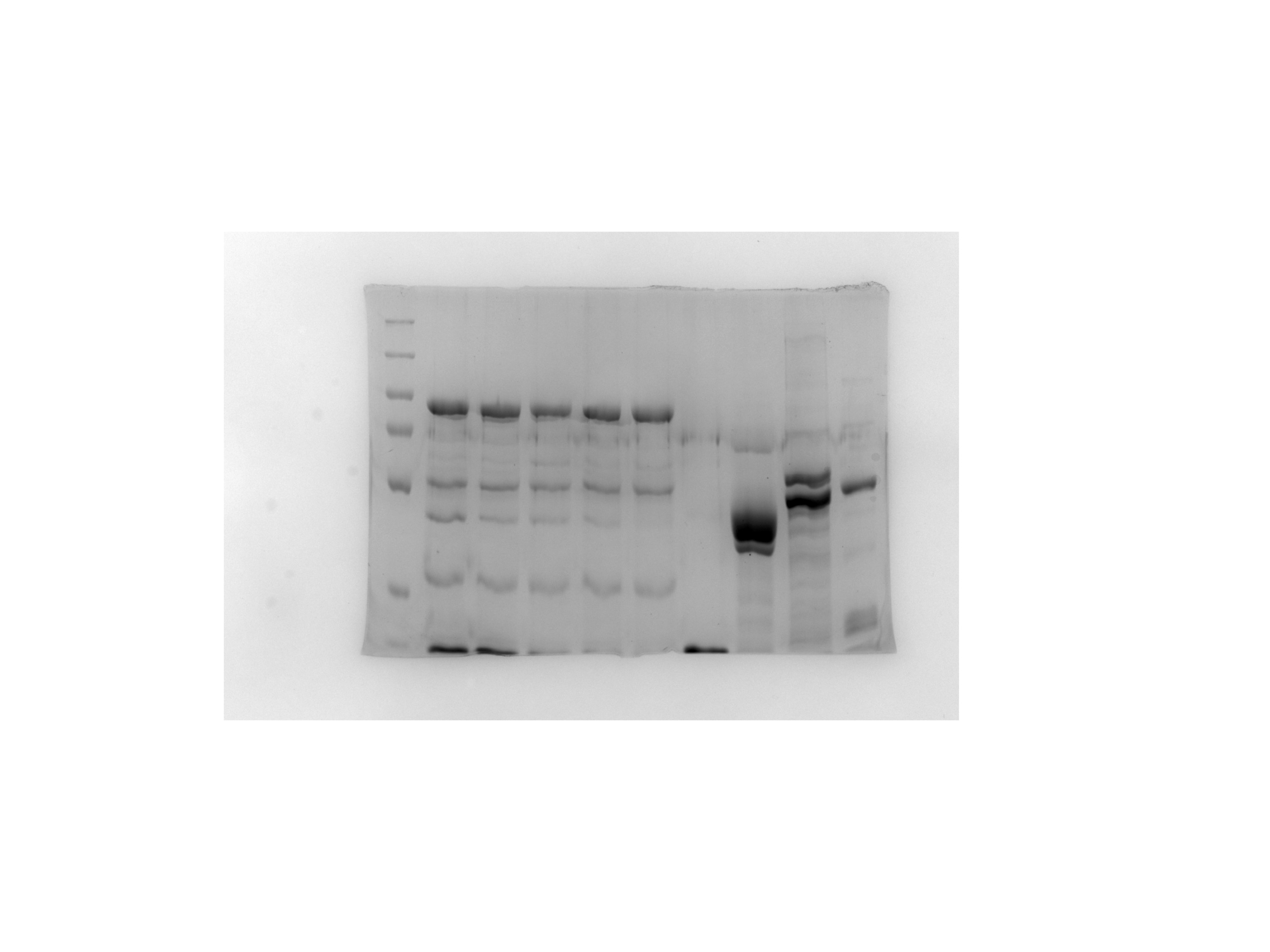

Supplement: Figure 4—source data 3. [file elife-79736-fig4-data3.zip › Figure 4-source data 3/Figure 4g.jpg]

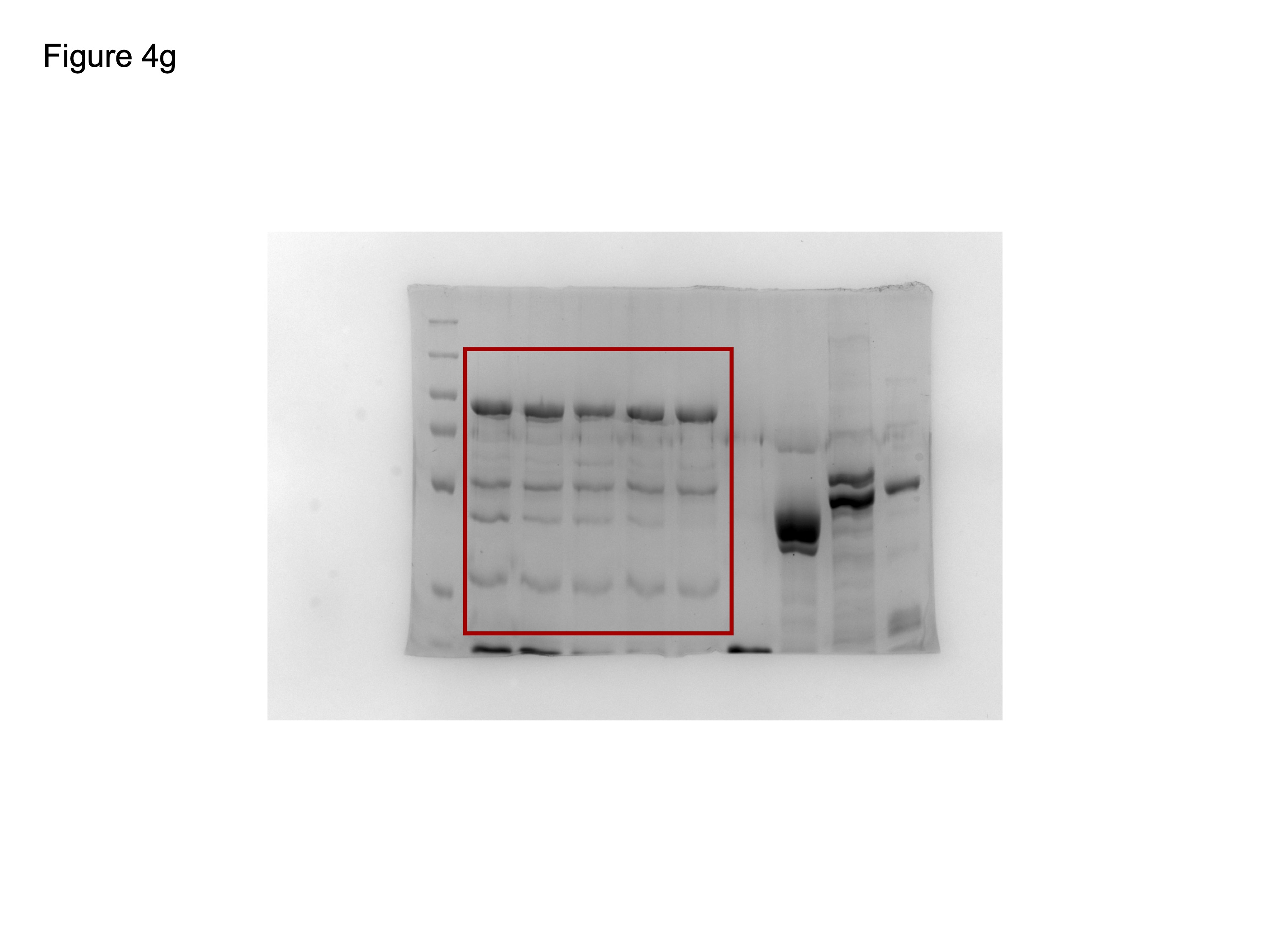

Supplement: Figure 4—source data 3. [file elife-79736-fig4-data3.zip › Figure 4-source data 3/Uncropped_Labeled_Gel_Figure 4g.jpg]

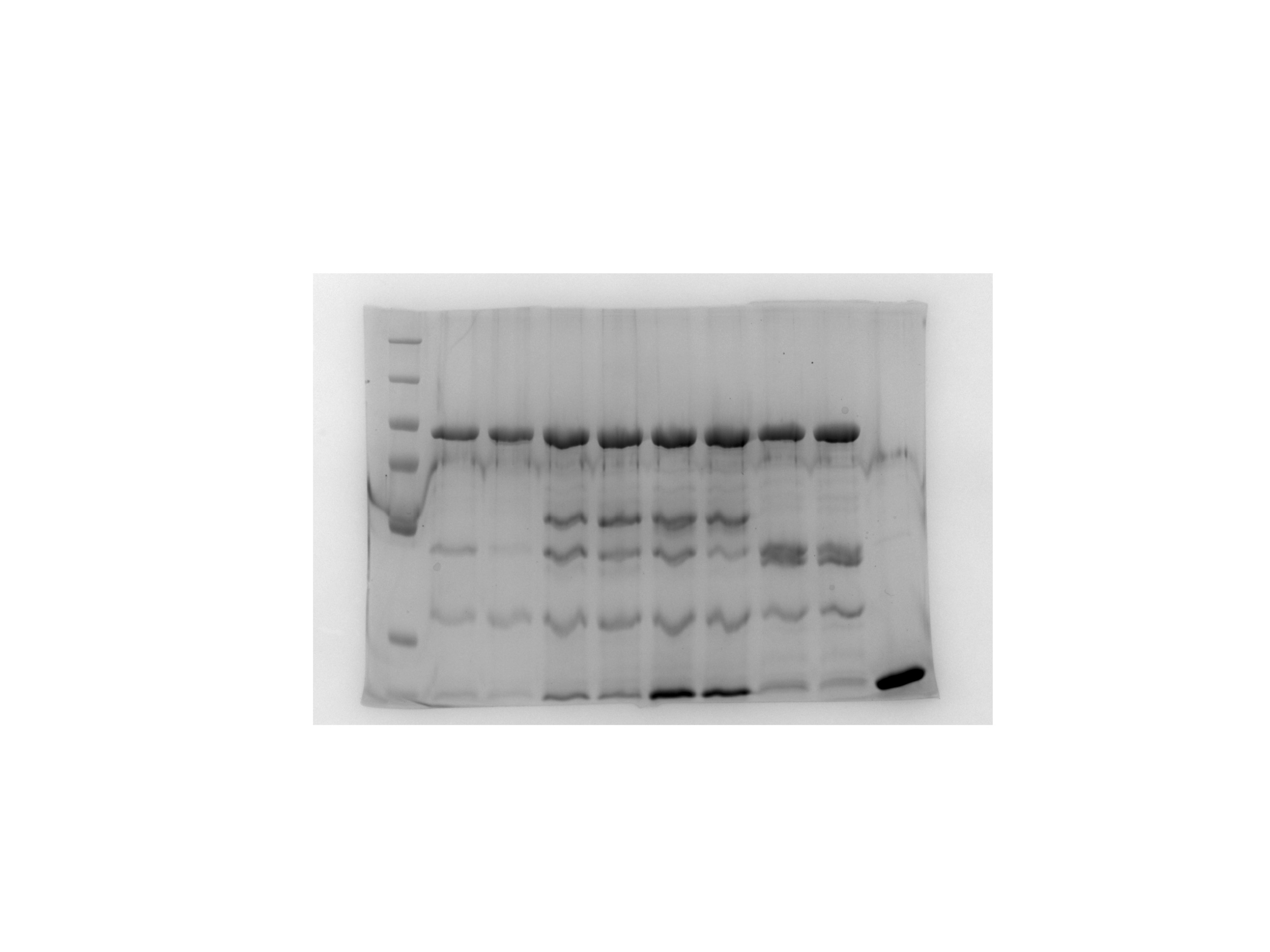

Supplement: Figure 5—source data 2. [file elife-79736-fig5-data2.zip › Figure 5-source data 2/Figure 5b .jpg]

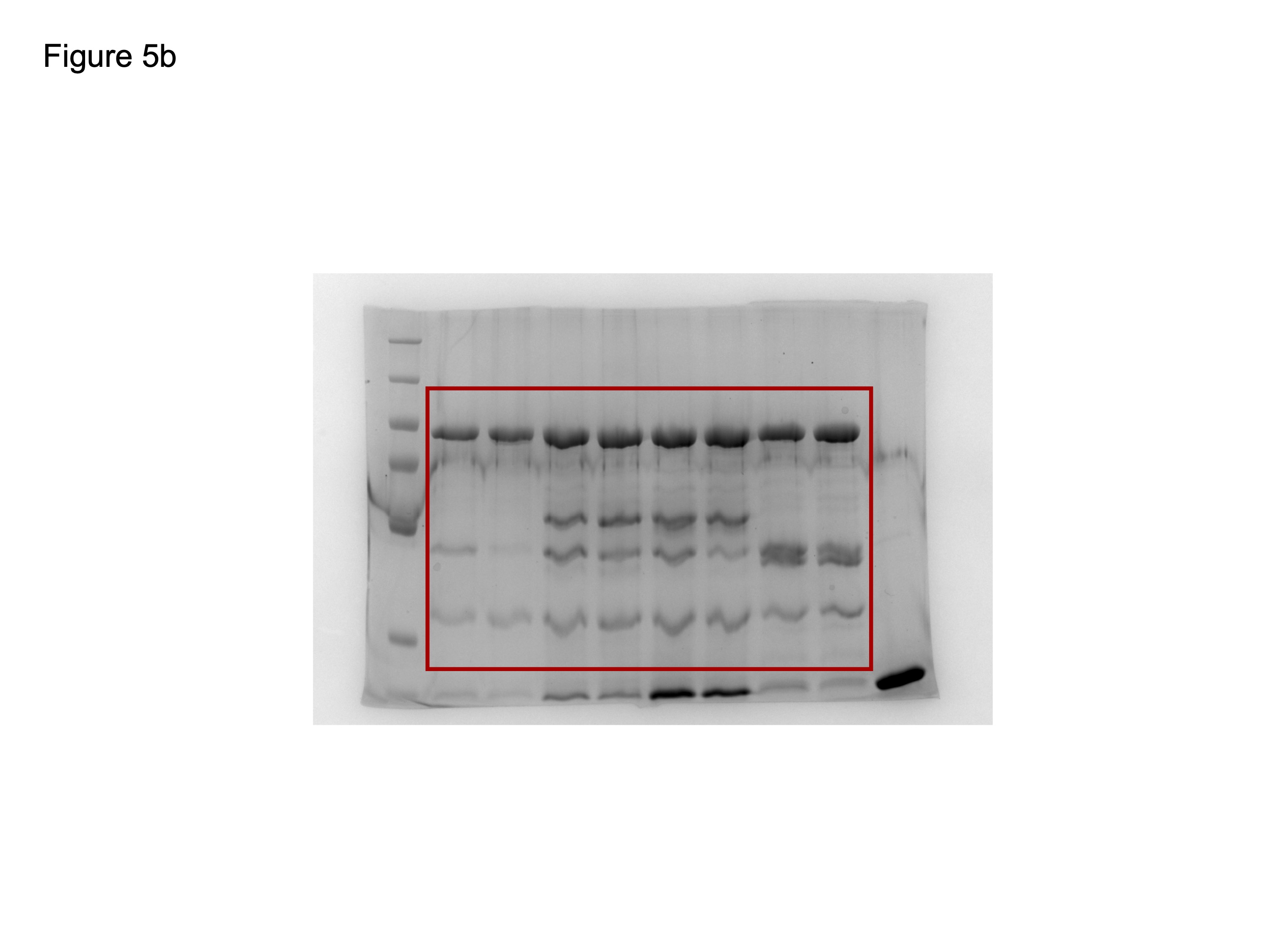

Supplement: Figure 5—source data 2. [file elife-79736-fig5-data2.zip › Figure 5-source data 2/Uncropped_Labeled_Gel_Figure 5b.jpg]

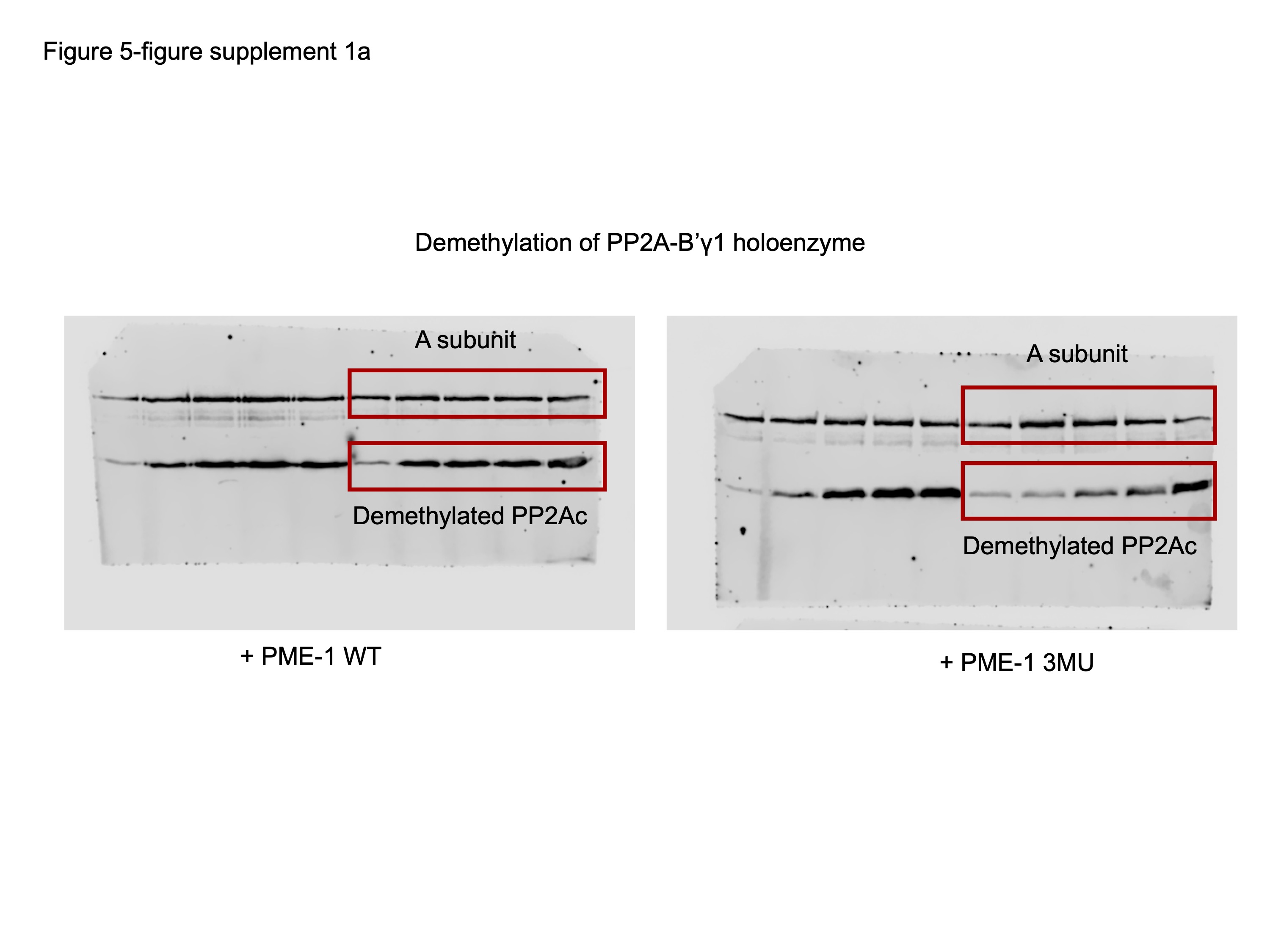

Supplement: Figure 5—figure supplement 1—source data 1. [file elife-79736-fig5-figsupp1-data1.zip › Figure 5-figure supplement 1-source data 1/Uncropped_Labeled_Gel_Figure 5-figure supplement 1a.jpg]

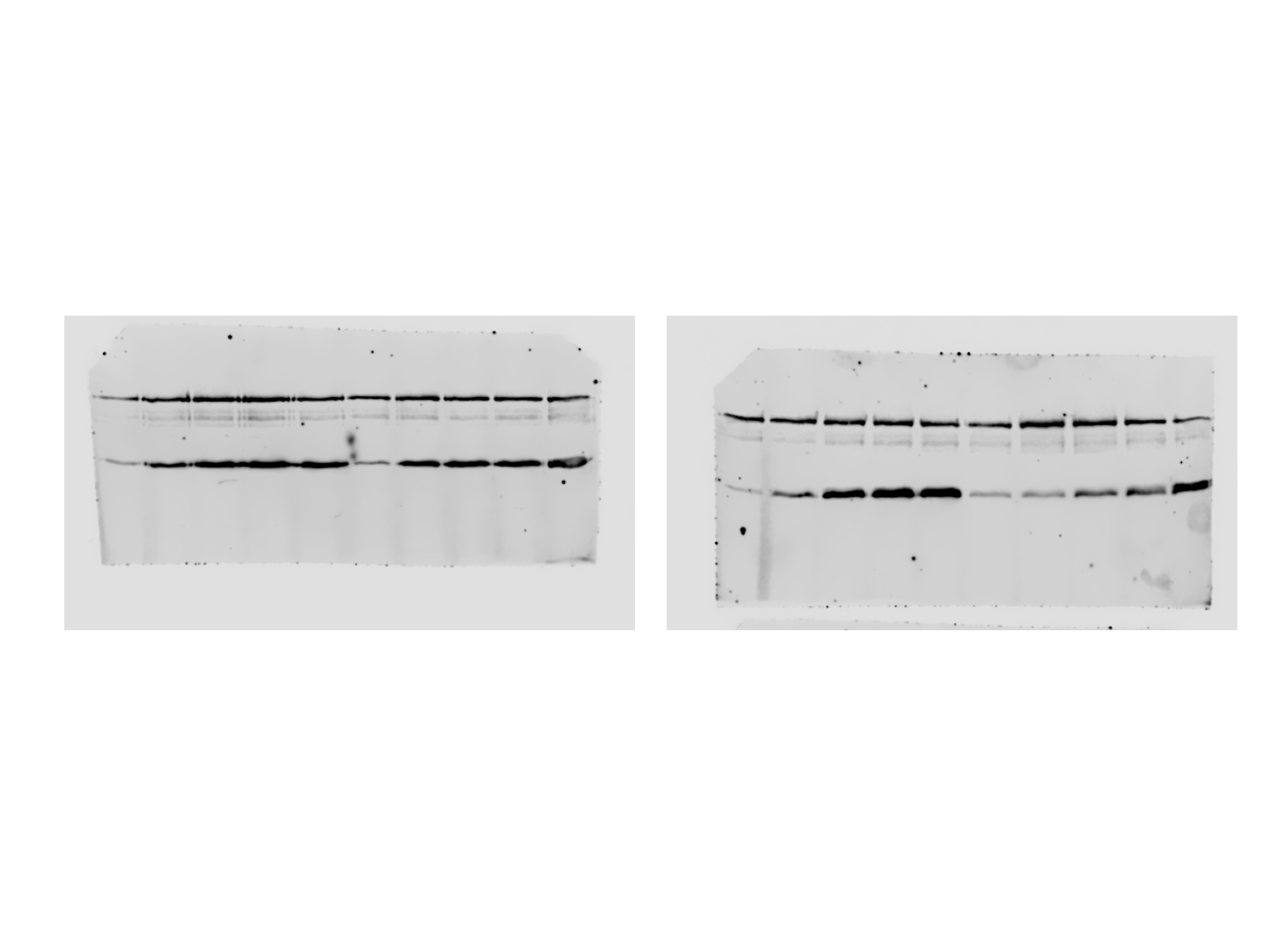

Supplement: Figure 5—figure supplement 1—source data 1. [file elife-79736-fig5-figsupp1-data1.zip › Figure 5-figure supplement 1-source data 1/Figure 5-Figure supplement 1a.jpg]

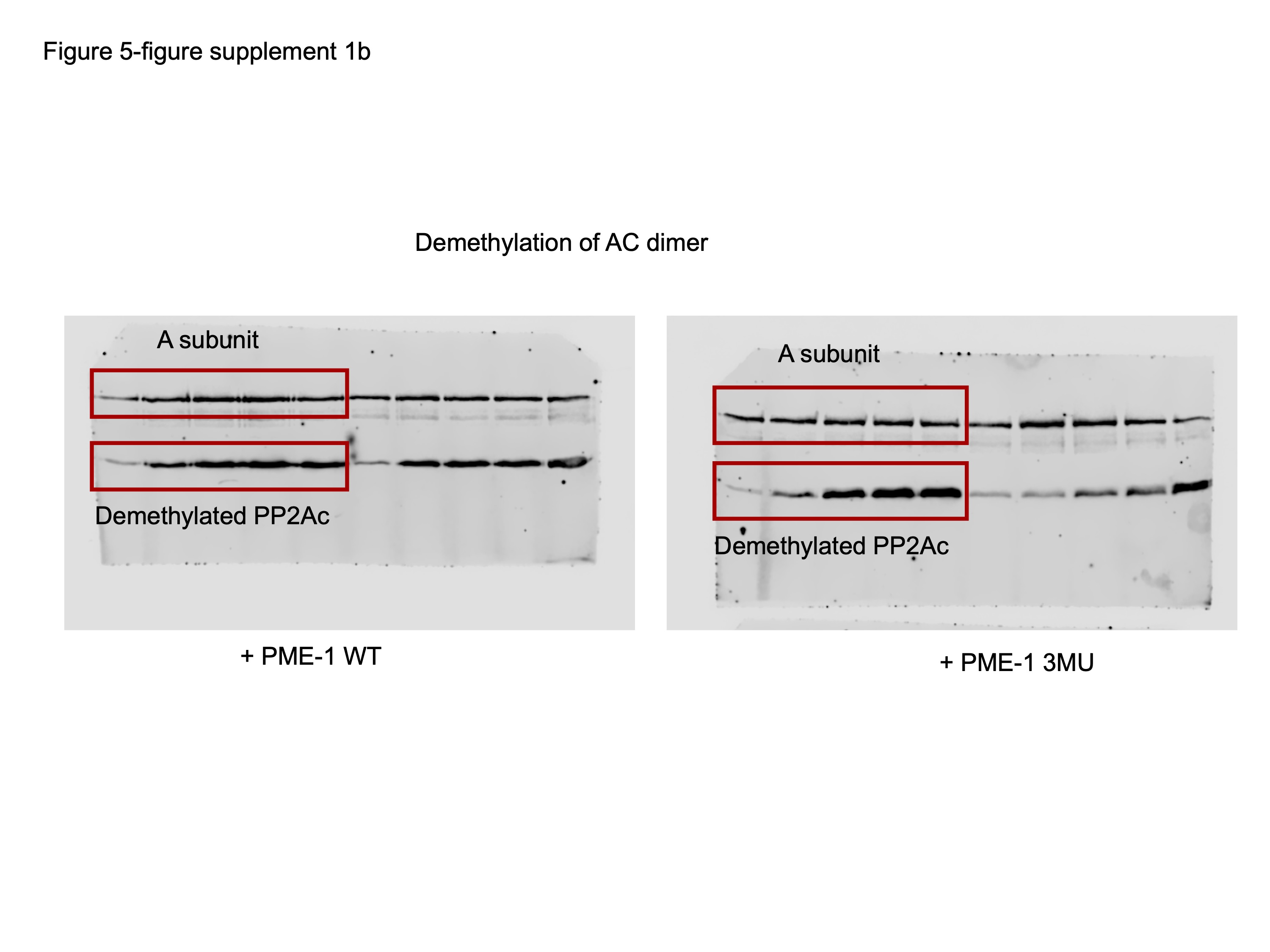

Supplement: Figure 5—figure supplement 1—source data 2. [file elife-79736-fig5-figsupp1-data2.zip › Figure 5-figure supplement 1-source data 2/Uncropped_Labeled_Gel_Figure 5-Figure supplement 1b.jpg]

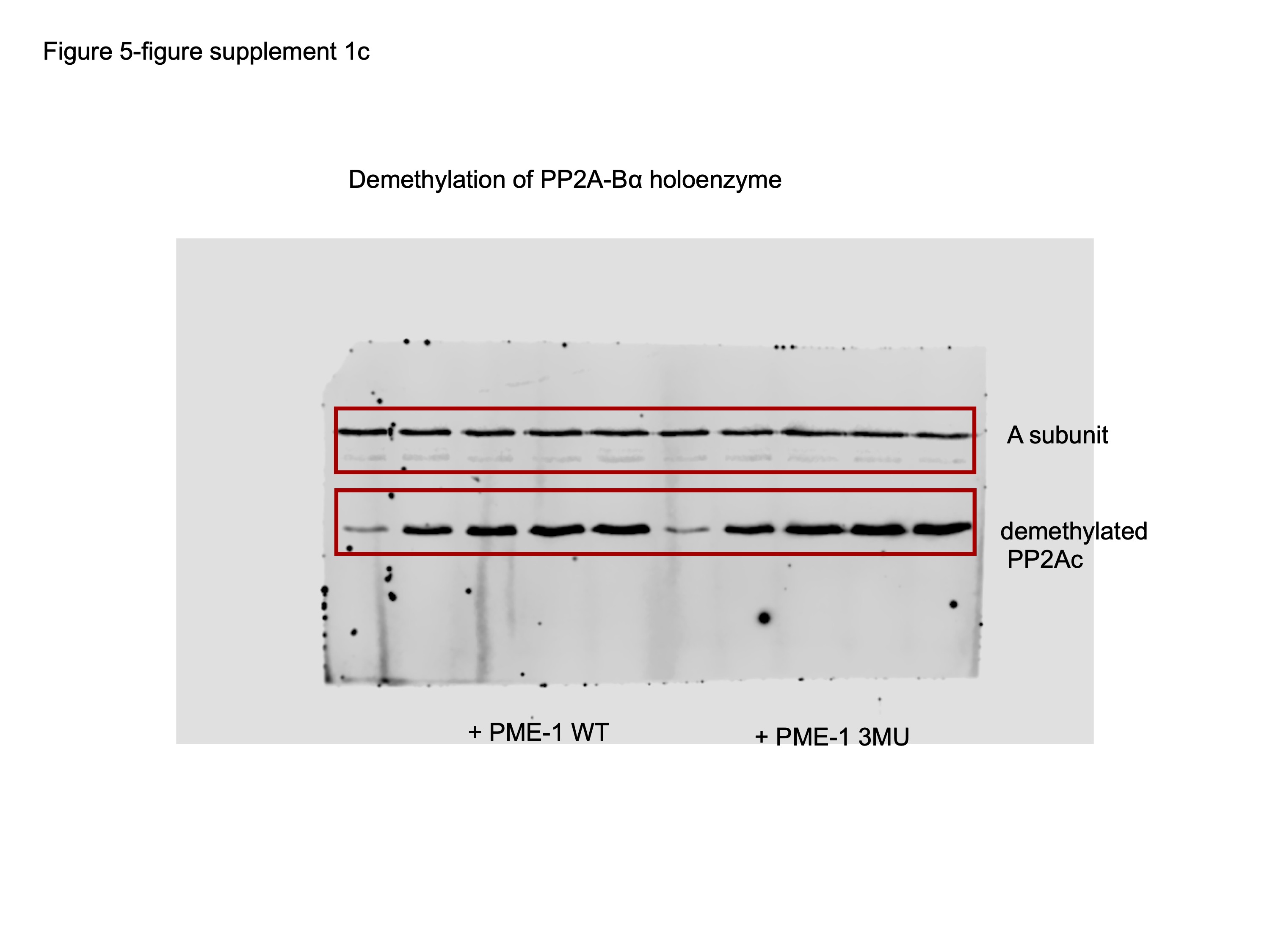

Supplement: Figure 5—figure supplement 1—source data 3. [file elife-79736-fig5-figsupp1-data3.zip › Figure 5-figure supplement 1-source data 3/Uncropped_Labeled_Gel_Figure 5-Figure supplement 1c.jpg]

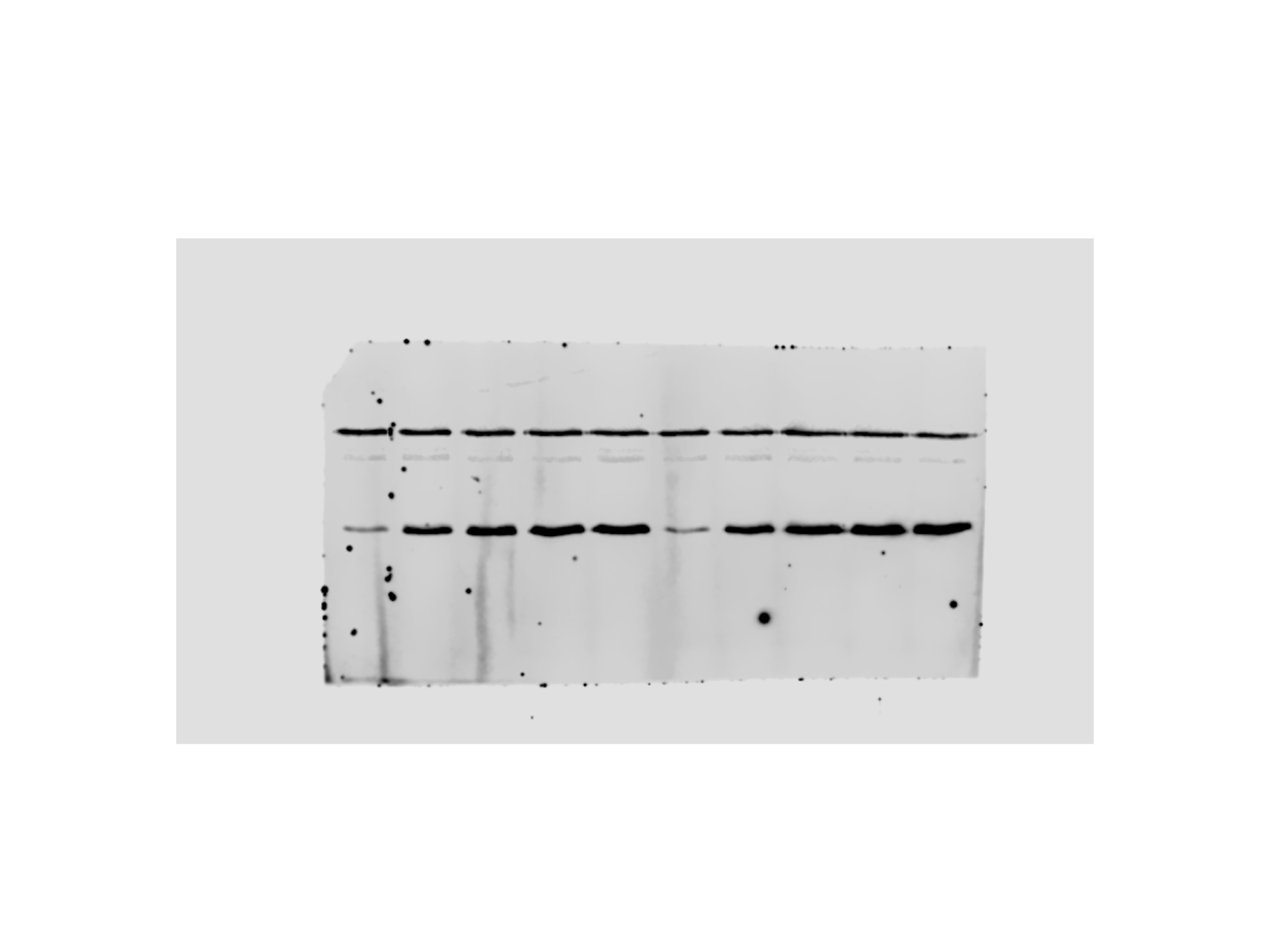

Supplement: Figure 5—figure supplement 1—source data 3. [file elife-79736-fig5-figsupp1-data3.zip › Figure 5-figure supplement 1-source data 3/Figure 5-Figure supplement 1c.jpg]

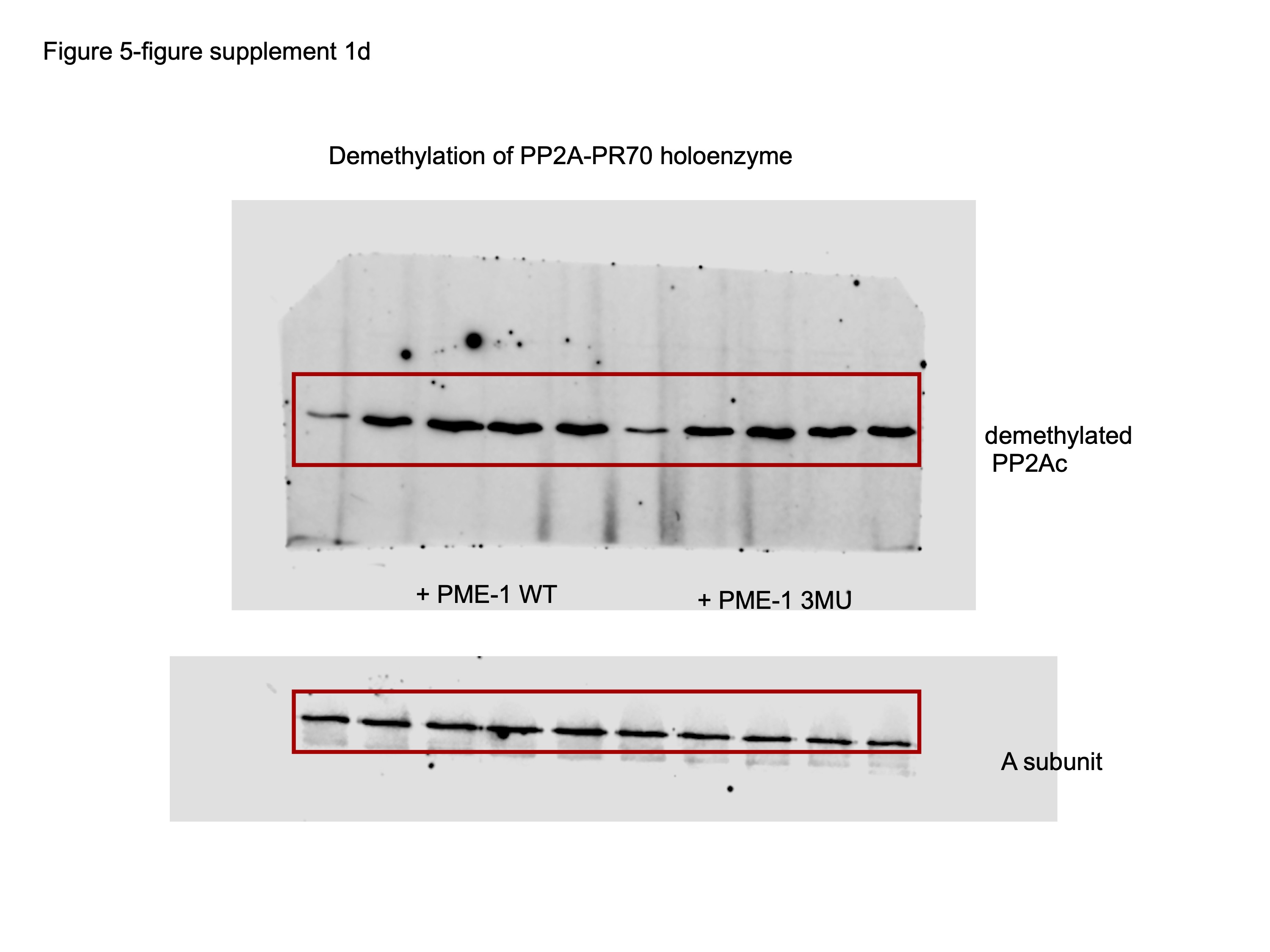

Supplement: Figure 5—figure supplement 1—source data 4. [file elife-79736-fig5-figsupp1-data4.zip › Figure 5-figure supplement 1-source data 4/Uncropped_Labeled_Gel_Figure 5-Figure supplement 1d.jpg]

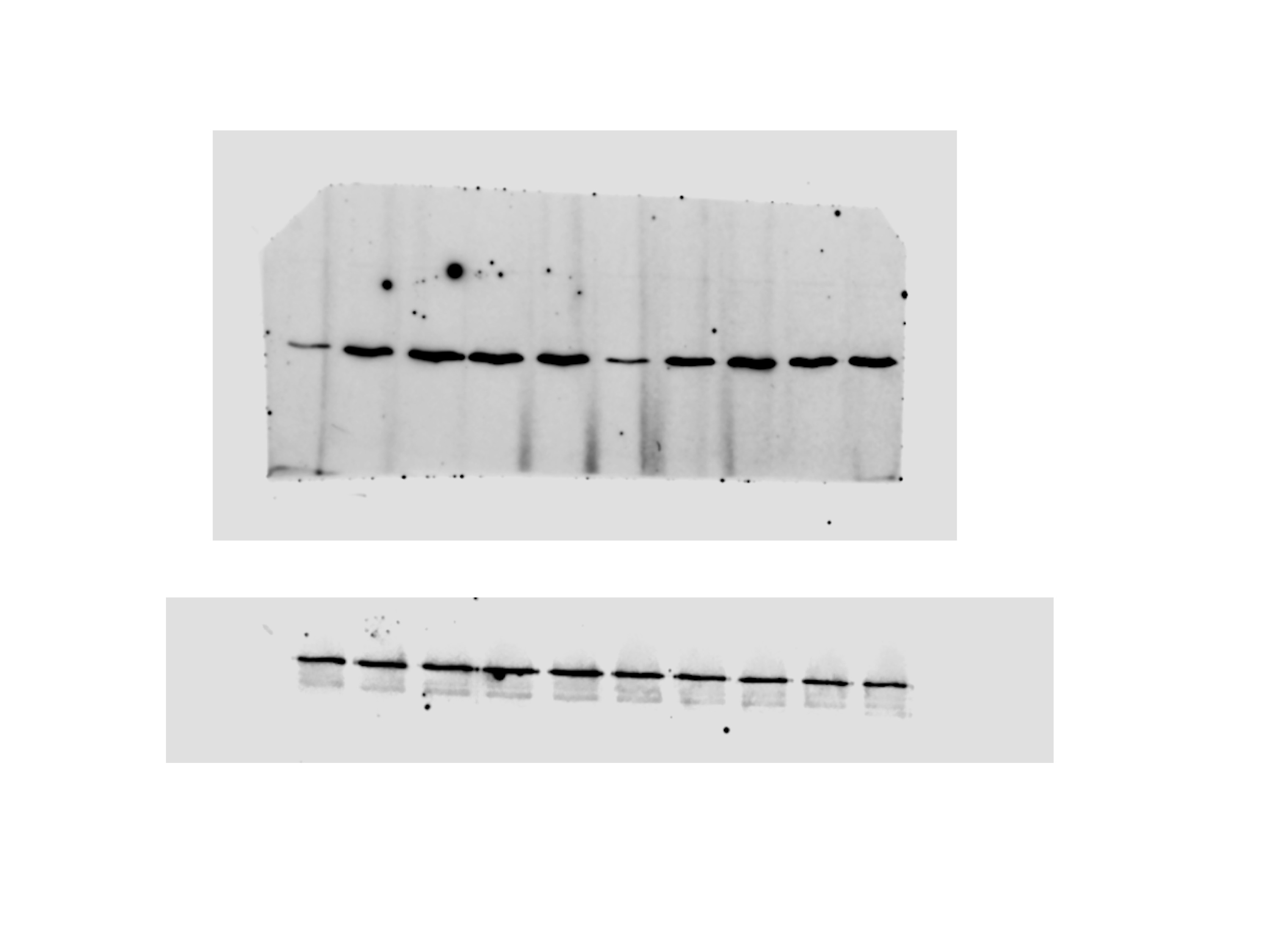

Supplement: Figure 5—figure supplement 1—source data 4. [file elife-79736-fig5-figsupp1-data4.zip › Figure 5-figure supplement 1-source data 4/Figure 5-Figure supplement 1d.jpg]

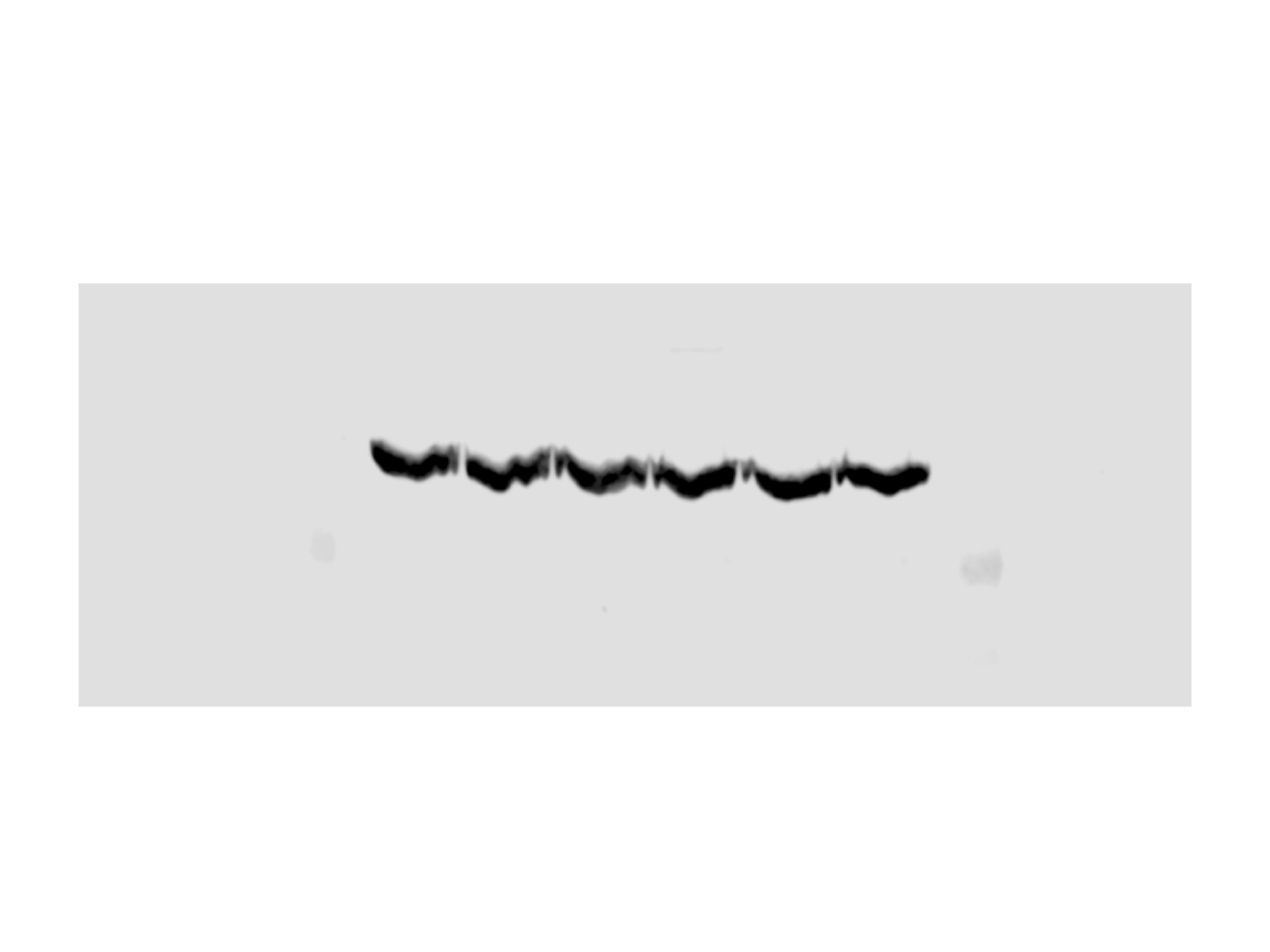

Supplement: Figure 6—source data 1. [file elife-79736-fig6-data1.zip › Figure 6-source data 1/Figure 6a_PP2Ac.jpg]

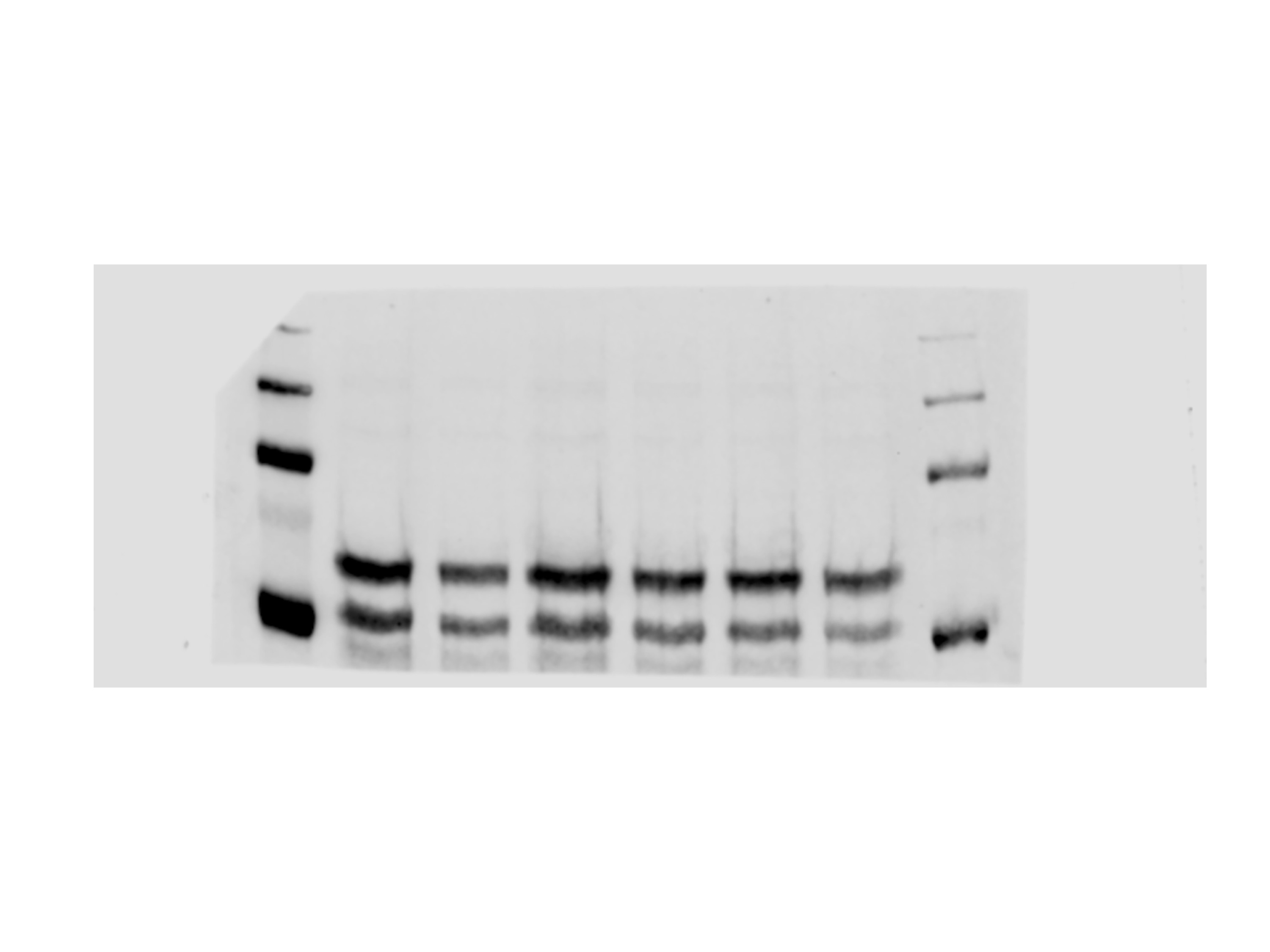

Supplement: Figure 6—source data 1. [file elife-79736-fig6-data1.zip › Figure 6-source data 1/Figure 6a_p53pT55.jpg]

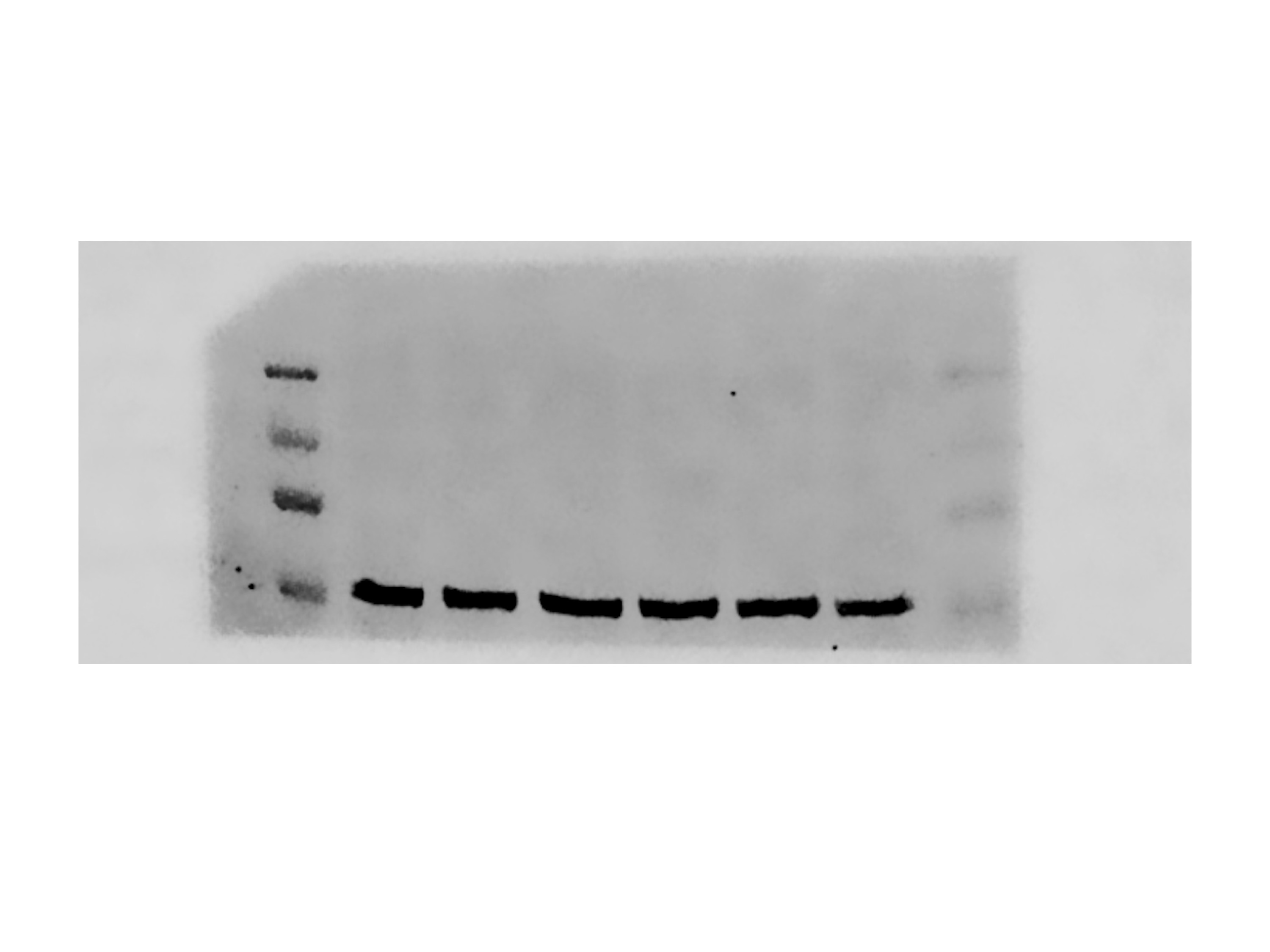

Supplement: Figure 6—source data 1. [file elife-79736-fig6-data1.zip › Figure 6-source data 1/Figure 6a_p53.jpg]

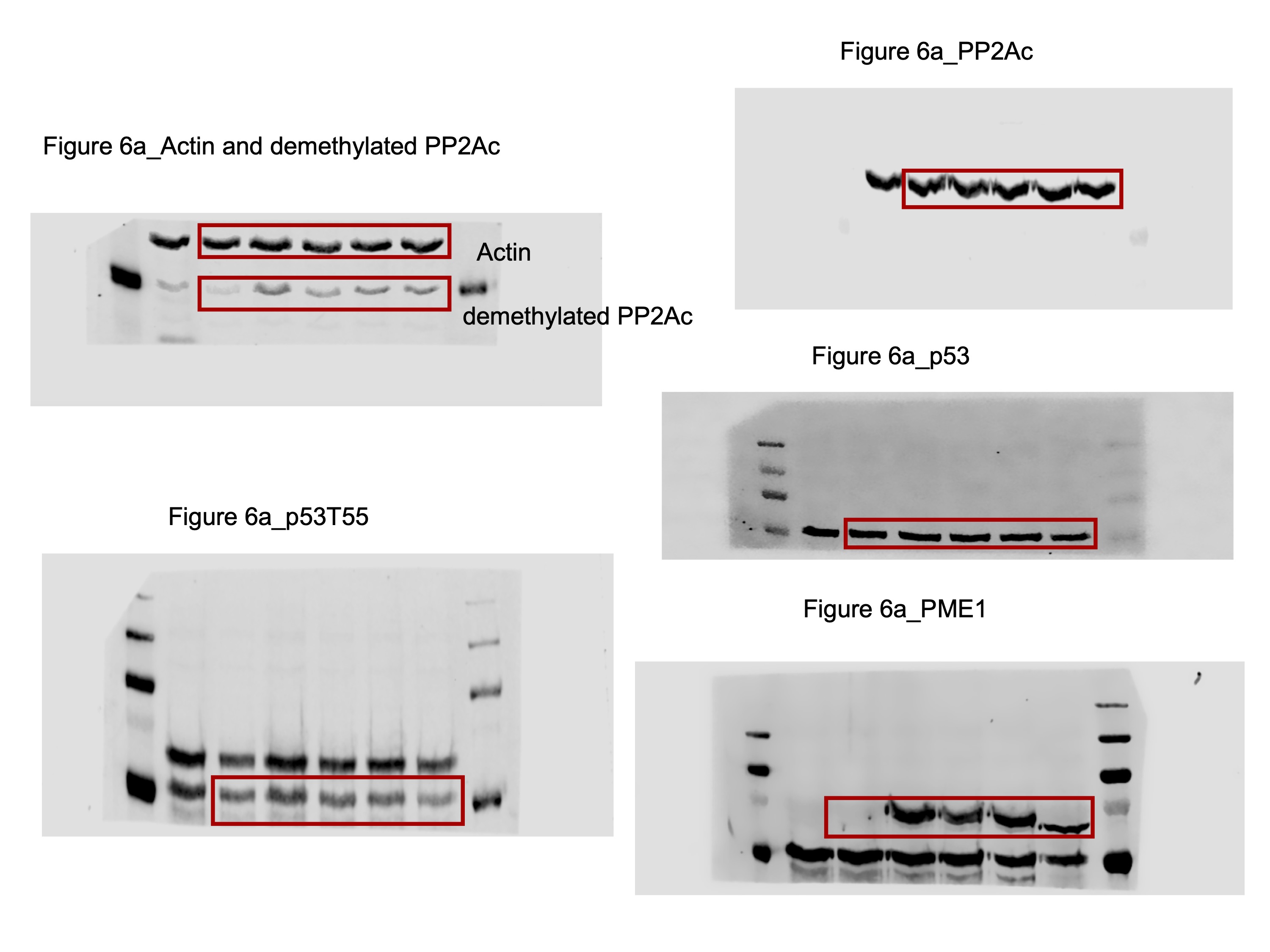

Supplement: Figure 6—source data 1. [file elife-79736-fig6-data1.zip › Figure 6-source data 1/Uncropped_Labeled_Gel_Figure 6a.jpg]

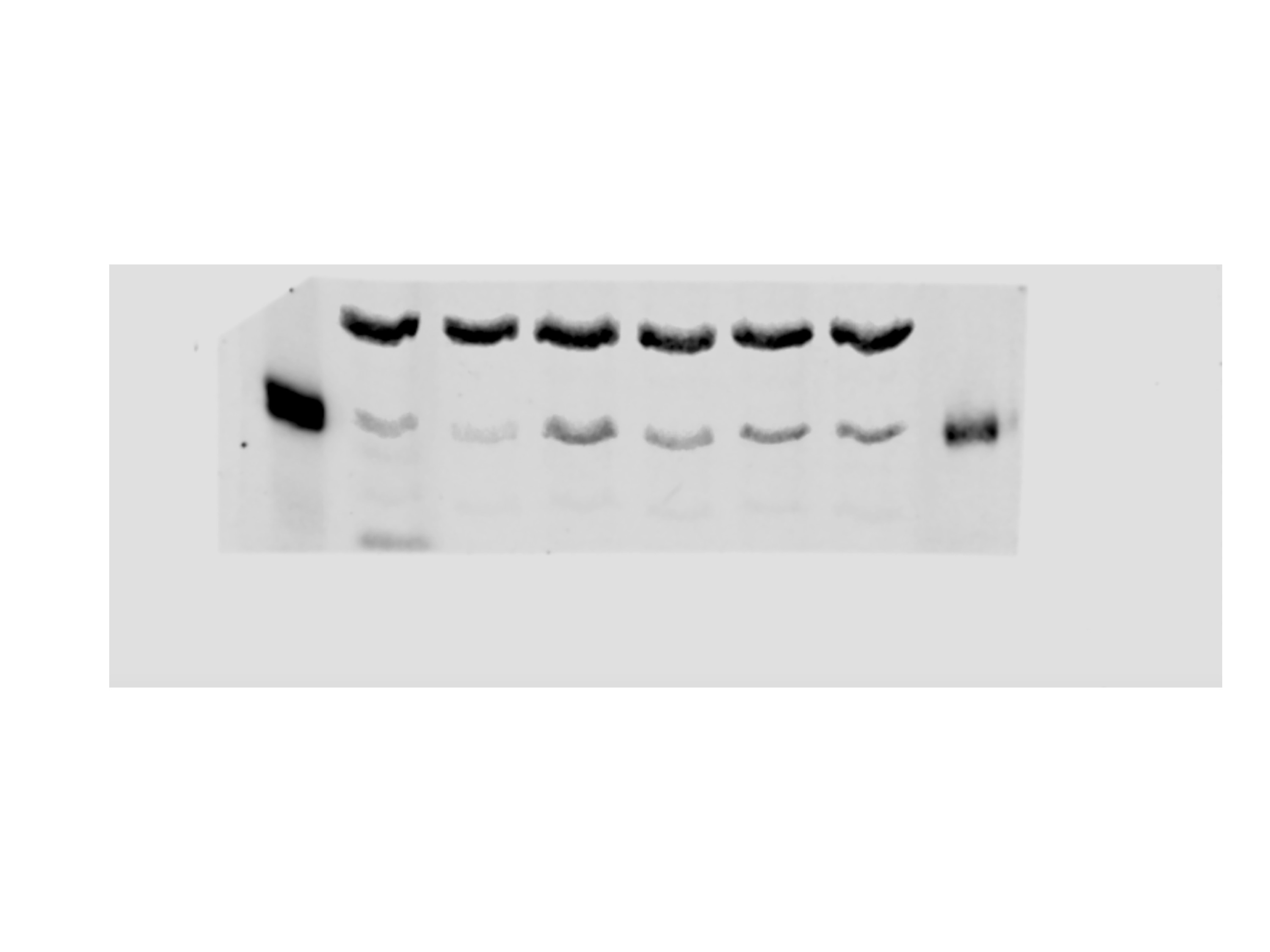

Supplement: Figure 6—source data 1. [file elife-79736-fig6-data1.zip › Figure 6-source data 1/Figure 6a_Actin and demethylated PP2Ac.jpg]

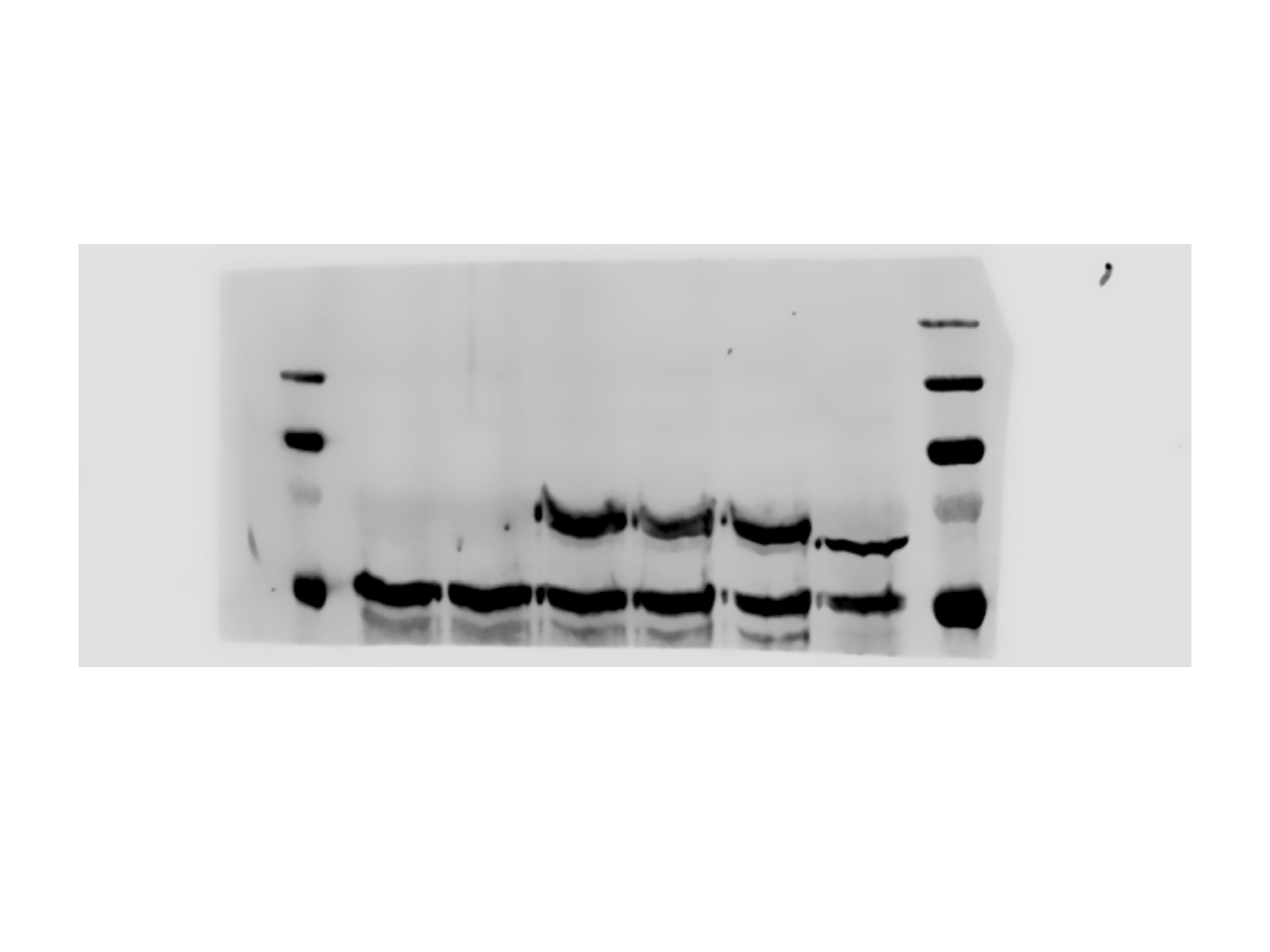

Supplement: Figure 6—source data 1. [file elife-79736-fig6-data1.zip › Figure 6-source data 1/Figure 6a_PME1.jpg]

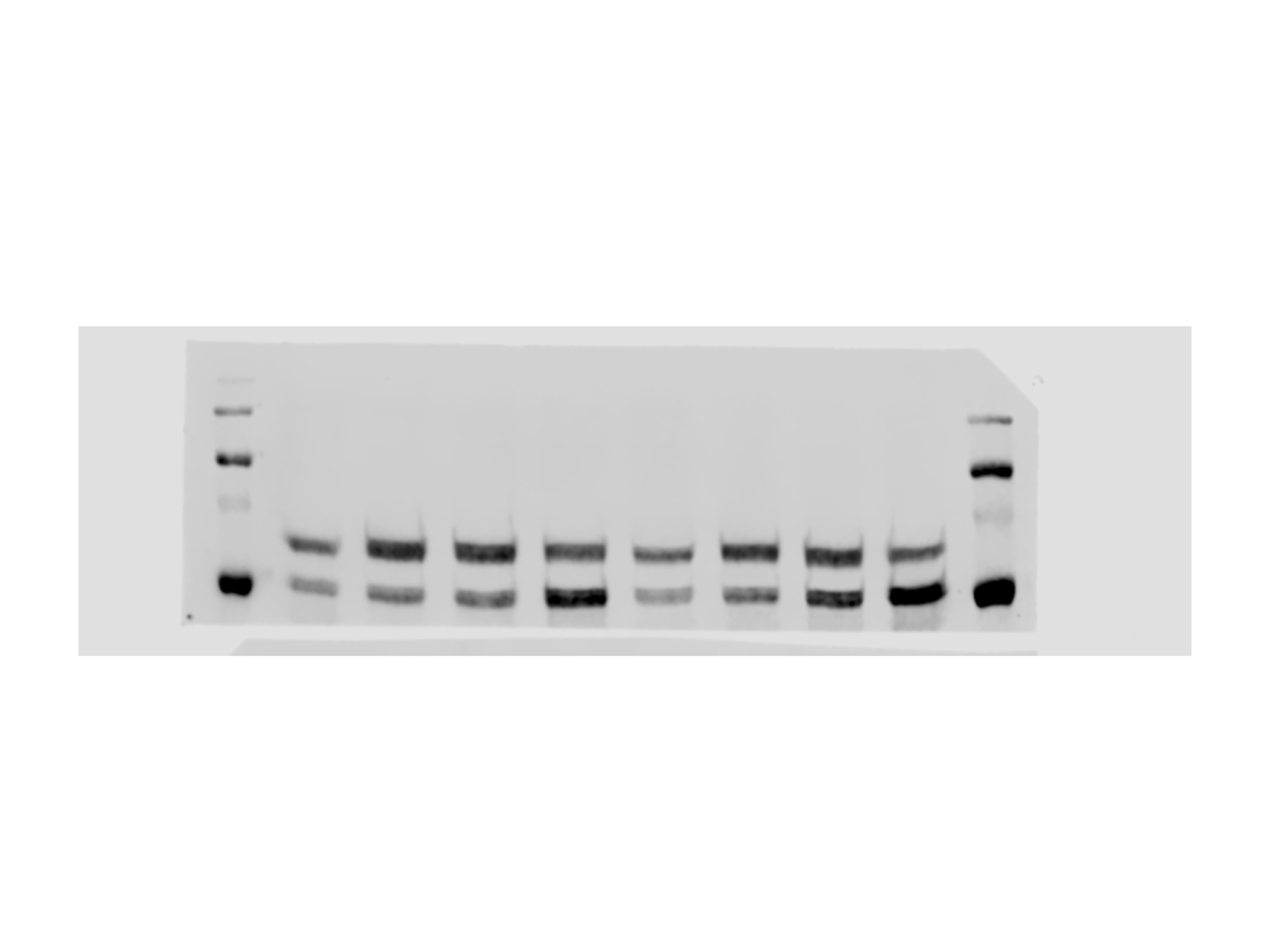

Supplement: Figure 6—source data 3. [file elife-79736-fig6-data3.zip › Figure 6-source data 3/Figure 6c_p53.jpg]

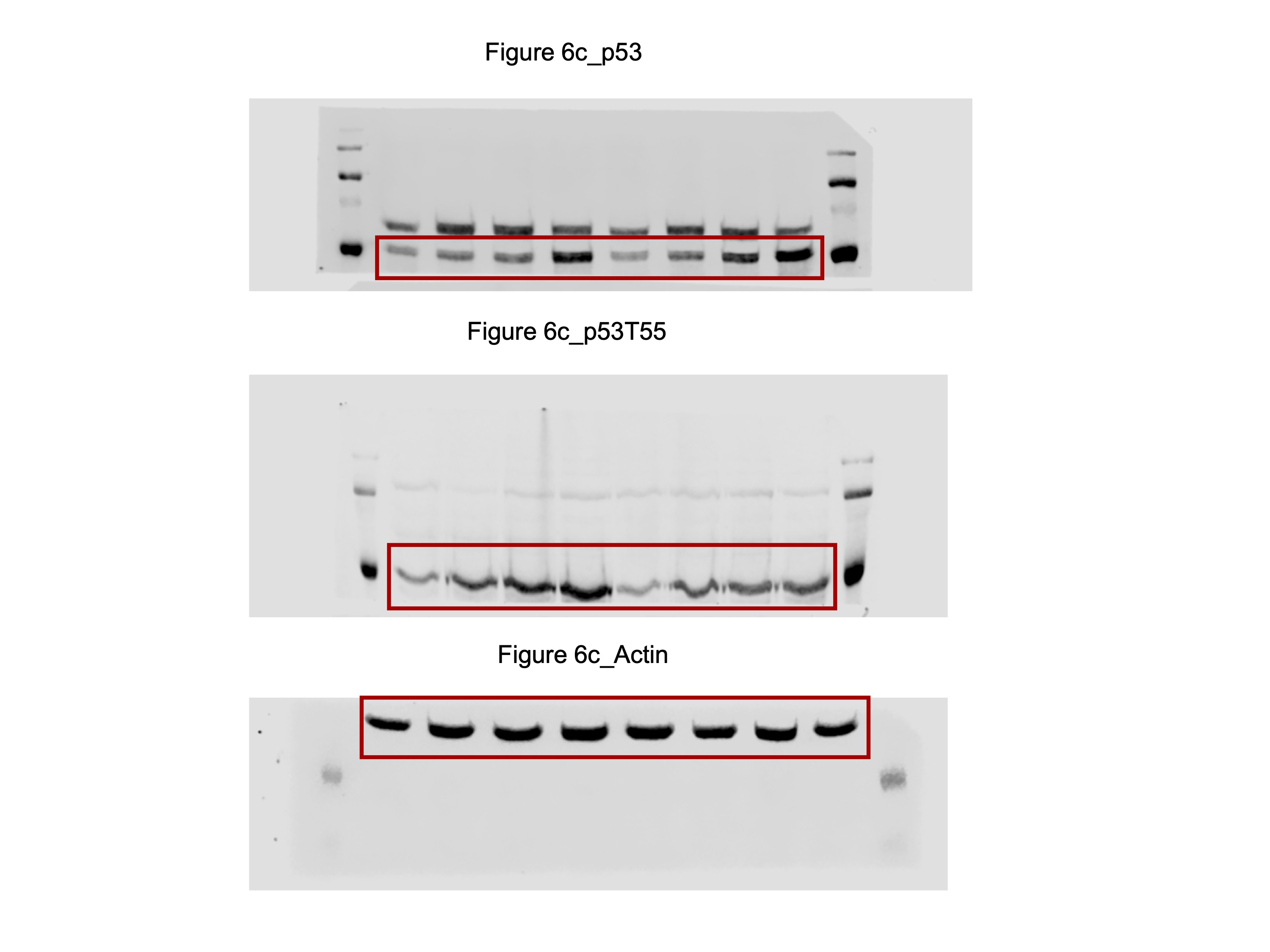

Supplement: Figure 6—source data 3. [file elife-79736-fig6-data3.zip › Figure 6-source data 3/Uncropped_Labeled_Gel_Figure 6c.jpg]

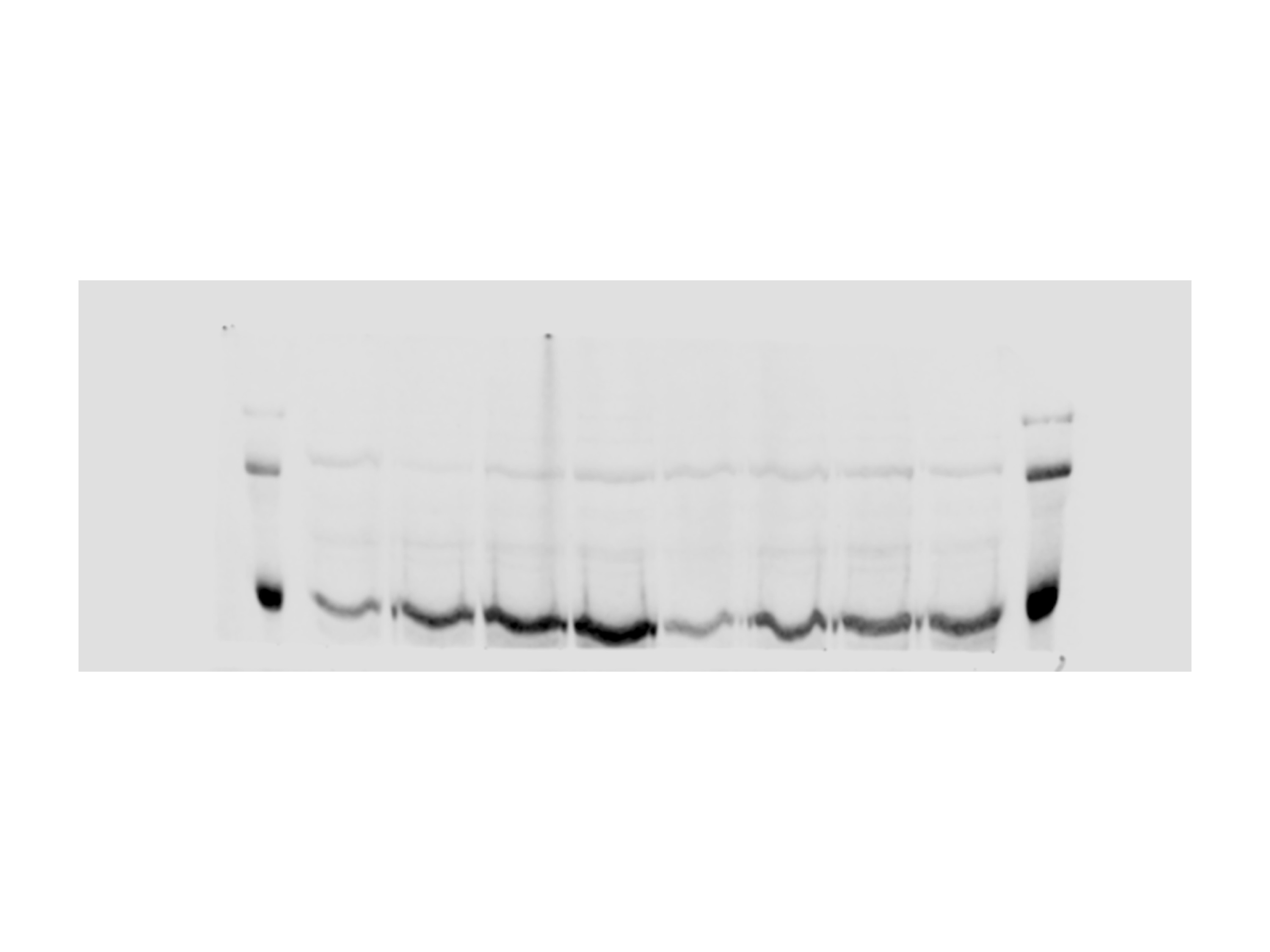

Supplement: Figure 6—source data 3. [file elife-79736-fig6-data3.zip › Figure 6-source data 3/Figure 6c_p53pT55.jpg]

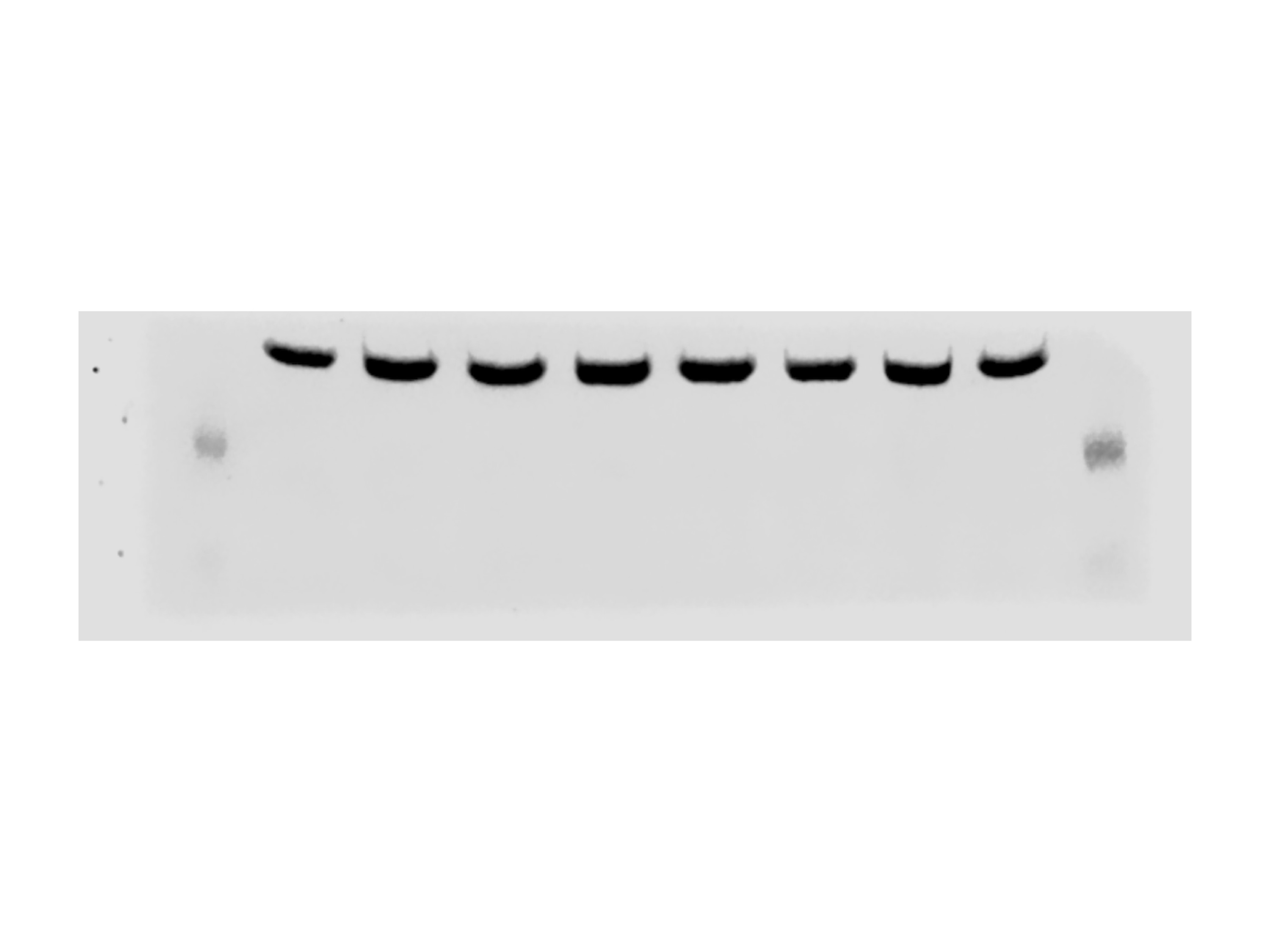

Supplement: Figure 6—source data 3. [file elife-79736-fig6-data3.zip › Figure 6-source data 3/Figure 6c_Actin.jpg]

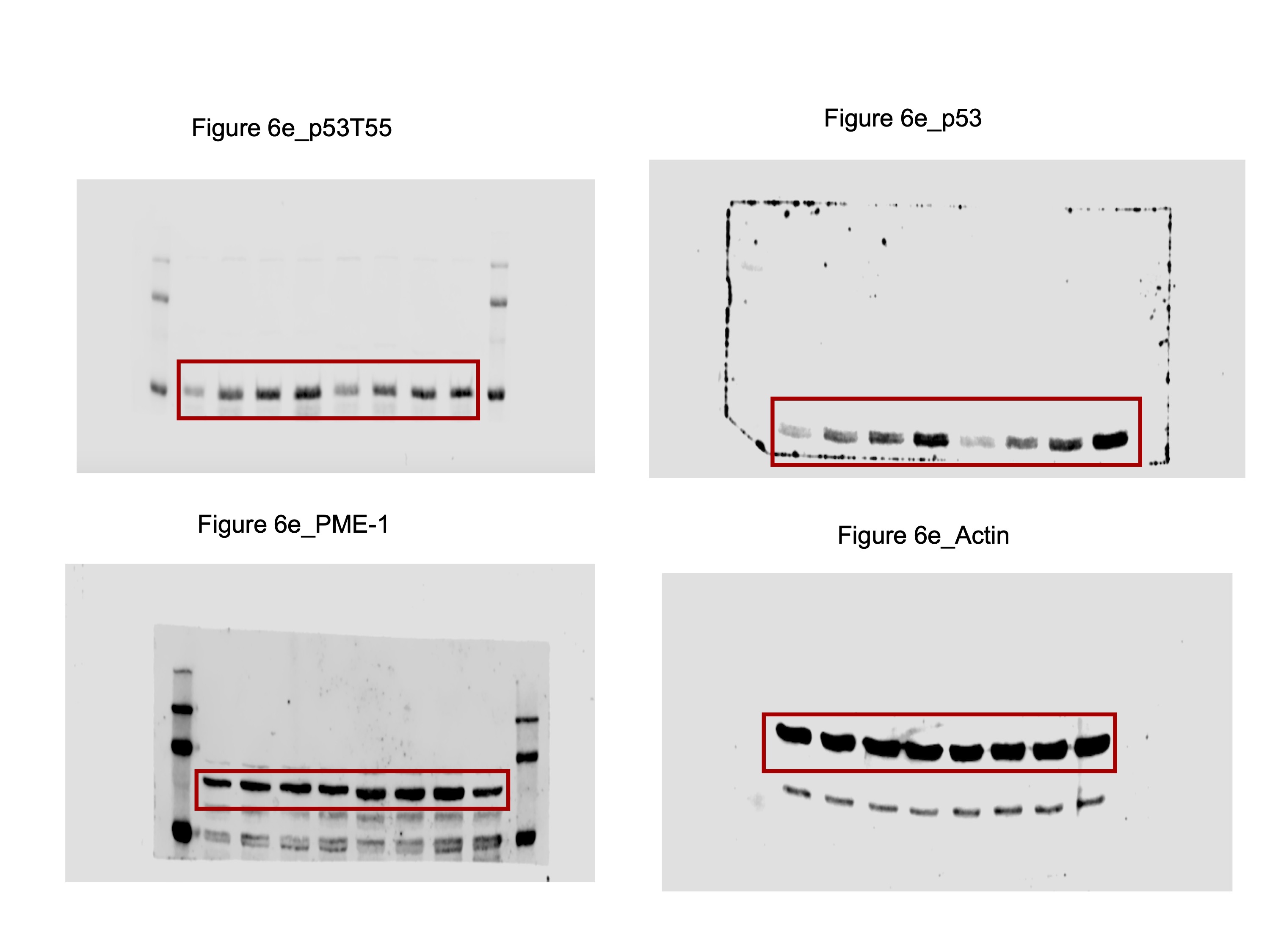

Supplement: Figure 6—source data 5. [file elife-79736-fig6-data5.zip › Figure 6-source data 5/Uncropped_Labeled_Gel_Figure 6e.jpg]

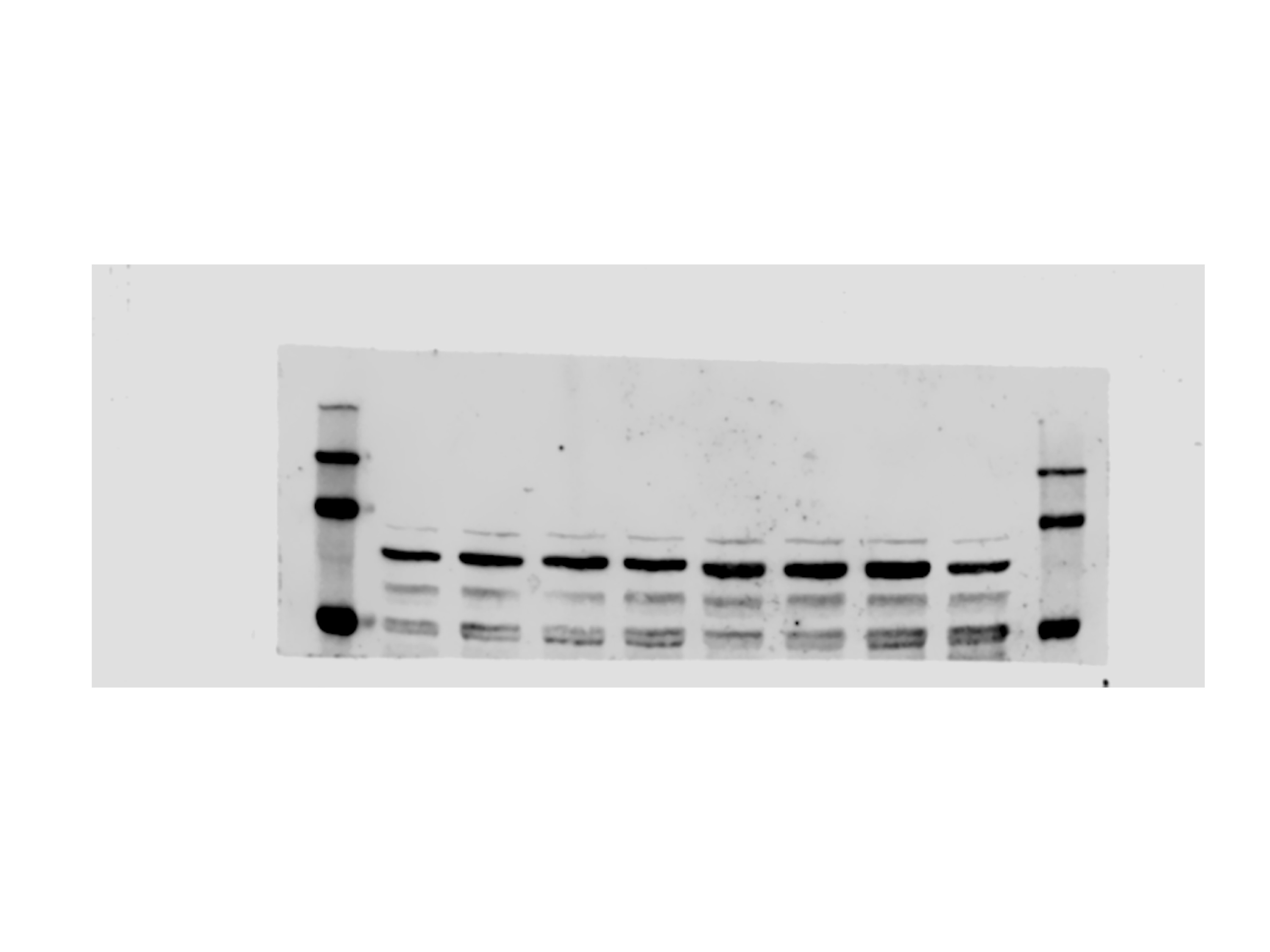

Supplement: Figure 6—source data 5. [file elife-79736-fig6-data5.zip › Figure 6-source data 5/Figure 6e_PME-1.jpg]

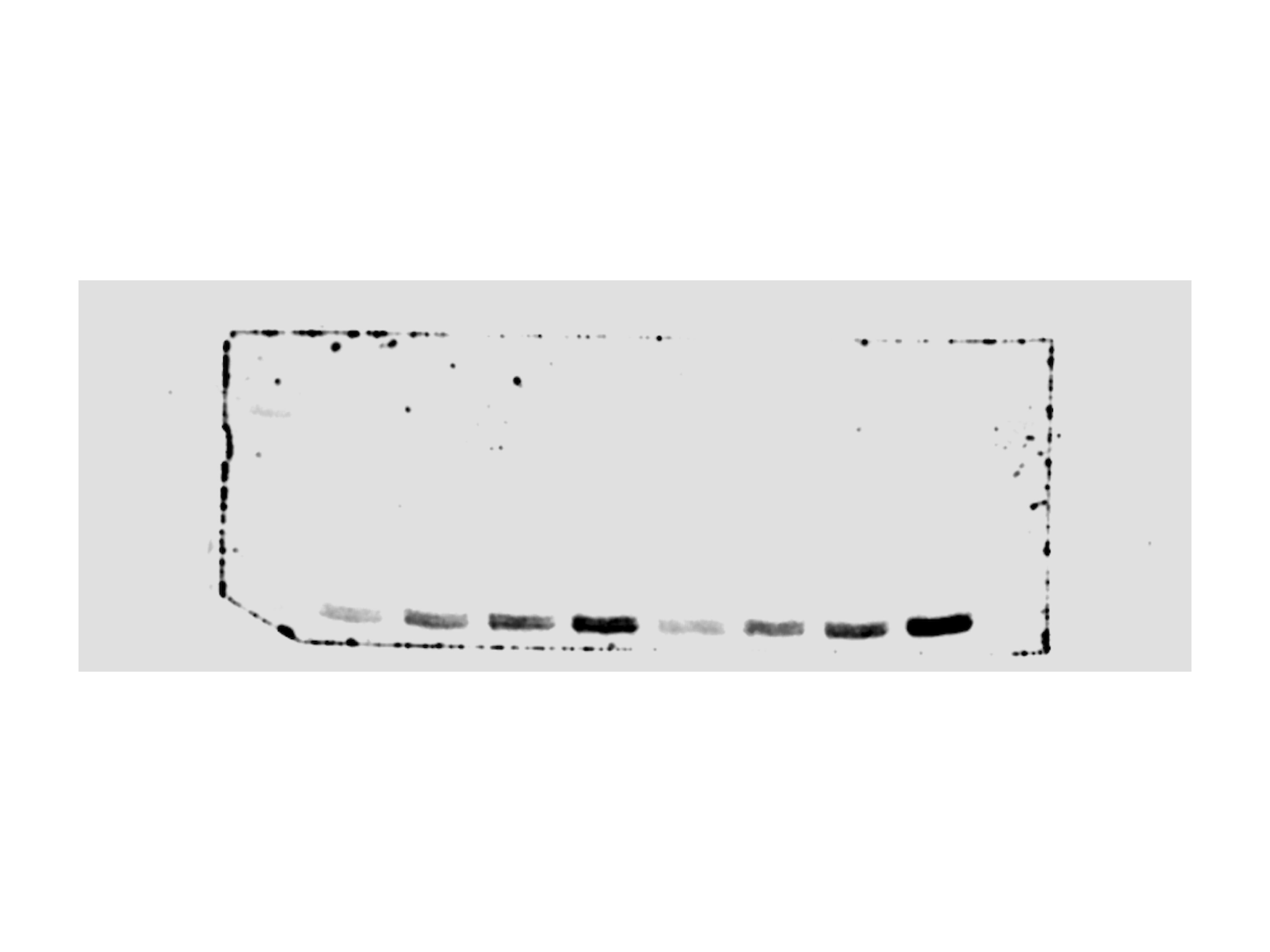

Supplement: Figure 6—source data 5. [file elife-79736-fig6-data5.zip › Figure 6-source data 5/Figure 6e_p53.jpg]

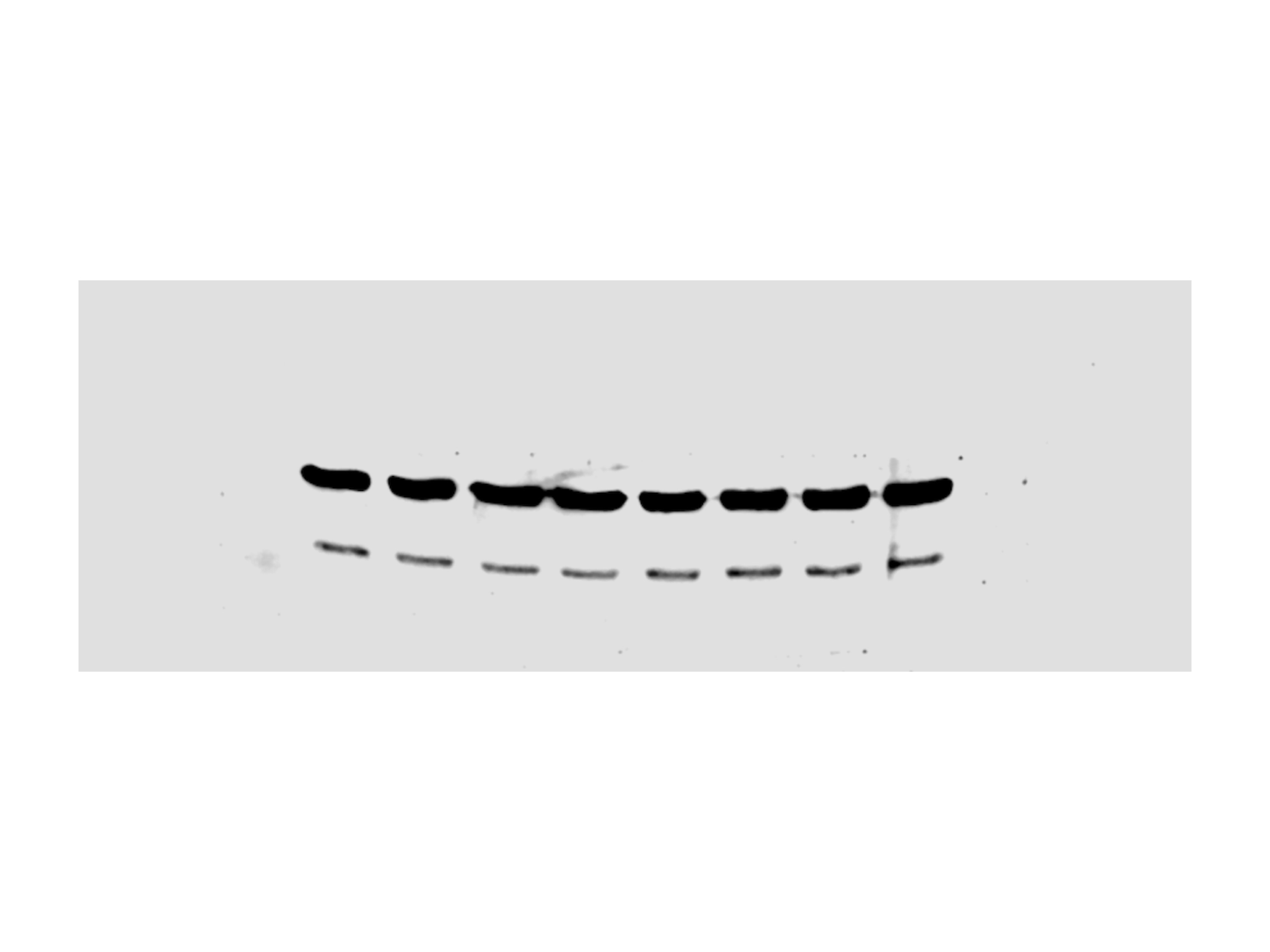

Supplement: Figure 6—source data 5. [file elife-79736-fig6-data5.zip › Figure 6-source data 5/Figure 6e_Actin.jpg]

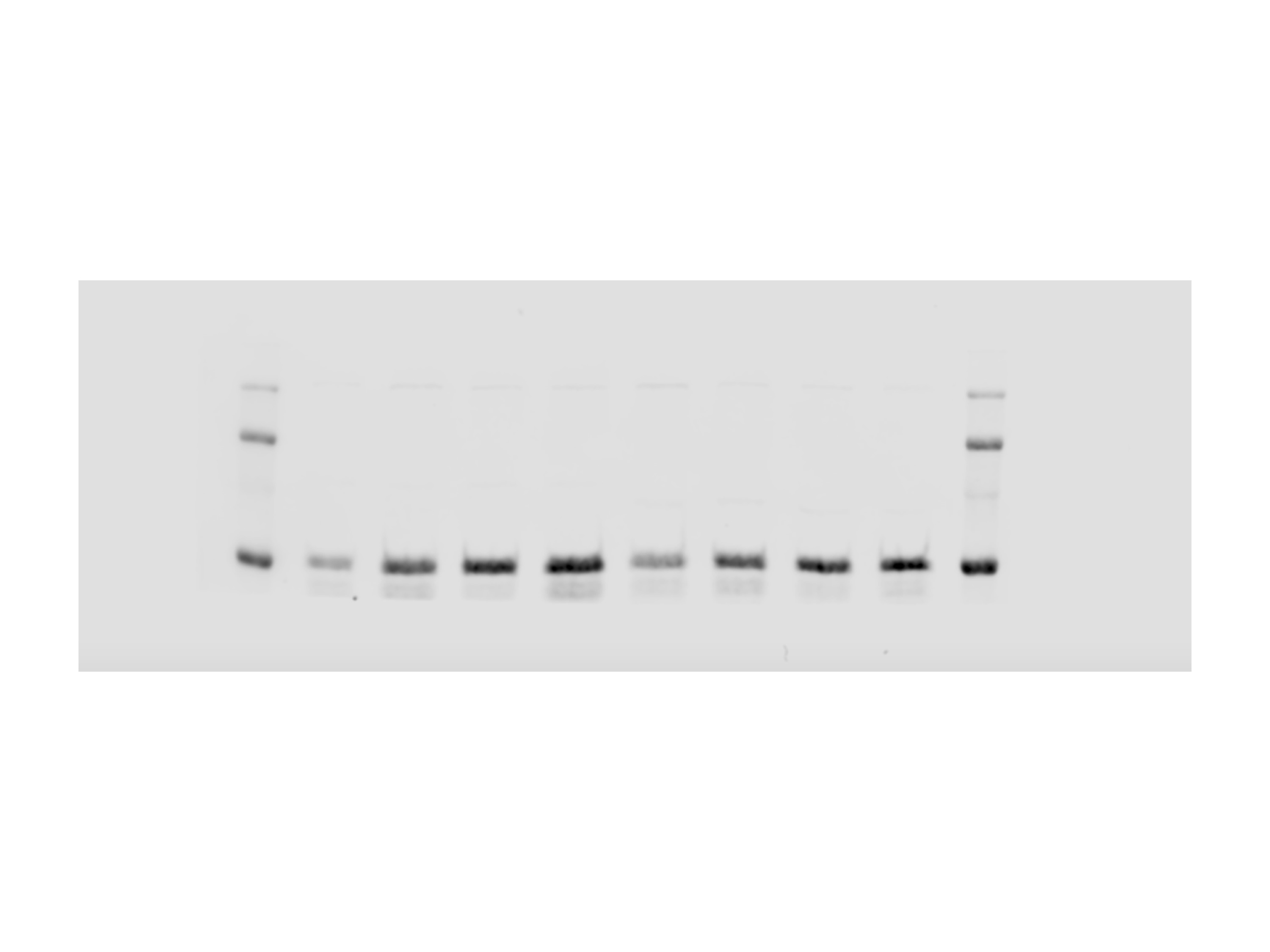

Supplement: Figure 6—source data 5. [file elife-79736-fig6-data5.zip › Figure 6-source data 5/Figure 6e_p53pT55.jpg]
